# Exploration of Indole Alkaloids from Marine Fungus *Pseudallescheria boydii* F44-1 Using an Amino Acid-Directed Strategy

**DOI:** 10.3390/md17020077

**Published:** 2019-01-23

**Authors:** Mei-Xiang Yuan, Yi Qiu, Yan-Qin Ran, Gong-Kan Feng, Rong Deng, Xiao-Feng Zhu, Wen-Jian Lan, Hou-Jin Li

**Affiliations:** 1 School of Chemistry, Sun Yat-sen University, Guangzhou 510275, China; yuanmx3@mail2.sysu.edu.cn (M.-X.Y.); qiuyi0771@163.com (Y.Q.); 2 School of Traditional Chinese Medicine, Guangdong Pharmaceutical University, Guangzhou 510006, China; RanyqGDPU@foxmail.com; 3 State Key Laboratory of Oncology in South China, Collaborative Innovation Center for Cancer Medicine, Cancer Center, Sun Yat-sen University, Guangzhou 510060, China; fenggk@sysucc.org.cn (G.-K.F.); dengrong@sysucc.org.cn (R.D.); zhuxfeng@mail.sysu.edu.cn (X.-F.Z.); 4 School of Pharmaceutical Sciences, Sun Yat-sen University, Guangzhou 510006, China

**Keywords:** *Pseudallescheria boydii*, indole alkaloid, pseudboindole, cytotoxic activity

## Abstract

The composition of the culture medium has great influence on the metabolite production of the marine fungus *Pseudallescheria boydii* F44-1. By adding amino acids to GPY culture medium, two new bisindole alkaloids, pseudboindoles A and B (**1** and **2**), together with 11 known indole alkaloids were isolated from the culture broth. Their structures were elucidated by comprehensive analysis of the NMR, MS, IR, and UV spectra. The 3,3′-cyclohexylidenebis(1*H*-indole) (**3**) showed cytotoxic activity against various cancer cell lines.

## 1. Introduction

Marine indole alkaloids are an increasingly growing class of secondary metabolites. From 2003 to 2015, about 800 new marine indole alkaloids were obtained [1]. Indole alkaloids—including mono-, bis- and trisindole alkaloids—are biosynthetically derived from one-, two- and three-indole building blocks, respectively, and have high structural diversity. Some of them were found to possess diverse biological activity, such as cytotoxic, antiviral, antiplasmodial, antifungal, antibacterial and anti-inflammatory activity, and are therefore promising leads for new drug development [2,3,4,5]. The natural occurrence of indole alkaloids is the result of biosynthesis via the coupling of the inessential amino acid tryptophan with other amino acids and structural fragments. Based on this consideration, our research group established an amino acid-directed strategy to explore the potential of marine fungi to produce diverse alkaloids. To date, more than forty novel and/or bioactive indole alkaloids have been obtained from marine fungi. For example, when cultured in glucose–peptone–yeast (GPY) extract medium supplemented with L-tryptophan, L-phenylalanine, L-threonine, and D,L-methionine, the marine fungus *Scedosporium apiospermum* F41-1 produced 12 new indole alkaloids. Among them, scedapin C and scequinadoline D displayed significant antiviral activity against hepatitis C [6]. A total of 18 indole alkaloids were isolated from the marine fungus *Dichotomomyces cejpii* F31-1 by feeding it with L-tryptophan and L-phenylalanine [7]. Scequinadoline A possesses the potential for further development as a dengue virus inhibitor [8].

*Pseudallescheria* species are filamentous fungi widely distributed in nature. The literature on the secondary metabolites of this fungal genus is still limited. In our previous research on marine fungi, three fungal strains were collected from marine organisms, and their metabolites showed chemodiversity and biodiversity. Two isobenzofuranone derivatives, pseudaboydins A and B [9], two new chlorinated benzofuran derivatives, 6-chloro-2-(2-hydroxypropan-2-yl)-2,3-dihydro-5-hydroxybenzofuran and 7-chloro-2-(2-hydroxypropan-2-yl)-2,3-dihydro-5-hydroxybenzofuran [10], were isolated from *Pseudallescheria boydii*, associated with the starfish *Acanthaster planci*. Pseudaboydin A showed moderate cytotoxic activity [9]. Two aromadendrane-type sesquiterpene diastereomers pseuboydones A and B, two diketopiperazines pseuboydones C and D, and a cyclopiazonic acid analogue pseuboydone E were isolated from the culture broth of the marine fungus *Pseudallescheria boydii* F19-1, which is associated with the soft coral *Lobophytum crassum*. Pseuboydone C displayed significant cytotoxicity against Sf9 cells from the fall armyworm *Spodoptera frugiperda* [11]. The pseudellones A−D [12] and (5S,6S)-dihydroxylasiodiplodin [13] were isolated from the marine fungus *Pseudallescheria ellipsoidea* F42-3, associated with the soft coral *Lobophytum crissum*.

**Figure 1:**
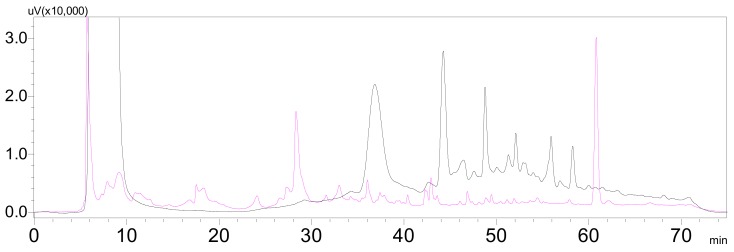
HPLC profiles detected at 254 nm of the metabolite extracts of marine fungus *Pseudallescheria boydii* F44-1 cultured in GPY medium (pink line) and GPY medium supplemented with various amino acids (black line).

**Figure 2:**
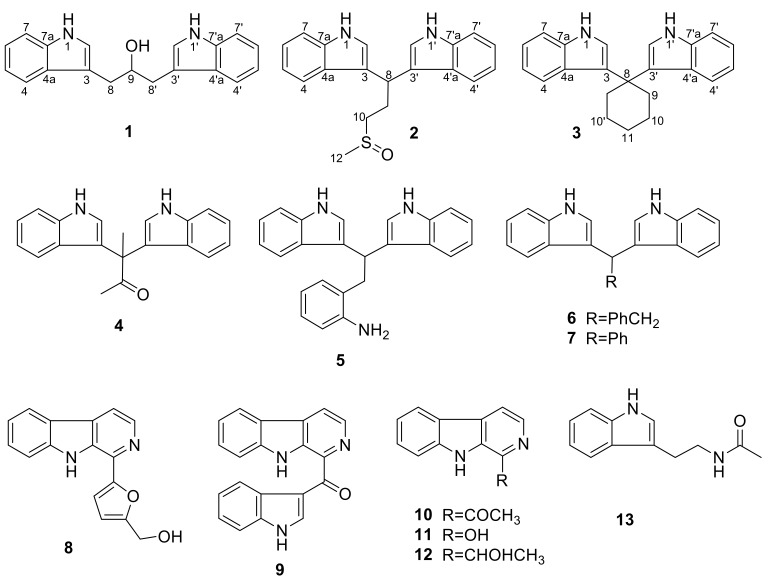
Chemical structure of compounds **1−13.**

Recently, another marine fungus *Pseudallescheria boydii* (collection no. F44-1) was isolated from the soft coral *Sarcophyton* sp. collected in the Hainan Sanya National Coral Reef Reserve, China. This fungal strain was cultured in GPY medium and GPY medium supplied with amino acids, including L-tryptophan, L-phenylalanine, L-methionine, and L-threonine. The culture extracts were analyzed with HPLC detected at UV 254 nm. HPLC traces indicated that *Pseudallescheria boydii* F44-1 cultured in the GPY medium containing additional amino acids could produce more metabolites with strong UV absorption (Figure 1 and Supplementary Figure S1). This meant that amino acids could regulate the production of metabolites containing aromatic rings. By tracking the characteristic ^1^H NMR signals in the aromatic region 6.5−8.5 ppm, two new bisindole alkaloids pseudboindoles A and B (**1** and **2**), together with 11 known indole alkaloids (Figure 2), were obtained efficiently. Here, we reported the isolation, structure elucidation and cytotoxic activity of these compounds.

## 2. Results and Discussion

### 2.1. Structural Elucidation

**Figure 3:**
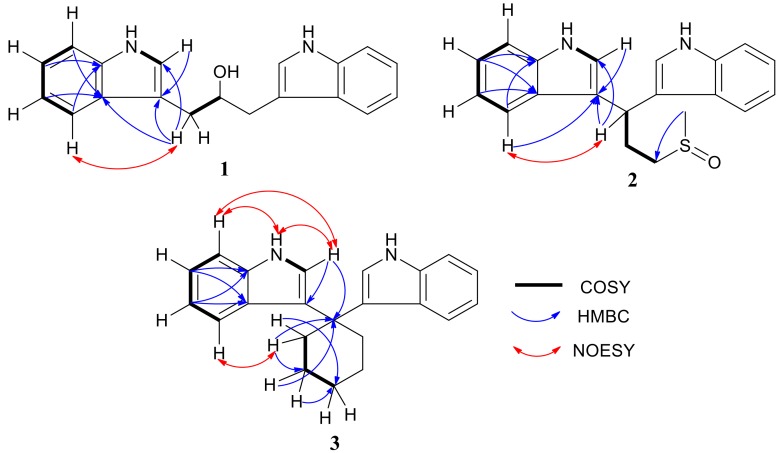
^1^H−^1^H COSY (bold lines), main HMBC (blue arrows) and NOESY (red arrows) correlations of compounds **1**–**3**.

Pseudboindole A (**1**) was obtained as a brown amorphous powder. The molecular formula was determined to be C_19_H_18_N_2_O by HR-(+)ESI-MS at *m/z* 291.14789 [M + H]^+^ (calculated for C_19_H_19_N_2_O, 291.14919) (Supplementary Figure S2), which has 12 degrees of unsaturation. The IR spectrum indicated the presence of the hydroxy group (3409 cm^−1^) and benzene ring (1618 and 1456 cm^−1^). UV maxima at 222 and 282 nm also displayed the conjugated system containing a benzene ring. The ^13^C NMR and DEPT showed one methylene, six methines and three quaternary carbons (Table 1 and Supplementary Figures S4–S6). The ^1^H NMR spectrum showed a set of adjacent aromatic protons at δ_H_ 7.61 (brd, 8.0, H-4), 7.34 (brd, 8.0, H-7), 7.21 (ddd, 8.0, 8.0, 0.8, H-6) and 7.12 (ddd, 8.0, 8.0, 0.8, H-5) (Supplementary Figure S3), indicating the existence of an ortho-disubstituted aromatic ring. Besides, the ^1^H NMR spectrum also displayed one methylene group (δ_H_ 3.09, dd, 14.4, 4.8; 2.95, dd, 14.4, 8.0), one methine group (δ_H_ 4.30, dddd, 8.0, 8.0, 4.8, 4.8, H-9), one hydroxyl group (δ_H_ 2.08, brs), and a broad singlet (δ_H_ 8.10, brs, NH) (Supplementary Figure S7). The ^1^H−^1^H COSY cross-peaks of H-1/H-2, H-4/H-5/H-6/H-7 (Figure 3 and Supplementary Figure S8) and the HMBC correlations from H-4 to C-7a (δ_C_ 136.3), H-7 to C-4a (δ_C_ 127.6), H-2 to C-3 (δ_C_ 112.4) (Supplementary Figure S9) were indicative of the presence of a 3-substitued indole alkaloid skeleton. In addition, the ^1^H−^1^H COSY correlations of H-8/H-9 demonstrated the presence of a −CH_2_CH− moiety. C-3 was connected to C-8 based on the HMBC correlations of H-8 with C-2/C-3/C-4a, and the NOESY correlation of H-4/H-8 (Supplementary Figure S10). The remaining hydrogen deficiency index further determined that two identical structural moieties were connected to C-9 (δ_C_ 71.5). Consequently, the chemical structure of pseudboindole A (**1**) was illustrated as 1,3-di(1*H*-indol-3-yl)propan-2-ol (Figure 1).

The molecular formula of pseudboindole B (**2**) was revealed to be C_20_H_20_N_2_OS by HR-(−)ESI-MS at *m/z* 335.12247 [M−H]^−^ (Supplementary Figure S11) requiring 13 degrees of unsaturation. IR absorption at 1659 and 1419 cm^−1^ and UV maxima at 222 and 282 nm also indicated the existence of the benzene ring conjugated system. The ^13^C NMR and DEPT spectra (Supplementary Figures S13–S15) displayed one methyl, two methylenes, six methines and three quaternary carbons (Table 1). In the ^1^H NMR spectrum (Supplementary Figure S12), the integral ratios of aryl proton, methylene, and methyl were 1:1:1.5 and indicated that the molecular structure was symmetrical. The ^1^H NMR spectrum displayed aromatic protons at δ_H_ 7.55 (dd, 7.6, 7.6, H-5), 7.34 (d, 8.0, H-7), 7.16 (dd, 8.0, 7.6, H-6) and 7.04 (d, 7.6, H-4) and ^1^H−^1^H COSY correlations of H-4/H-5/H-6/H-7 (Figure 3) revealed the existence of an ortho-disubstituted aromatic ring. Additionally, the ^1^H−^1^H COSY correlations of H-1/H-2 (Supplementary Figure S17) and the key HMBC correlations of H-4 (H-6)/C-7a (δ_C_ 136.60 or 136.56), H-5 (H-7)/C-4a (δ_C_ 126.80 or 126.71), and H-2/C-4a/C-7a/C-3 (δ_C_ 118.67 or 118.40) (Supplementary Figures S16 and S18) indicated a 3-substitued indole skeleton. The ^1^H−^1^H COSY cross peaks of H-8/H-9/H-10 revealed the fragment of −CHCH_2_CH_2_−. The remaining methyl singlet signal at δ_H_ 2.47 (H_3_-12) was connected to a sulfoxide group, which can enable the further analysis of the HMBC correlation of H_3_-12 to C-10 (δ_C_ 53.0). Consequently, the chain partial structure of −CHCH_2_CH_2_SOCH_3_ was established. The HMBC correlations of H-8/C-3 indicated that two 3-substitued indole parts were connected to C-8 (δ_C_ 33.2), which was confirmed by the NOESY correlation of H-4/H-8 (Supplementary Figure S19). Therefore, the chemical structure of pseudboindole B (**2**) was unambiguously established, as shown in Figure 2.

Compound **3** was a brown amorphous powder. It had the molecular formula C_22_H_22_N_2_, which was established on the basis of the HR-(−)ESI-MS ion at *m/z* 313.17120 [M−H]^−^ (calcd. for C_22_H_21_N_2_, 313.17102) (Supplementary Figure S20) and indicated 13 degrees of unsaturation. The 1D and 2D NMR data recorded in CDCl_3_ and acetone-*d*_6_ were slightly different (Table 2 and Supplementary Figures S21–S33). The ^13^C NMR and DEPT spectra displayed three methylenes, five methines and four quaternary carbons. Compared with the NMR data of pseudboindoles A and B, the fragment of 3-substitued indole also existed. The ^1^H-^1^H COSY cross peaks of H-9/H-10/H-11 (Figure 3) revealed the remaining three methylene forming the fragment of −CH_2_CH_2_CH_2_−. However, the integrals of H-9, H-10, H-11 and aromatic proton were 2:2:1:1. Based on the above analysis, compound **3** was inferred containing a symmetric framework and belonged to the bisindole class. The analysis of the HMBC correlations of H-2 (δ_H_ 7.10, d, 1.8)/C-8 (δ_C_ 39.5), H-9 (δ_H_ 2.55, t, 6.0)/C-8, H-10 (δ_H_ 1.66, m)/C-8, the two 3-substitued indole parts and the chain part of −CH_2_CH_2_CH_2_CH_2_CH_2_− were connected to C-8. The chemical structure of compound **3** was illustrated as 3,3′-cyclohexylidenebis(1*H*-indole), as seen in Figure 2. The 3,3′-cyclohexylidenebis(1*H*-indole) (**3**) can be synthesized by the reaction of an indole with cyclohexanone, and showed rather potent enhancing activity (140%) on Am80-induced HL-60 cell differentiation [14,15]. This is the first report of compound **3** as a natural product and its ^1^H and ^13^C NMR data assignment were elaborated unambiguously.

Compounds **4**–**13** were identified as 3,3-bis(3-indolyl)butan-2-one (**4**) [16], 2-[2,2-di(1*H*-indol-3-yl) ethyl] aniline (**5**) [17], 3,3′-diindolyl(phenyl)methane (**6**) [18], 1,1-(3,3′-diindolyl)-2-phenylethane (**7**) [18], perlolyrin (**8**) [19], pityriacitrin (**9**) [20], 1-acetyl-β-carboline (**10**) [21], 3-hydroxy-β-carboline (**11**) [22], 1-(9*H*-pyrido[3,4-b]indol-1-yl)ethan-1-ol (**12**) [23], and N_b_-acetyltryptamine (**13**) [24], respectively, by comparing their spectroscopic data (Supplementary Figures S34–S74) with the literature values.

### 2.2. Biological Activity

Eight cancer cell lines, including human lung cancer cell lines A549 and GLC82, human nasopharyngeal carcinoma cell lines CNE1, CNE2, HONE1 and SUNE1, human hepatoma carcinoma cell lines BEL7402 and SMMC7721, were used to evaluate the cytotoxic activities of **1**−**13** in vitro. As a result, compound **3** showed significant cytotoxicity against these cancer cell lines A549, GLC82, CNE1, CNE2, HONE1, SUNE1, BEL7402, and SMMC7721 with the IC_50_ values of 22.84, 22.04, 18.69, 20.84, 26.62, 20.54, 27.52 and 22.50 μM, respectively. In contrast, **1**, **2** and **4**−**13** were apparently inactive in this assay (IC_50_ > 200 μM).

## 3. Materials and Methods

### 3.1. General Procedures

The column chromatography made use of silica gel (SiO_2_, 200−300 mesh, Qingdao Marine Chemical Inc., Qingdao, China). Preparative HPLC was performed using a Shimadzu LC-20AT HPLC pump (Shimadzu Corporation, Nakagyo−ku, Kyoto, Japan) and installed with an SPD-20A dual λ absorbance detector (Shimadzu Corporation, Nakagyo−ku, Kyoto, Japan) and a Capcell−Pak C18 UG80 HPLC column (250 mm×20 mm, Shiseido Co., Ltd., Minato-ku, Tokyo, Japan) and a Spolar HPLC packed column (250 mm × 4.6 mm, Shiseido Co., Ltd., Minato-ku, Tokyo, Japan). The melting point used the melting point apparatus WRS-3 (Shenguang, Shanghai, China) to record. UV data were obtained on a Shimadzu UV-Vis-NIR spectrophotometer (Shimadzu Corporation, Nakagyo-ku, Kyoto, Japan). IR spectra were recorded on a PerkinElmer Frontier FT-IR spectrophotometer (PerkinElmer Inc., Waltham, MA, USA). The 1D and 2D NMR experiments were measured with Bruker Avance 400 spectrometer and Bruker Avance 600 spectrometer (Bruker Bio Spin AG, Industriestrasse 26, Fällanden, Switzerland). The chemical shifts were relative to the residual solvent signals (CDCl_3_: δ_H_ 7.260 and δ_C_ 77.000; acetone-*d*_6_: δ_H_ 2.050 and δ_C_ 29.840; and methanol-*d*_4_: δ_H_ 3.310 and δ_C_ 49.000). HR-ESI-MS data were collected on a Thermo Fisher LTQ Orbitrap Elite high-resolution mass spectrometer (Thermo Fisher Scientific Inc., Waltham, MA, USA).

### 3.2. Fungal Strain and Culture Method

The marine fugus *Pseudallescheria boydii* (collection no. F44-1) was isolated from the inner tissue of the soft coral *Sarcophyton* sp. collected from Hainan Sanya National Coral Reef Reserve, China. This fungal strain was conserved in 15% (*v*/*v*) glycerol aqueous solution at −80 °C. A voucher specimen was deposited in the School of Chemistry, Sun Yat-sen University, Guangzhou, China. Analysis of the ITS rDNA by BLAST database screening provided 99.9% match to *Pseudallescheria boydii*.

The fermentation medium was glucose 15 g, peptone 10 g, yeast extract 2 g, L-tryptophan 2 g, L-phenylalanine 2 g, L-methionine 2 g, L-threonine 2 g, sea salt 25 g, and H_2_O 1L at pH 7.5. Fungal mycelia were cut and transferred aseptically to 1000 mL conical flasks each containing 400 mL sterilized liquid medium. The flasks were incubated at 28 °C for 20 days.

### 3.3. Extraction and Isolation

A total of 60 liters of liquid culture were filtered through cheesecloth. The culture broth was successively extracted five times with EtOAc (60 L). Finally, the extract was concentrated by low-temperature rotary evaporation to obtain a crude extract (39.8 g).

The extract was chromatographed on a silica gel column (diameter: 8 cm, length: 70 cm, silica gel, 450 g) with a gradient of petroleum ether−EtOAc (100:0−0:100, *v*/*v*) followed by EtOAc−MeOH (100:0−0:100, *v*/*v*) to yield thirty fractions (Fr.1−Fr.30). Fr. 15−Fr.19 were merged for having the similar fractions as monitored by ^1^H NMR prescreening, and then, the constituents was purified by silica gel column using a step gradient elution with petroleum ether−EtOAc (10:0−0:10, *v*/*v*) to obtain 8 subfractions (Fr.15-19-1−Fr.15-19-8). Compound **1** (18.3 mg) was obtained from Fr. 15-19-6 by repeated preparative HPLC using CH_3_CN−H_2_O (60:40, *v*/*v*, RT = 37.5 min) as eluent. HPLC purification of Fr. 9 with a solvent system CH_3_OH−H_2_O (75:25, *v/v*, RT = 23.5 min) gave compound **2** (16.5 mg). Fr.5 was purified by preparative HPLC with a mobile phase of MeOH-H_2_O (75:25, *v/v*, RT = 54 min) to obtain compound **3** (15.3 mg). Fr.12 was purified with preparative HPLC (CH_3_CN−H_2_O, 80:20, *v*/*v*, RT = 27 min) to obtain compound **4** (5.3 mg). Fr.10–Fr.11 was merged after ^1^H NMR prescreening, and then were purified with preparative HPLC using CH_3_CN−H_2_O (85:15, *v*/*v*) as eluent to obtain compounds **5** (RT = 28 min, 6.1 mg), **6** (RT = 34 min, 1.5 mg), **7** (RT = 35 min, 1.0 mg), and **9** (RT = 52 min, 5.6 mg). Similarly, Fr.21 was purified with preparative HPLC and eluted with CH_3_CN−H_2_O (70:30, *v*/*v*) to obtain compound **8** (RT = 27 min, 20.0 mg). Compound **10** (12.3 mg) was purified from Fr.7 with preparative HPLC (CH_3_OH−H_2_O, 75:25, *v*/*v*, RT = 47.5 min). Fr.23-Fr.24 was further purified using CH_3_OH−H_2_O (75:25, *v*/*v*) as eluent and got compounds **11** (RT = 39 min, 5.5 mg), **12** (RT = 43 min, 5.2 mg) and **13** (RT =31 min, 5.4 mg).

Pseudboindole A (**1**). Brown amorphous powder. mp 116.2−116.8 °C. UV (MeOH) λ_max_ (log ε): 282 (3.73), 222 (4.45). IR υ_max_ 3409, 2924, 1678, 1618, 1456, 1340, 1227, 1094, 1050, 738 cm^-1^. ^1^H and ^13^C NMR data, see Table 1; HR-(+) ESI-MS *m/z* 291.14789 [M + H]^+^ (calcd. for C_19_H_19_N_2_O, 291.14919).

Pseudboindole B (**2**). Brown amorphous powder. mp 113.9−114.8 °C. UV (MeOH) λ_max_ (log ε): 282 (3.82), 222 (4.51). IR υ_max_ 3420, 2980, 2926, 1659, 1419, 1332, 1197, 1094, 1050, 937, 881, 741 cm^-1^. ^1^H and ^13^C NMR data, see Table 1; HR-(−)ESI-MS *m/z* 335.12247 [M−H]^−^ (calcd. for C_20_H_19_N_2_OS, 335.12236).

3,3′-cyclohexylidenebis(1*H*-indole) (**3**). Brown amorphous powder. mp 114.7−115.9 °C. UV (MeOH) λ_max_ (log ε): 283 (3.88), 224 (4.63). IR υ_max_ 3405, 2935, 1686, 1618, 1456, 1415, 1097, 1336, 1242, 1103, 1046, 1012, 817, 738 cm^-1^. ^1^H and ^13^C NMR data, see Table 2; HR-(−) ESI-MS *m/z* 313.17120 [M−H]^−^ (calcd. for C_22_H_21_N_2_, 313.17102).

### 3.4. Cytotoxicity Assay

The in vitro cytotoxic activity of **1**−**13** was determined by means of the colorimetric MTT (3-(4,5-dimethylthiazol-2-yl)-2,5-diphenyl-2H-tetrazolium bromide) assay. The tested human cancer cell lines were seeded in 96-well plates at a density of 3 × 10^7^ cells/L, and the compounds were added at various concentrations (7.864−30.00 μM). After 72 h, MTT was added to the culture medium at a final concentration of 0.5 mg/mL, and the plates were incubated for 4 h at 37 °C. The supernatant was removed. The formazan crystals were dissolved in DMSO (150 μL) with gentle shaking at room temperature. The absorbance at 570 nm was recorded with a microplate reader (Bio-Rad, Hercules, CA, USA), and the data were analyzed with the SPSS (version 13.0) [25].

## 4. Conclusions

By tracking characteristic ^1^H NMR signals in the aromatic region of 6.50−8.50 ppm, two new bisindole compounds, pseudboindoles A and B (**1** and **2**), together with 11 known indole alkaloids (**3**−**12**) were efficiently isolated from the marine fungus *Pseudallescheria boydii* F44-1. The 3,3′-cyclohexylidenebis(1*H*-indole) (**3**) showed significant cytotoxic activity against various cancer cell lines. The result proves again that an amino acid-directed strategy is effective for inducing the marine fungi to produce diverse alkaloids. However, the specific quantitative relationship between amino acids and alkaloids and their biosynthesis pathways still need further study. After revealing these relationships, the application of this strategy will be more efficient.

## Figures and Tables

**Table 1:** ^1^H (400 MHz) and ^13^C (100 MHz) NMR data for **1** and **2** in CDCl_3_ (*δ* in ppm).

No.	1			2	
	δ_C_, Type	δ_H_, Mult. (J, Hz)		δ_C_, Type	δ_H_, Mult. (J, Hz)
1, 1′	NH	8.10, brs	NH	8.11, d (2.4)
2, 2′	122.8, CH	7.03, d (2.0)	121.77, CH	7.01, d (2.4)
3, 3′	112.4, C		118.67/118.40, C	
4a, 4′a	127.6, C		126.80/126.71, C	
4, 4′	118.9, CH	7.61, brd (8.0)	119.29, CH	7.04, d (7.6)
5, 5′	119.3, CH	7.12, ddd (8.0, 8.0, 0.8)	119.37, CH	7.55, dd (7.6, 7.6)
6, 6′	122.0, CH	7.21, ddd (8.0, 8.0, 0.8)	122.01/121.99, CH	7.16, dd (8.0, 7.6)
7, 7′	111.1, CH	7.34, brd (8.0)	111.24, CH	7.34, d (8.0)
7a, 7′a	136.3, C		136.60/136.56, C	
8	32.9, CH_2_	2.95, dd (14.4, 8.0) 3.09, dd (14.4, 4.8)		33.2, CH	4.64, t (7.6,)
9	71.5, CH	4.30, dddd (8.0, 8.0, 4.8, 4.8)		28.3, CH_2_	2.69, m
10	OH	2.08, brs		53.0, CH_2_	2.81, m; 2.73, m
11				S = O	
12				38.4, CH_3_	2.47, s

**Table 2:** ^1^H (600 MHz) and ^13^C NMR (150 MHz) data for **3** (*δ* in ppm).

No.	In CDCl_3_			In acetone-*d*_6_	
	δ_C_, Type	δ_H_, Mult. (J, Hz)		δ_C_, Type	δ_H_, Mult. (J, Hz)
1, 1′	NH	7.92 brs		NH	9.95, brs
2, 2′	122.0, CH	7.10, d (1.8)		123.0, CH	7.34, d (2.4)
3, 3′	123.7 C			123.7, C	
4a, 4′a	126.3, C			127.4, C	
4, 4′	121.5, CH	7.56, d (8.0)		121.9, CH	7.46, d (8.4)
5, 5′	118.5, CH	6.90, dd (8.0, 8.0)		121.3, CH	6.91, ddd (8.4, 8.4, 0.6)
6, 6′	121.2, CH	7.06, dd (8.0, 8.0)		118.6, CH	6.72, ddd (8.4, 8.4, 0.6)
7, 7′	111.0, CH	7.30, d (8.0)		112.0, CH	7.28, d (8.4)
7a, 7′a	137.1, C			138.5, C	
8	39.5, C			40.1, C	
9, 9′	36.8, CH_2_	2.55, t (6.0)		37.9, CH_2_	2.55, t (6.0)
10, 10′	23.0, CH_2_	1.66, m		23.8, CH_2_	1.68, m
11	26.8, CH_2_	1.58, m		27.6, CH_2_	1.57, m

## Supplementary Materials

HPLC analysis of the fungal metabolites in different culture media (Supplementary Figure S1), the HR-ESI-MS and NMR spectra of compounds **1**–**13** (Supplementary Figures S2–S74) are available online at http://www.mdpi.com/1660-3397/17/2/77/s1.
