# Supplementary material for: Exploration of Indole Alkaloids from Marine Fungus Pseudallescheria boydii F44-1 Using an Amino Acid-Directed Strategy
Source: Mar Drugs. 2019 Jan 23;17(2):77. doi: 10.3390/md17020077 (PMC6410255; doi:10.3390/md17020077)
Supplement: Supplementary file 1 [file marinedrugs-17-00077-s001.pdf]

Supplementary Materials

Exploration of Indole Alkaloids from Marine Fungus *Pseudallescheria boydii* F44-1 Using an Amino Acid-Directed Strategy

Mei-Xiang Yuan [1], Yi Qiu [1], Yan-Qin Ran [2], Gong-Kan Feng [3], Rong Deng [3], Xiao-Feng Zhu [3], Wen-Jian Lan [4,*] and Hou-Jin Li [1,*]

[1] School of Chemistry, Sun Yat-sen University, Guangzhou 510275, China; yuanmx3@mail2.sysu.edu.cn (M.-X.Y.); qiuyi0771@163.com (Y.Q.)
[2] School of Traditional Chinese Medicine, Guangdong Pharmaceutical University, Guangzhou 510006, China; RanyqGDPU@foxmail.com (Y.-Q.R.)
[3] State Key Laboratory of Oncology in South China, Collaborative Innovation Center for Cancer Medicine, Cancer Center, Sun Yat-sen University, Guangzhou 510060, China; fenggk@sysucc.org.cn (G.-K.F.); dengrong@sysucc.org.cn (R.D.); zhuxfeng@mail.sysu.edu.cn (X.-F.Z.)
[4] School of Pharmaceutical Sciences, Sun Yat-sen University, Guangzhou 510006, China
* Correspondence: lanwj@mail.sysu.edu.cn (W.-J.L.); ceslhj@mail.sysu.edu.cn (H.-J.L.); Tel.: +86-20-39943042 (W.-J.L.); +86-20- 84113698 (H.-J.L.)

List of Contents

Figure S1. HPLC analysis on the fungal metabolites in different culture media. S6

Figure S2. HR-(+)ESI-MS spectrum of pseudboindole A (**1**) S7

Figure S3. ^{1}H NMR spectrum of pseudboindole A (**1**) in $CDCl_3$ (400 MHz) S8

Figure S4. ^{13}C NMR spectrum of pseudboindole A (**1**) in $CDCl_3$ (100 MHz) S9

Figure S5. DEPT 135 spectrum of pseudboindole A (**1**) in $CDCl_3$ (100 MHz) S10

Figure S6. DEPT 90 spectrum of pseudboindole A (**1**) in $CDCl_3$ (100 MHz) S11

Figure S7. HMQC spectrum of pseudboindole A (**1**) in $CDCl_3$ S12

Figure S8. ^{1}H–^{1}H COSY spectrum of pseudboindole A (**1**) in $CDCl_3$ S13
Figure S9. HMBC spectrum of pseudboindole A (**1**) in $CDCl_3$ S14
Figure S10. NOESY spectrum of pseudboindole A (**1**) in $CDCl_3$ S15
Figure S11. HR-(-)ESI-MS spectrum of pseudboindole B (**2**) S16
Figure S12. ^{1}H NMR spectrum of pseudboindole B (**2**) in $CDCl_3$ (400 MHz) S17
Figure S13. ^{13}C NMR spectrum of pseudboindole B (**2**) in $CDCl_3$ (100 MHz) S18
Figure S14. DEPT 135 spectrum of pseudboindole B (**2**) in $CDCl_3$ (100 MHz) S19
Figure S15. DEPT 90 spectrum of pseudboindole B (**2**) in $CDCl_3$ (100 MHz) S20
Figure S16. HMQC spectrum of pseudboindole B (**2**) in $CDCl_3$ S21
Figure S17. ^{1}H–^{1}H COSY spectrum of pseudboindole B (**2**) in $CDCl_3$ S22
Figure S18. HMBC spectrum of pseudboindole B (**2**) in $CDCl_3$ S23
Figure S19. NOESY spectrum of pseudboindole B (**2**) in $CDCl_3$ S24
Figure S20. HR-(-)ESI-MS spectrum of 3,3'-cyclohexylidenebis(1*H*-indole) (**3**) S25
Figure S21. ^{1}H NMR spectrum of 3,3'-cyclohexylidenebis(1*H*-indole) (**3**) in $CDCl_3$ (600 MHz) S26
Figure S22. ^{13}C NMR spectrum of 3,3'-cyclohexylidenebis(1*H*-indole) (**3**) in $CDCl_3$ (150 MHz) S27
Figure S23. DEPT 135 spectrum of 3,3'-cyclohexylidenebis(1*H*-indole) (**3**) in $CDCl_3$ (150 MHz) S28
Figure S24. DEPT 90 spectrum of 3,3'-cyclohexylidenebis(1*H*-indole) (**3**) in $CDCl_3$ (150 MHz) S29
Figure S25. HMQC spectrum of 3,3'-cyclohexylidenebis(1*H*-indole) (**3**) in $CDCl_3$ S30
Figure S26. ^{1}H–^{1}H COSY spectrum of 3,3'-cyclohexylidenebis(1*H*-indole) (**3**) in $CDCl_3$ S31

Figure S27. HMBC spectrum of 3,3'-cyclohexylidenebis(1*H*-indole) (**3**) in $CDCl_3$ S32
Figure S28. NOESY spectrum of 3,3'-cyclohexylidenebis(1*H*-indole) (**3**) in $CDCl_3$.......... S33
Figure S29. ^{1}H NMR spectrum of 3,3'-cyclohexylidenebis(1*H*-indole) (**3**) in acetone-d_6 (600 MHz).......... S34
Figure S30. ^{13}C NMR spectrum of 3,3'-cyclohexylidenebis(1*H*-indole) (**3**) in acetone-d_6 (150 MHz).......... S35
Figure S31. DEPT 135 spectrum of 3,3'-cyclohexylidenebis(1*H*-indole) (**3**) in acetone-d_6 (150 MHz) S36
Figure S32. DEPT 90 spectrum of 3,3'-cyclohexylidenebis(1*H*-indole) (**3**) in acetone-d_6 (150 MHz) S37
Figure S33. HMQC spectrum of 3,3'-cyclohexylidenebis(1*H*-indole) (**3**) in acetone-d_6.......... S38
Figure S34. HR-(+)ESI-MS spectrum of 3,3- bis(3-indolyl) butan-2-one (**4**) S39
Figure S35. ^{1}H NMR spectrum of 3,3- bis(3-indolyl) butan-2-one (**4**) in $CDCl_3$ (400 MHz) S40
Figure S36. ^{13}C NMR spectrum of3,3- bis(3-indolyl) butan-2-one (**4**) in $CDCl_3$ (100 MHz) S41
Figure S37. HR-(+)ESI-MS spectrum of 2-[2,2-di(1*H*-indol-3-yl) ethyl] aniline (**5**).......... S42
Figure S38. ^{1}H NMR spectrum of 2-[2,2-di(1*H*-indol-3-yl) ethyl] aniline (**5**) in $CDCl_3$ (600 MHz).......... S43
Figure S39. ^{13}C NMR spectrum of 2-[2,2-di(1*H*-indol-3-yl) ethyl] aniline (**5**) in $CDCl_3$ (150 MHz).......... S44
Figure S40. DEPT 135 spectrum of 2-[2,2-di(1*H*-indol-3-yl) ethyl] aniline (**5**) in $CDCl_3$ (150 MHz) S45
Figure S41. HMQC spectrum of 2-[2,2-di(1*H*-indol-3-yl) ethyl] aniline (**5**) in $CDCl_3$.......... S46
Figure S42. ^{1}H–^{1}H COSY spectrum of 2-[2,2-di(1*H*-indol-3-yl) ethyl] aniline (**5**) in $CDCl_3$ S47
Figure S43. HMBC spectrum of 2-[2,2-di(1*H*-indol-3-yl) ethyl] aniline (**5**) in $CDCl_3$.......... S48
Figure S44. NOESY spectrum of2-[2,2-di(1*H*-indol-3-yl) ethyl] aniline (**5**) in $CDCl_3$ S49
Figure S45. ^{1}H NMR spectrum of 3,3'-Diindolyl(phenyl)methane (**6**) in $CDCl_3$ (400 MHz).......... S50

Figure S46. ^{13}C NMR spectrum of 3,3'-Diindolyl(phenyl)methane (**6**) in $CDCl_3$ (100 MHz)....S51
Figure S47. ^{1}H NMR spectrum of 1,1-(3,3'-Diindolyl)-2-phenylethane (**7**) in acetone-d_6 (400 MHz)....S52
Figure S48. ^{13}C NMR spectrum of 1,1-(3,3'-Diindolyl)-2-phenylethane (**7**) in acetone-d_6 (100 MHz)....S53
Figure S49. ^{1}H NMR spectrum of perlolyrin (**8**) in acetone-d_6 (400 MHz)....S54
Figure S50. ^{13}C NMR spectrum of perlolyrin (**8**) in acetone-d_6 (100 MHz)....S55
Figure S51. ^{1}H NMR spectrum of pityriacitrin (**9**) in acetone-d_6 (600 MHz)....S56
Figure S52. ^{13}C NMR spectrum of pityriacitrin (**9**) in A acetone-d_6 (150 MHz)....S57
Figure S53. DEPT 135 spectrum of pityriacitrin (**9**) in acetone-d_6 (150 MHz)....S58
Figure S54. DEPT 90 spectrum of pityriacitrin (**9**) in acetone-d_6 (150 MHz)....S59
Figure S55. HMQC spectrum of pityriacitrin (**9**) in acetone-d_6....S60
Figure S56. ^{1}H–^{1}H COSY spectrum of pityriacitrin (**9**) in acetone-d_6....S61
Figure S57. HMBC spectrum of pityriacitrin (**9**) in acetone-d_6....S62
Figure S58. NOESY spectrum of pityriacitrin (**9**) in acetone-d_6....S63
Figure S59. ^{1}H NMR spectrum of 1-acetyl-β-carboline (**10**) in $CDCl_3$ (400 MHz)....S64
Figure S60. ^{13}C NMR spectrum of 1-acetyl-β-carboline (**10**) in $CDCl_3$ (100 MHz)....S65
Figure S61. HMQC spectrum of 1-acetyl-β-carboline (**10**) in $CDCl_3$....S66
Figure S62. ^{1}H–^{1}H COSY spectrum of 1-acetyl-β-carboline (**10**) in $CDCl_3$....S67
Figure S63. HMBC spectrum of 1-acetyl-β-carboline (**10**) in $CDCl_3$....S68
Figure S64. ^{1}H NMR spectrum of 3-hydroxy-β-carboline (**11**) in acetone-d_6 (400 MHz)....S69

Figure S65. ^{13}C NMR spectrum of 3-hydroxy-β-carboline (**11**) in acetone-d_6 (100 MHz) S70

Figure S66. ^{1}H NMR spectrum of 1-(9*H*-pyrido [3,4-b] indol-1-yl) ethan-1-ol (**12**) in methanol-d_4 (400 MHz) S71

Figure S67. ^{13}C NMR spectrum of 1-(9*H*-pyrido [3,4-b] indol-1-yl) ethan-1-ol (**12**) in methanol-d_4 (100 MHz) S72

Figure S68. DEPT 135 spectrum of 1-(9*H*-pyrido [3,4-b] indol-1-yl) ethan-1-ol (**12**) in methanol-d_4 (100 MHz) S73

Figure S69. DEPT 90 spectrum of 1-(9*H*-pyrido [3,4-b] indol-1-yl) ethan-1-ol (**12**) in methanol-d_4 (100 MHz) S74

Figure S70. HMQC spectrum of 1-(9*H*-pyrido [3,4-b] indol-1-yl) ethan-1-ol (**12**) in methanol-d_4 .... S75

Figure S71. ^{1}H–^{1}H COSY spectrum of 1-(9*H*-pyrido [3,4-b] indol-1-yl) ethan-1-ol (**12**) in methanol-d_4 .... S76

Figure S72. HMBC spectrum of 1-(9*H*-pyrido [3,4-b] indol-1-yl) ethan-1-ol (**12**) in methanol-d_4 .... S77

Figure S73. ^{1}H NMR spectrum of N_b-acetyltryptamine (**13**) in acetone-d_6 (400 MHz) S78

Figure S74. ^{13}C NMR spectrum of N_b-acetyltryptamine (**13**) in acetone-d_6 (100 MHz) S79

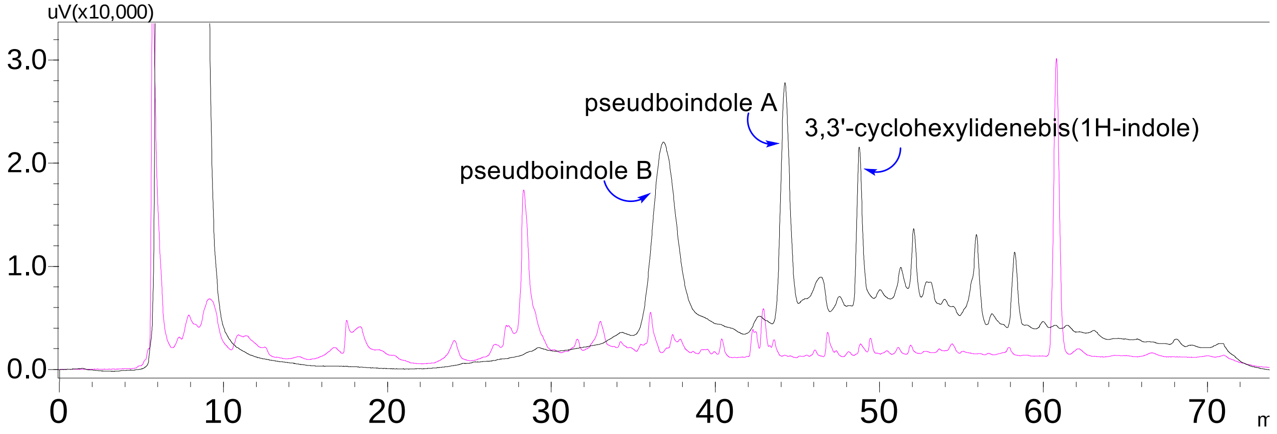

Figure S1. HPLC analysis on the fungal metabolites in different culture media.

HPLC profiles detected at 254 nm of the metabolite extracts of marine fungus *Pseudallescheria boydii* F44-1 cultured in GPY medium (pink line) and GPY medium supplemented with various amino acids (black line). HPLC analysis was used a Shimadzu LC-20AT HPLC pump (Shimadzu Corporation, Nakagyu-ku, Kyoto, Japan) equipped with an SPD-20A dual λ absorbance detector (Shimadzu Corporation, Nakagyu-ku, Kyoto, Japan) and a Shiseido spolar C18 column (4.6 mm I.D. × 250 mm, 5 μm) and a step gradient elution with $CH_3CN–H_2O$. LC time program was listed below:

Time/min	Module	Action	Value/%
0.01	Pumps	H_2O Conc.	30
10.00	Pumps	H_2O Conc.	30
40.00	Pumps	H_2O Conc.	100
60.00	Pumps	H_2O Conc.	100
65.00	Pumps	H_2O Conc.	30
70.00	Pumps	H_2O Conc.	30
75.00	Controller	Stop	

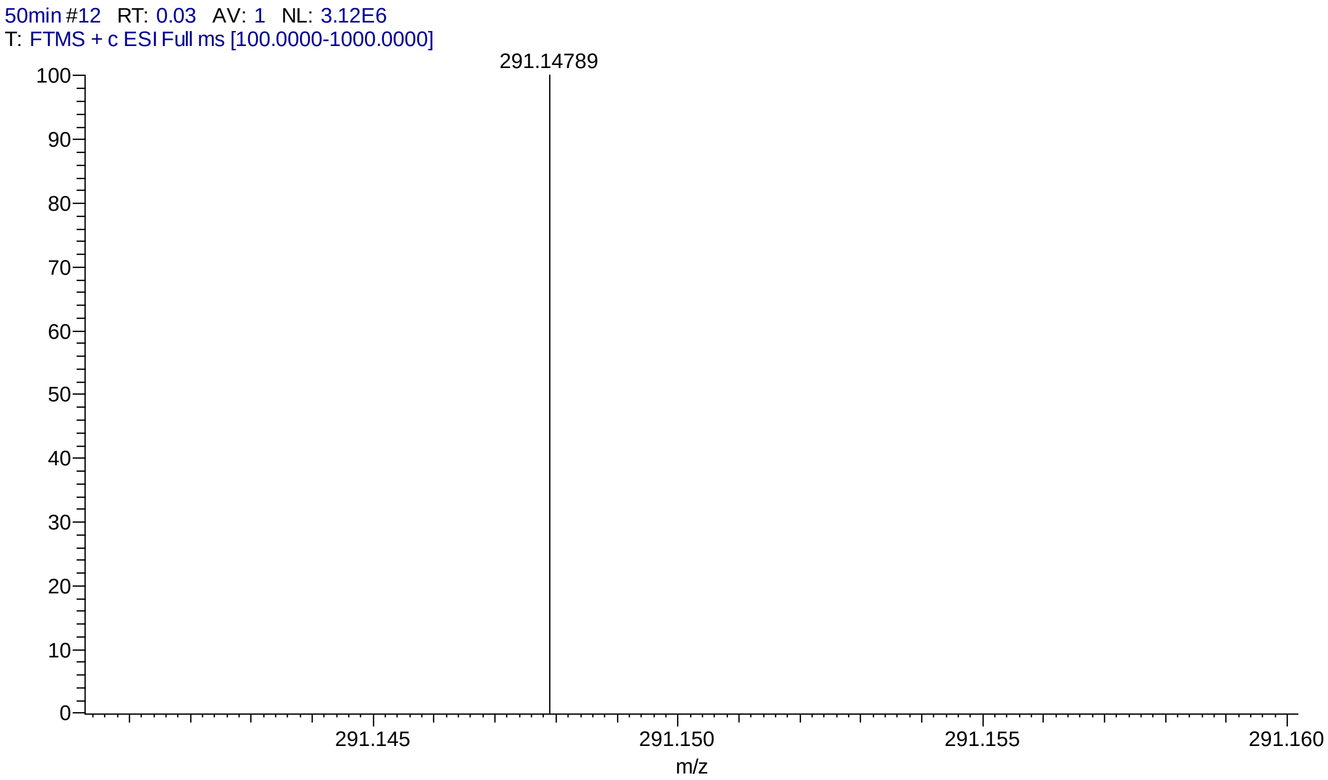

SPECTRUM - simulation:

m/z	Theo. Mass	Delta (ppm)	RDB equiv.	Composition
291.14789	291.14919	-4.46	11.5	C19 H19 O N2

Figure S2. HR-(+)ESI-MS spectrum of pseudboindole A (**1**).

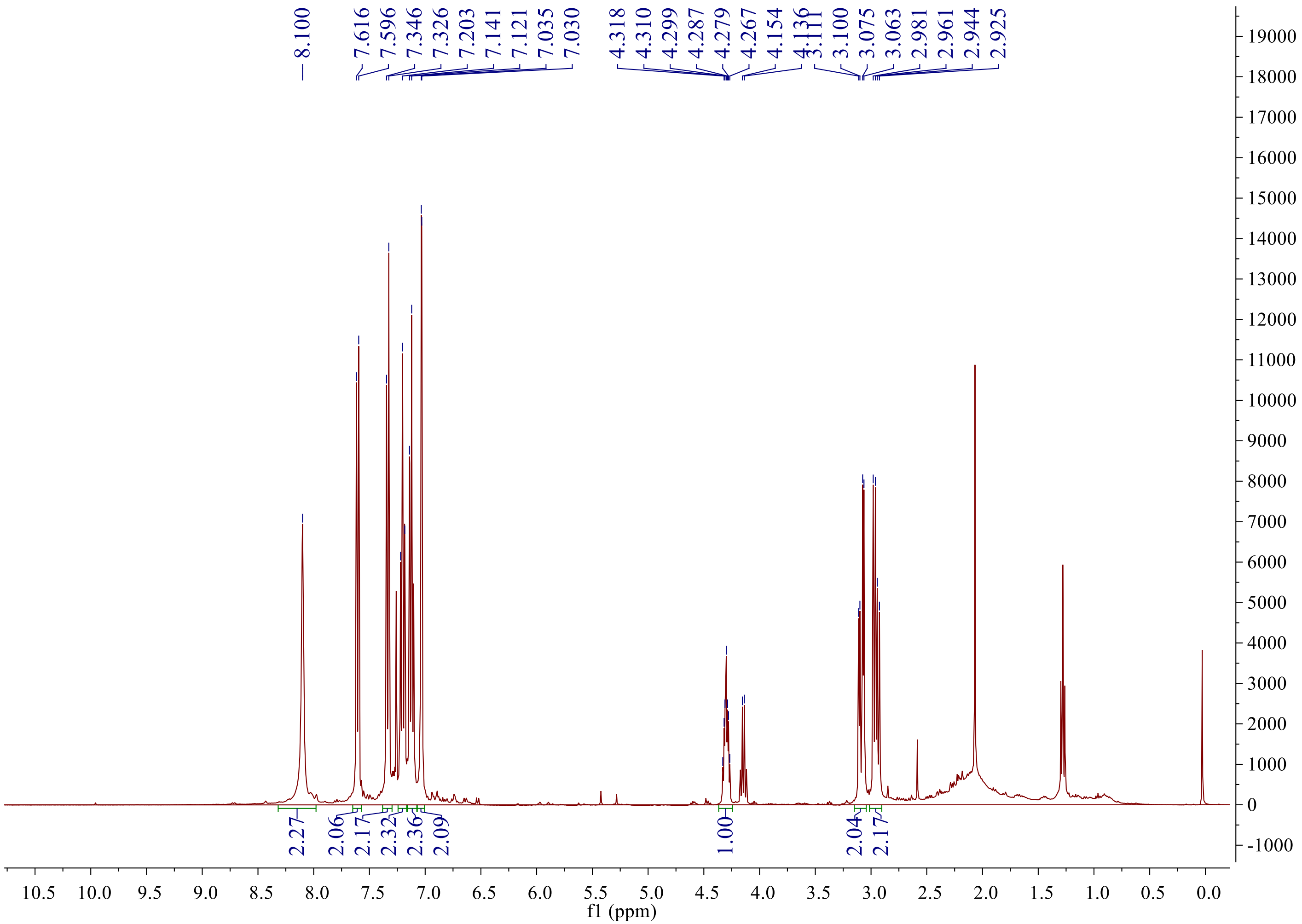

Figure S3. ^{1}H NMR spectrum of pseudboindole A (**1**) in $CDCl_3$ (400 MHz).

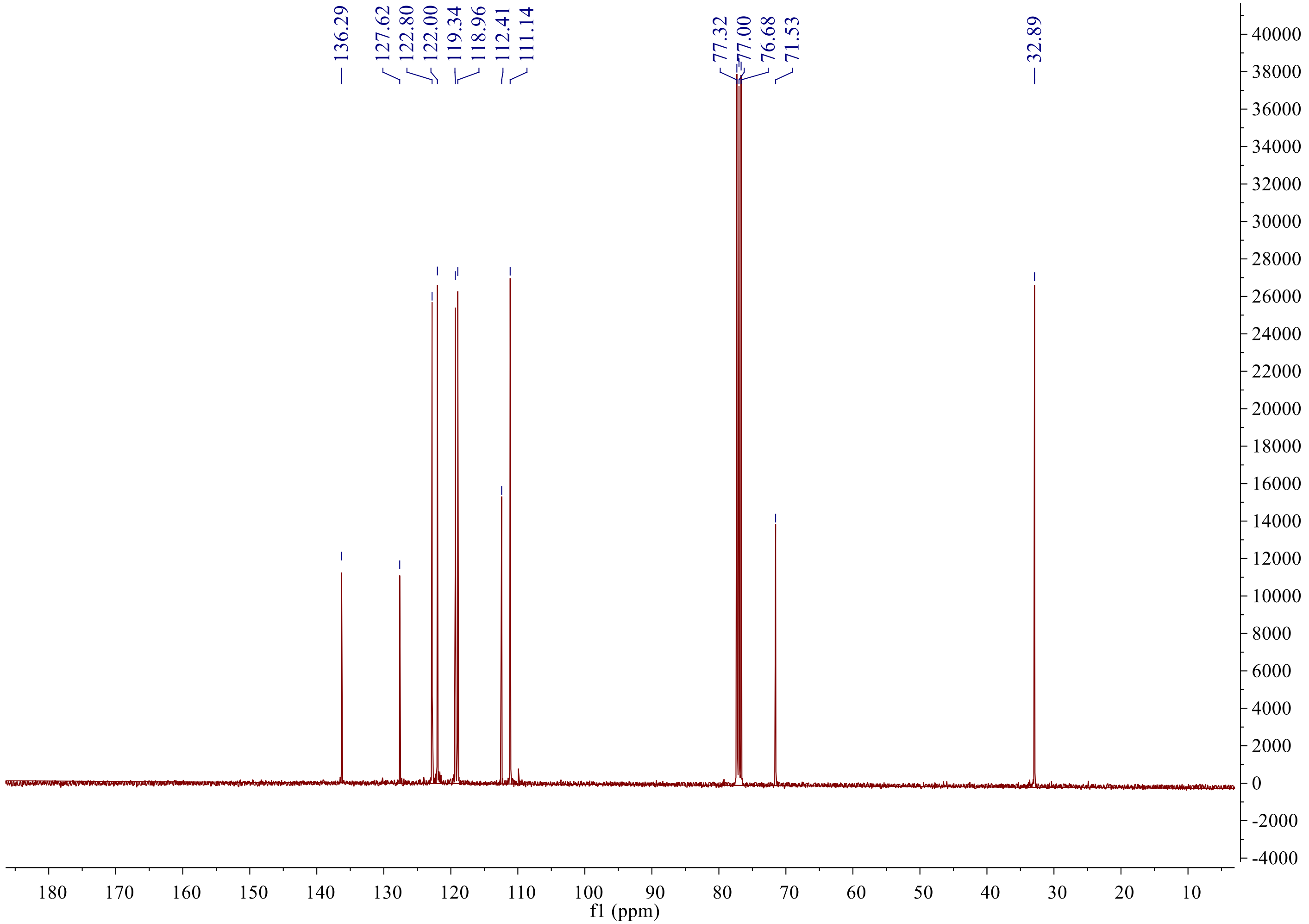

Figure S4. ^{13}C NMR spectrum of pseudboindole A (**1**) in $CDCl_3$ (100 MHz).

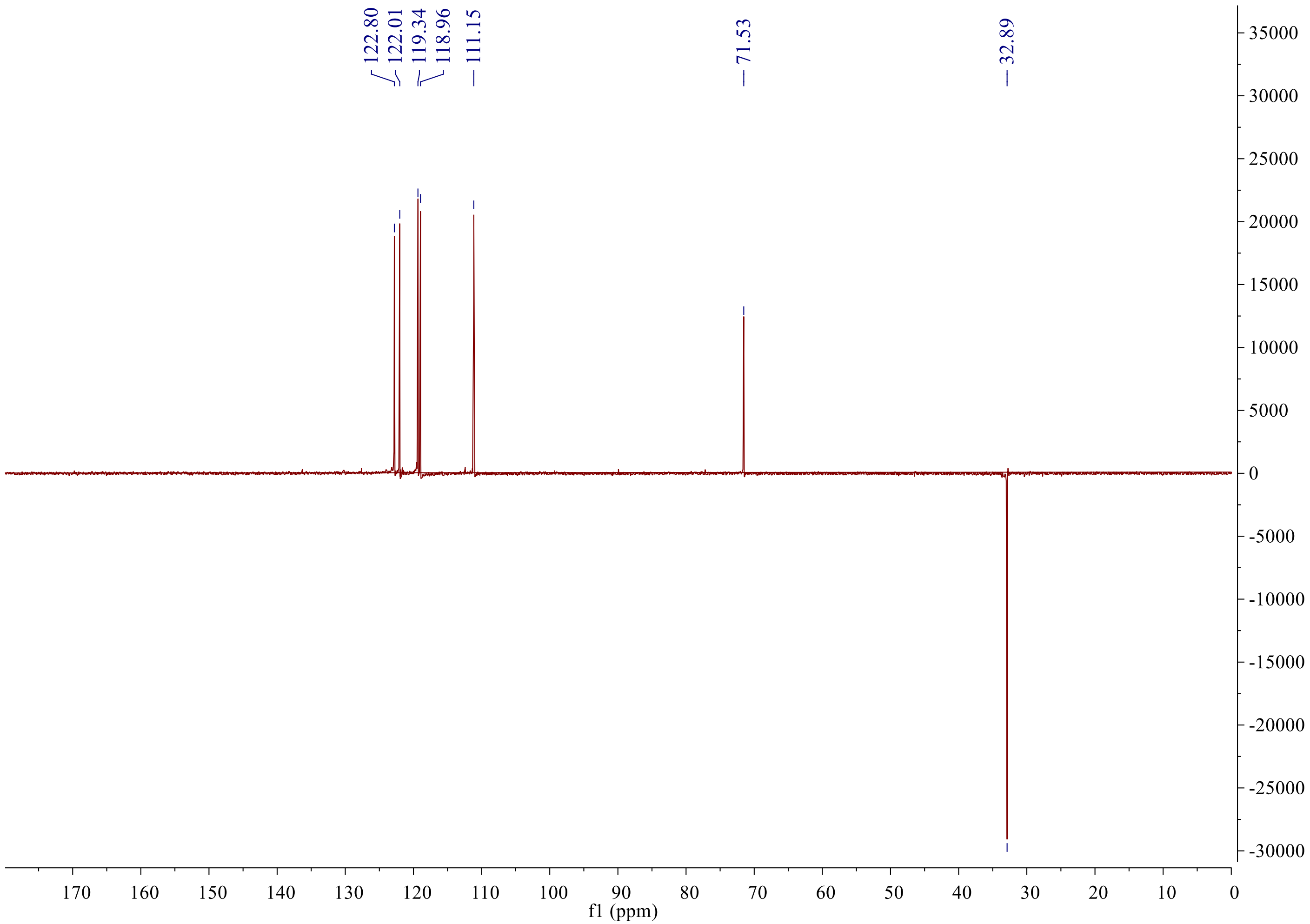

Figure S5. DEPT 135 spectrum of pseudboindole A (**1**) in $CDCl_3$ (100 MHz).

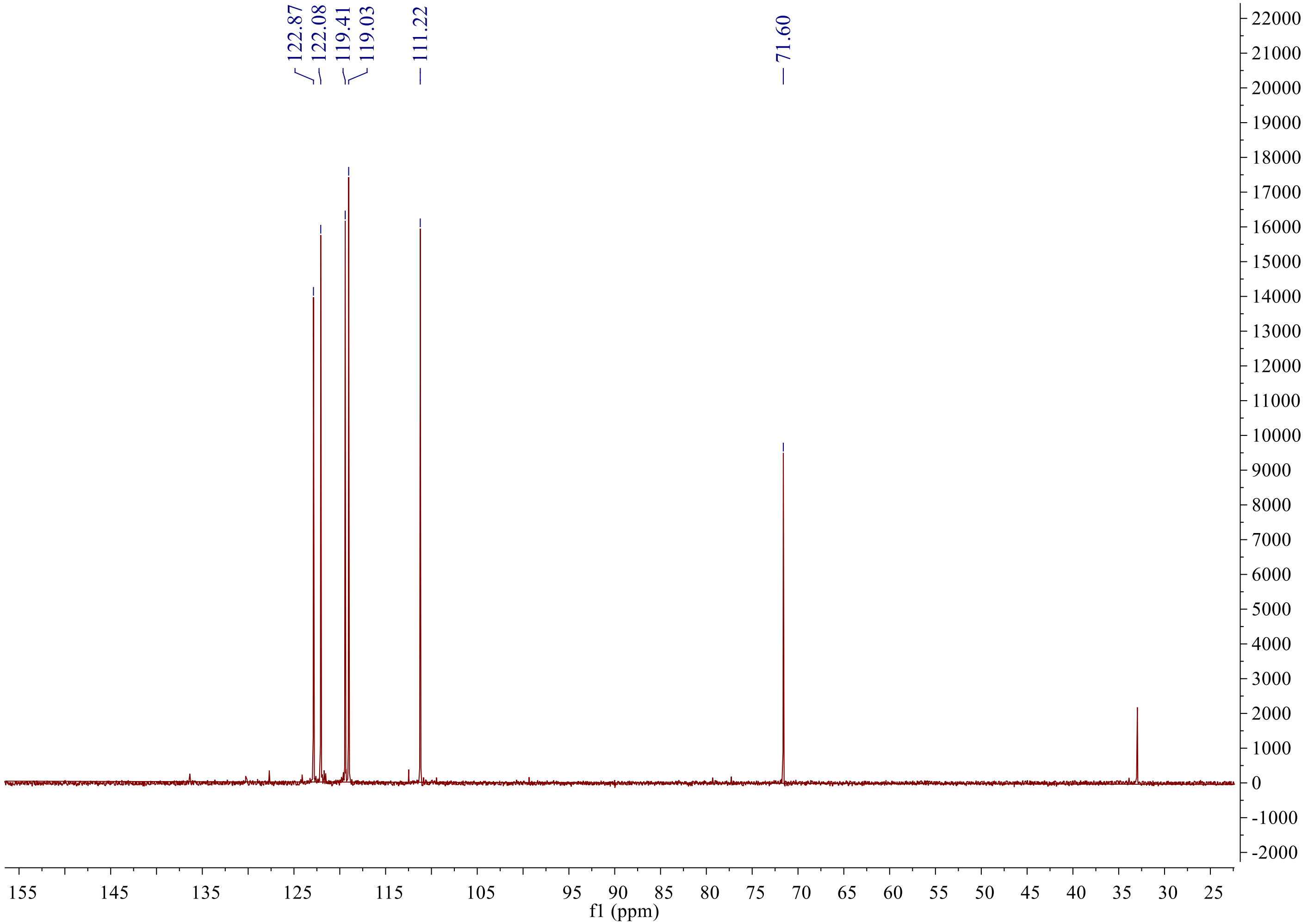

Figure S6. DEPT 90 spectrum of pseudboindole A (**1**) in $CDCl_3$ (100 MHz).

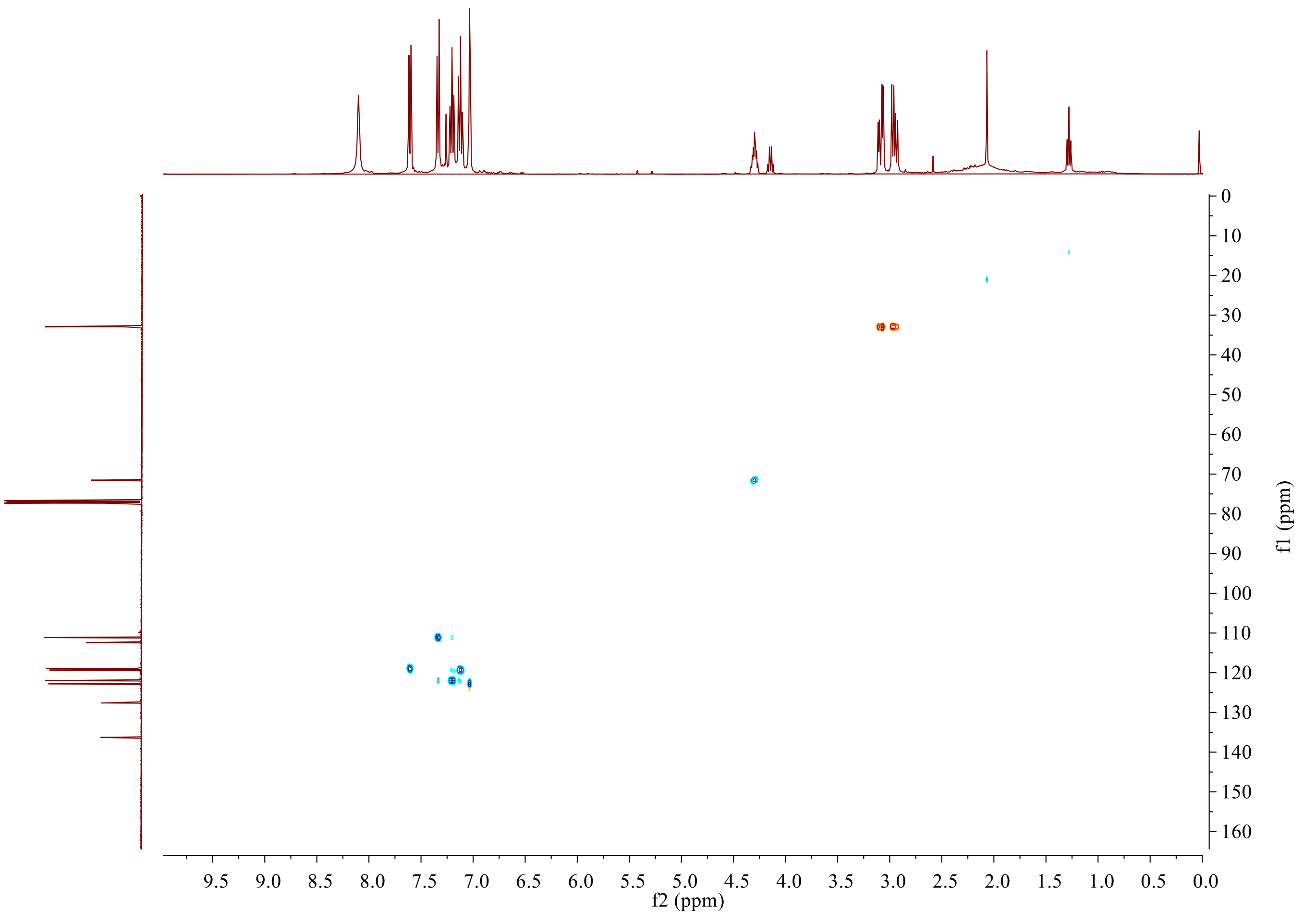

Figure S7. HMQC spectrum of pseudboindole A (**1**) in $CDCl_3$.

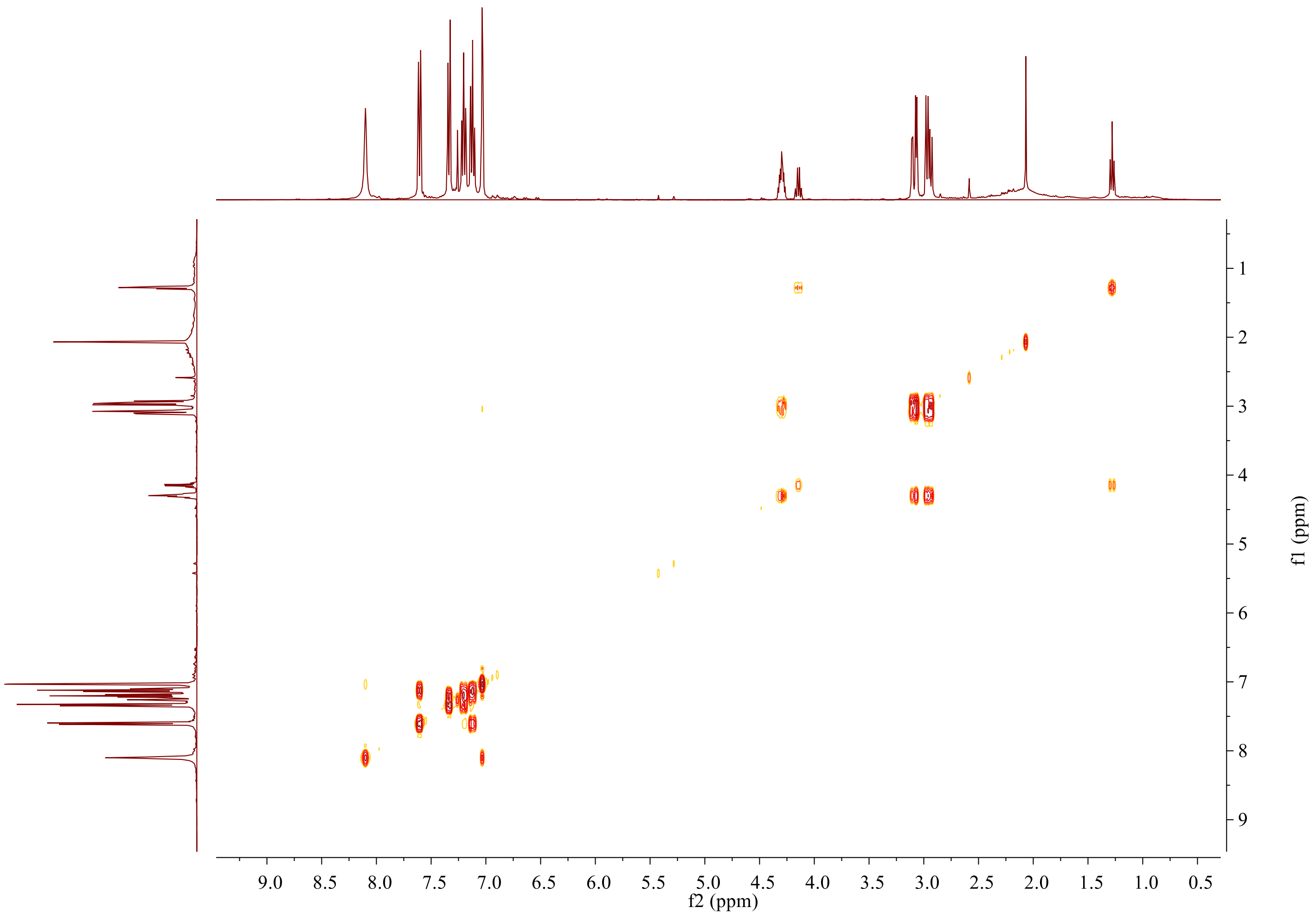

Figure S8. ^1H–^1H COSY spectrum of pseudboindole A (**1**) in $CDCl_3$.

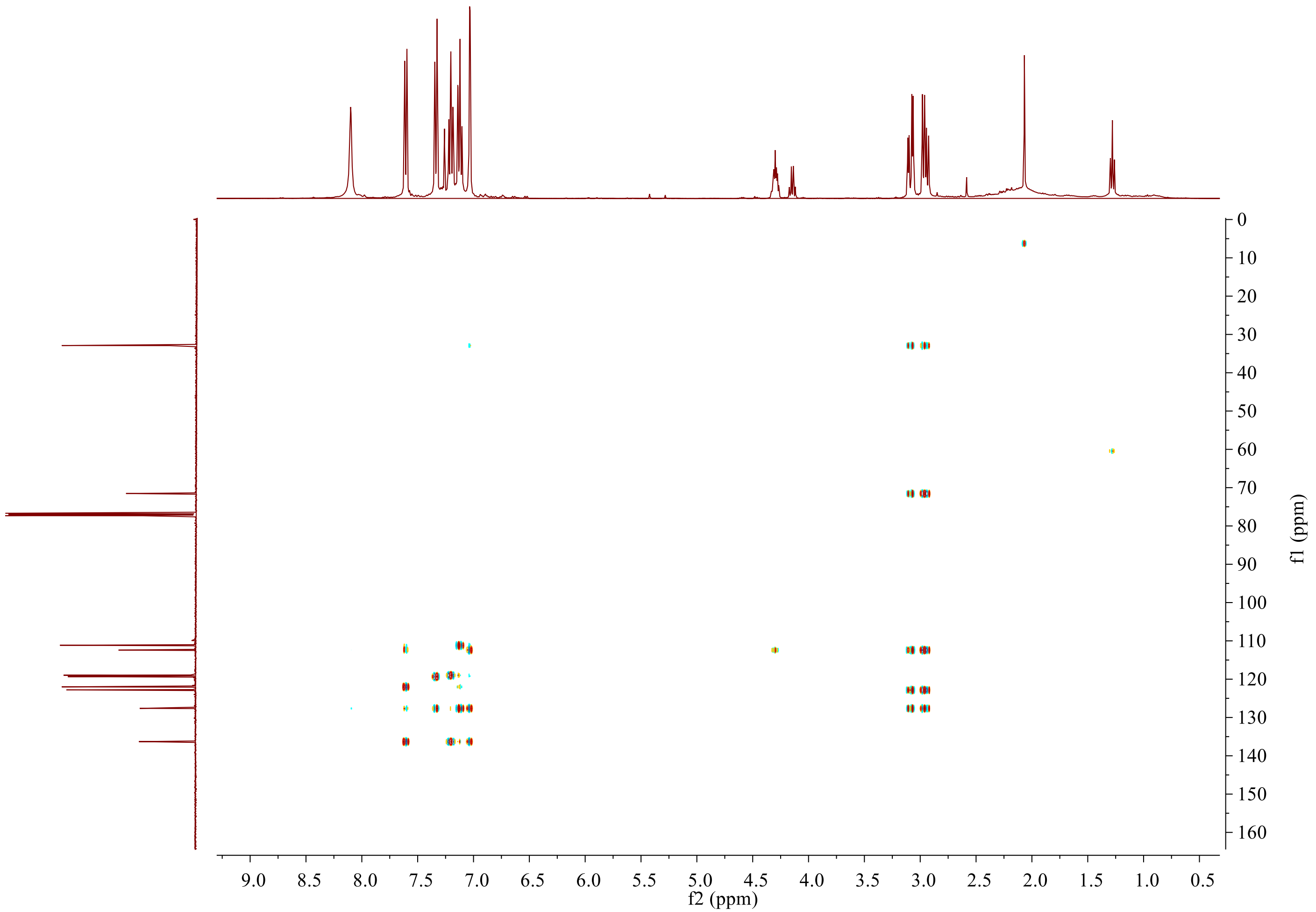

Figure S9. HMBC spectrum of pseudboindole A (**1**) in $CDCl_3$.

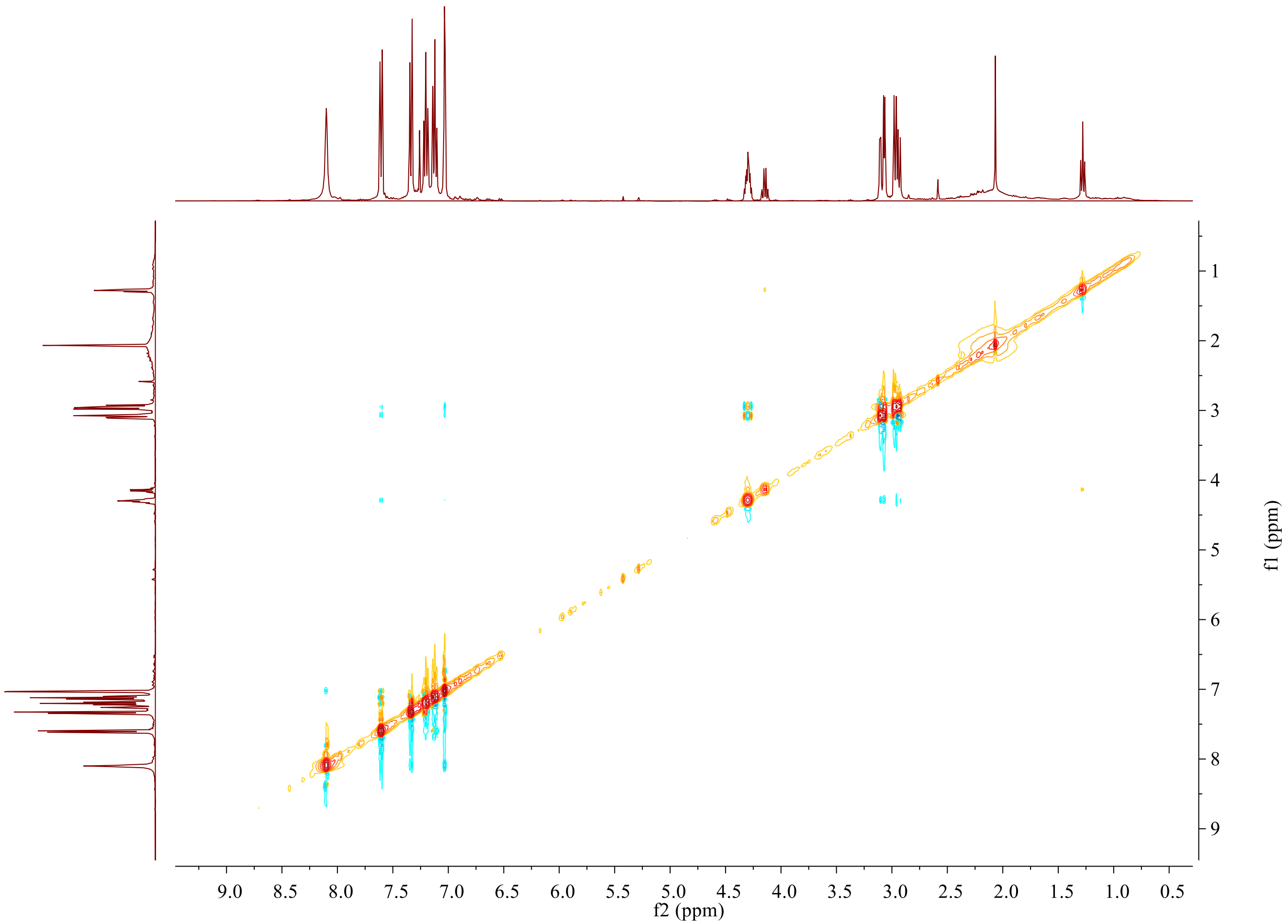

Figure S10. NOESY spectrum of pseudboindole A (**1**) in $CDCl_3$.

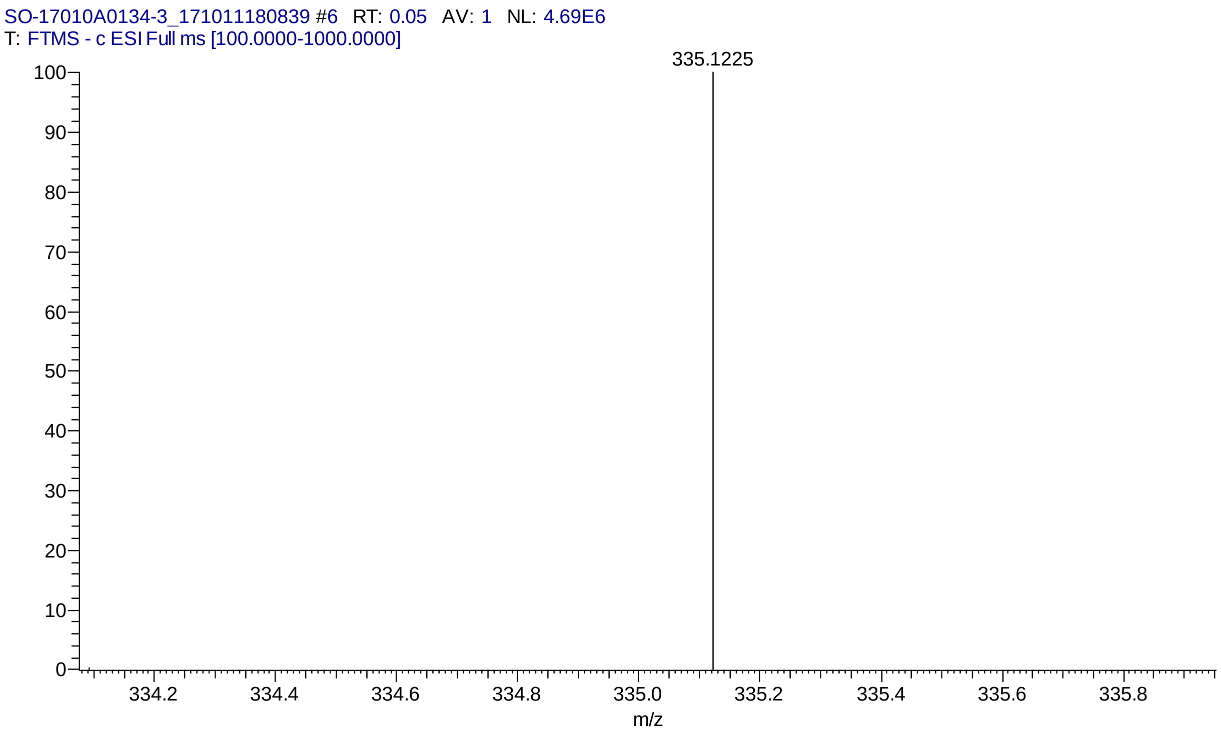

SPECTRUM - simulation:

m/z	Theo. Mass	Delta (ppm)	RDB equiv.	Composition
335.1225	335.1224	0.52	12.5	C20 H19 O N2 S

Figure S11. HR-(-)ESI-MS spectrum of pseudboindole B (**2**).

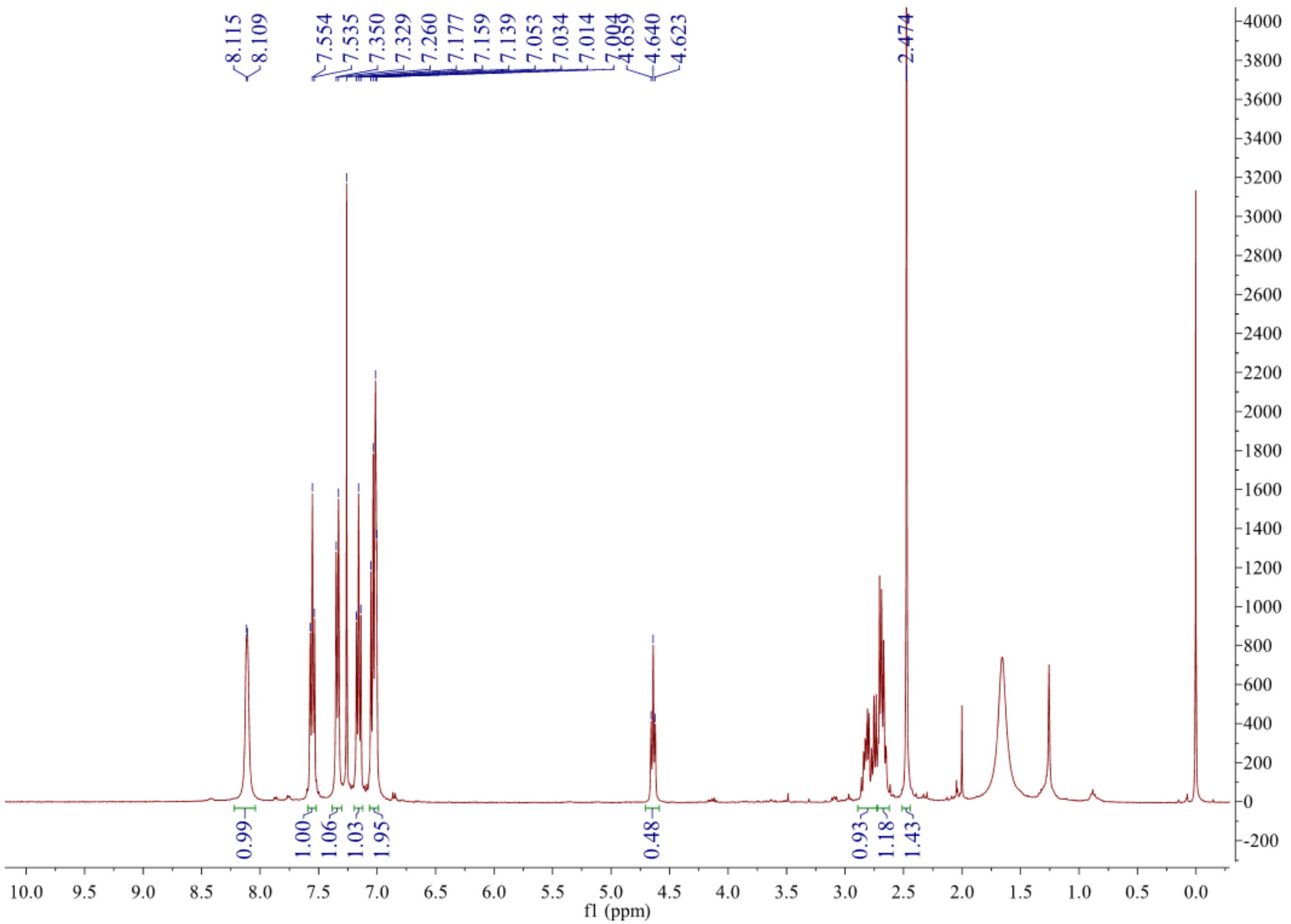

Figure S12. ^{1}H NMR spectrum of pseudboindole B (**2**) in $CDCl_3$ (400 MHz).

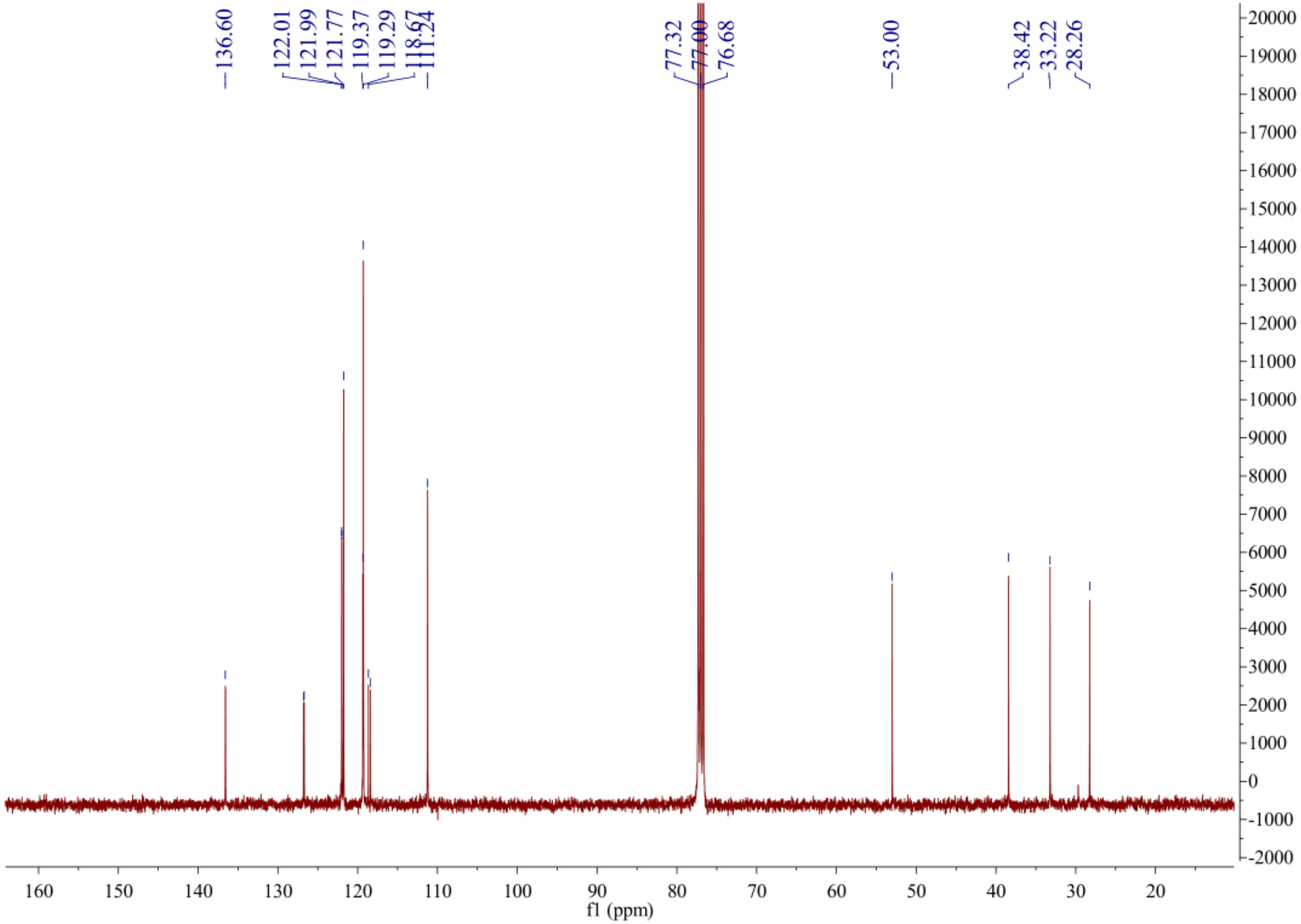

Figure S13. ^{13}C NMR spectrum of pseudboindole B (**2**) in $CDCl_3$ (100 MHz).

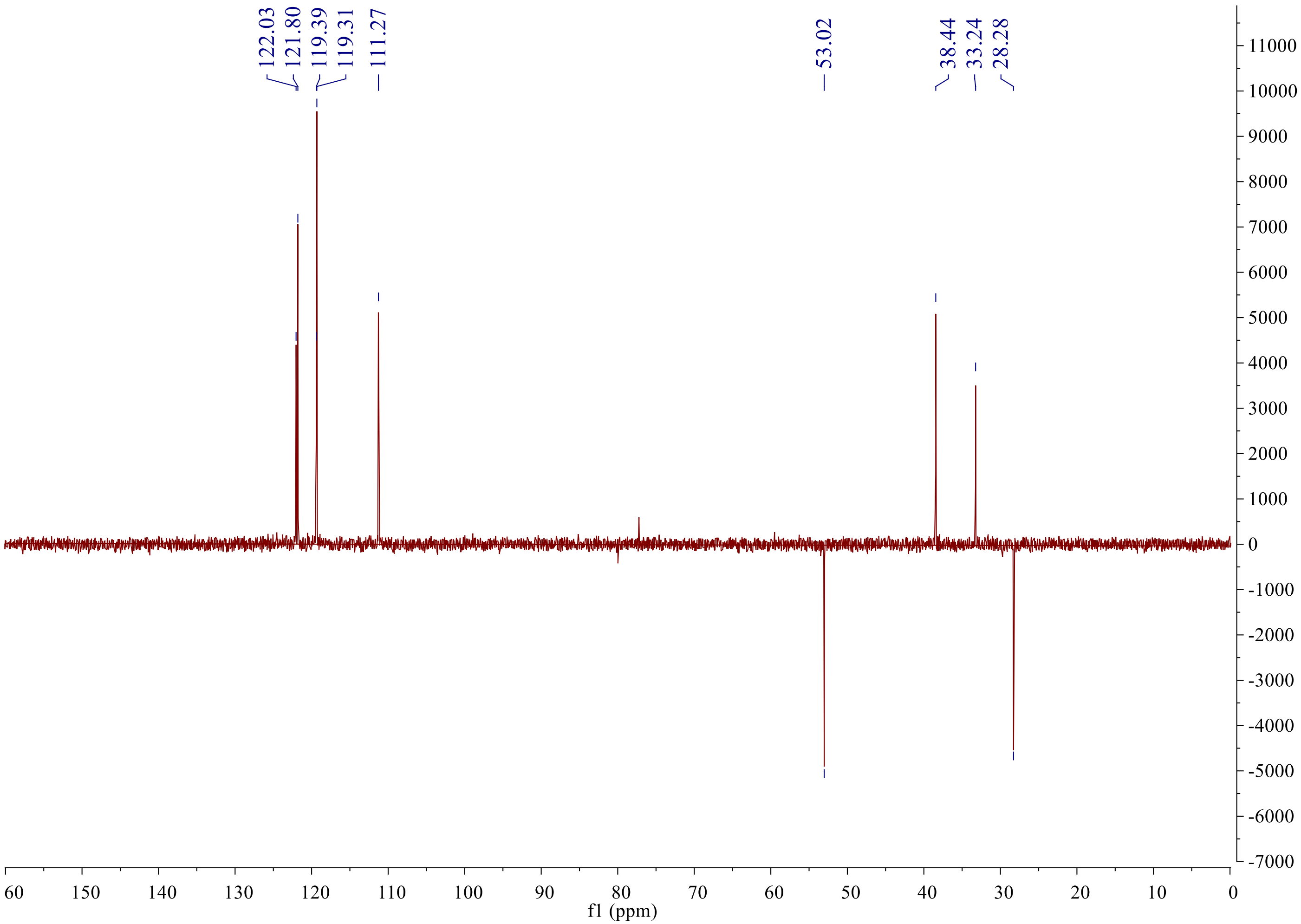

Figure S14. DEPT 135 spectrum of pseudboindole B (**2**) in $CDCl_3$ (100 MHz).

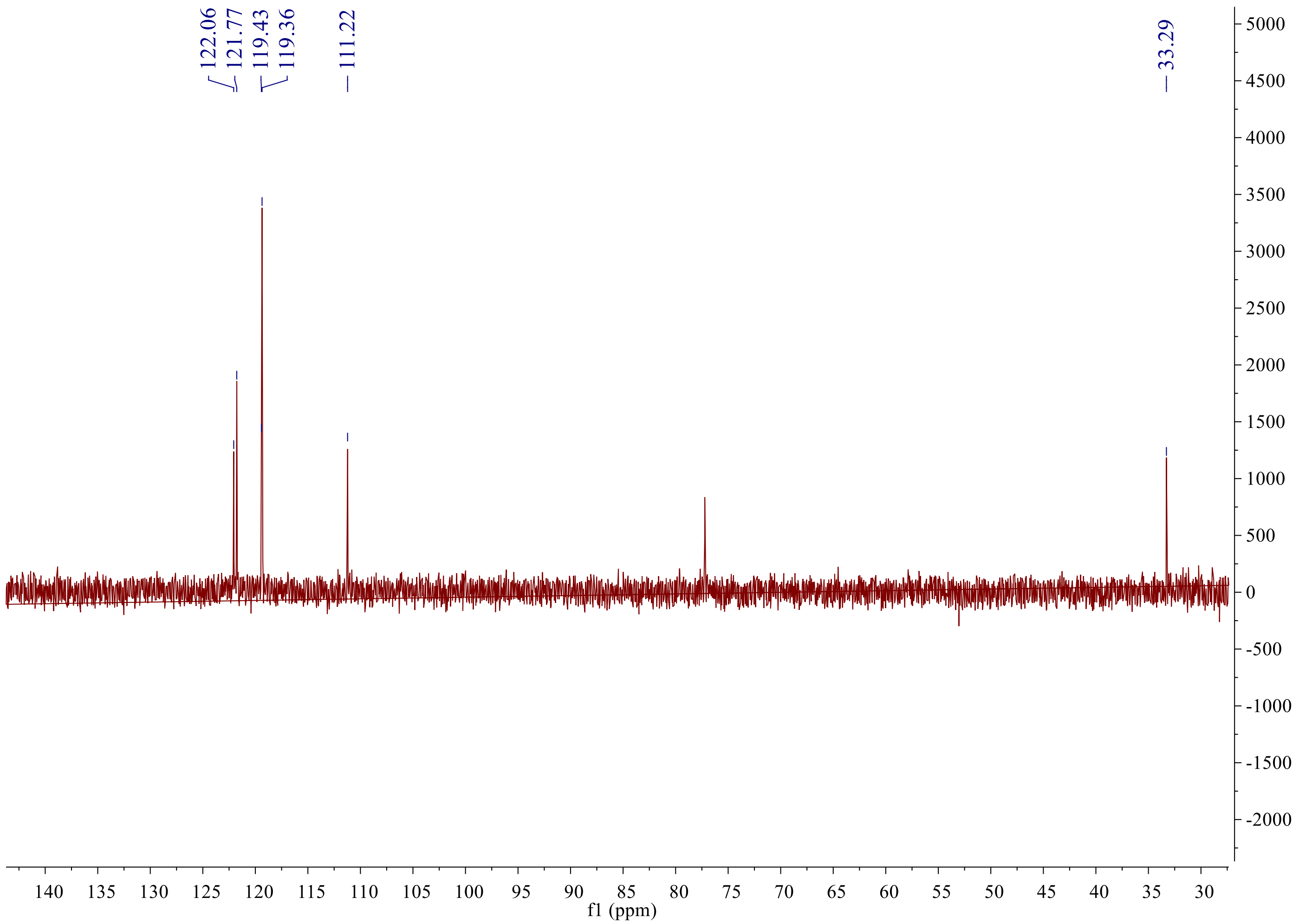

Figure S15. DEPT 90 spectrum of pseudboindole B (**2**) in $CDCl_3$ (100 MHz).

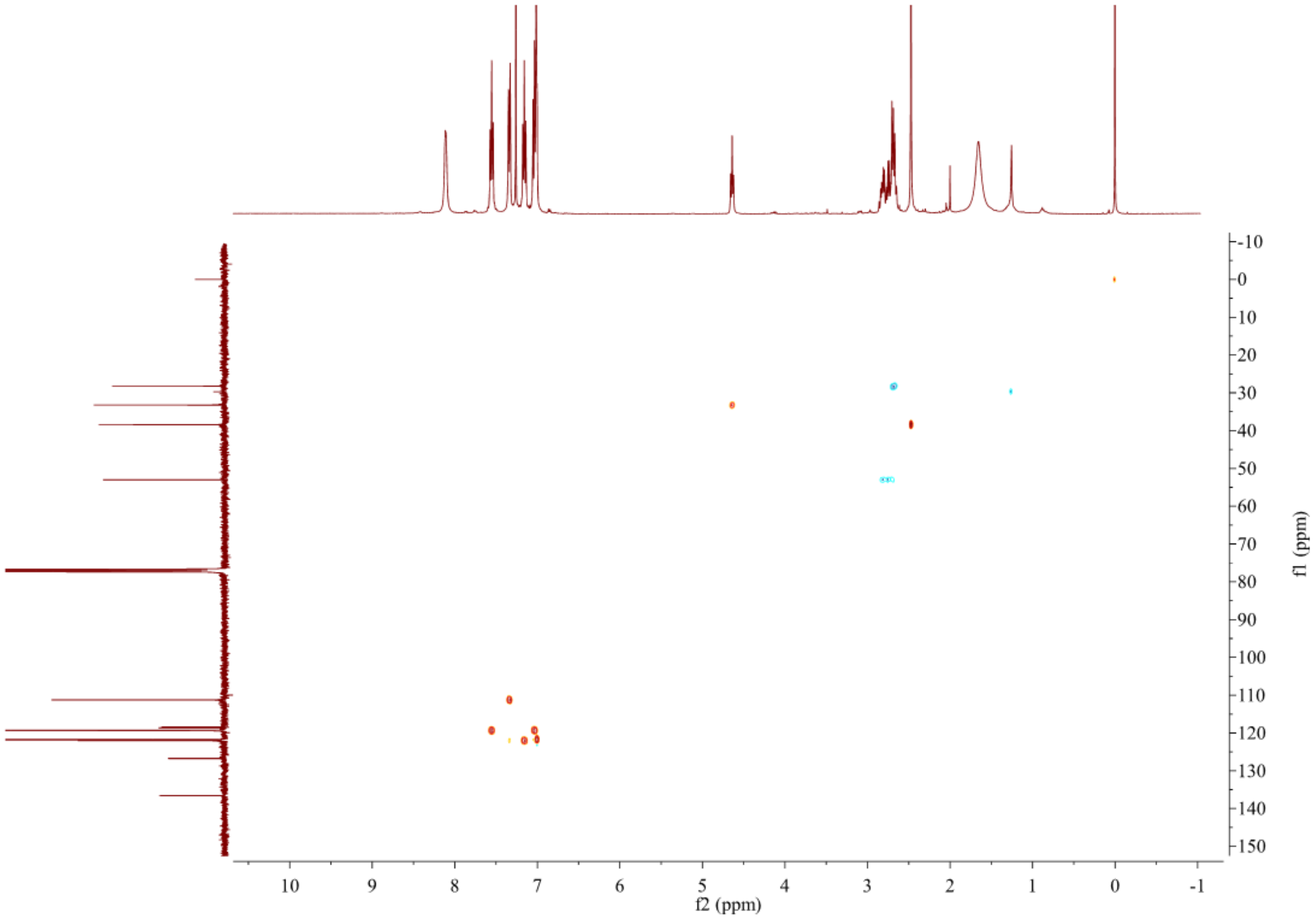

Figure S16. HMQC spectrum of pseudboindole B (**2**) in $CDCl_3$.

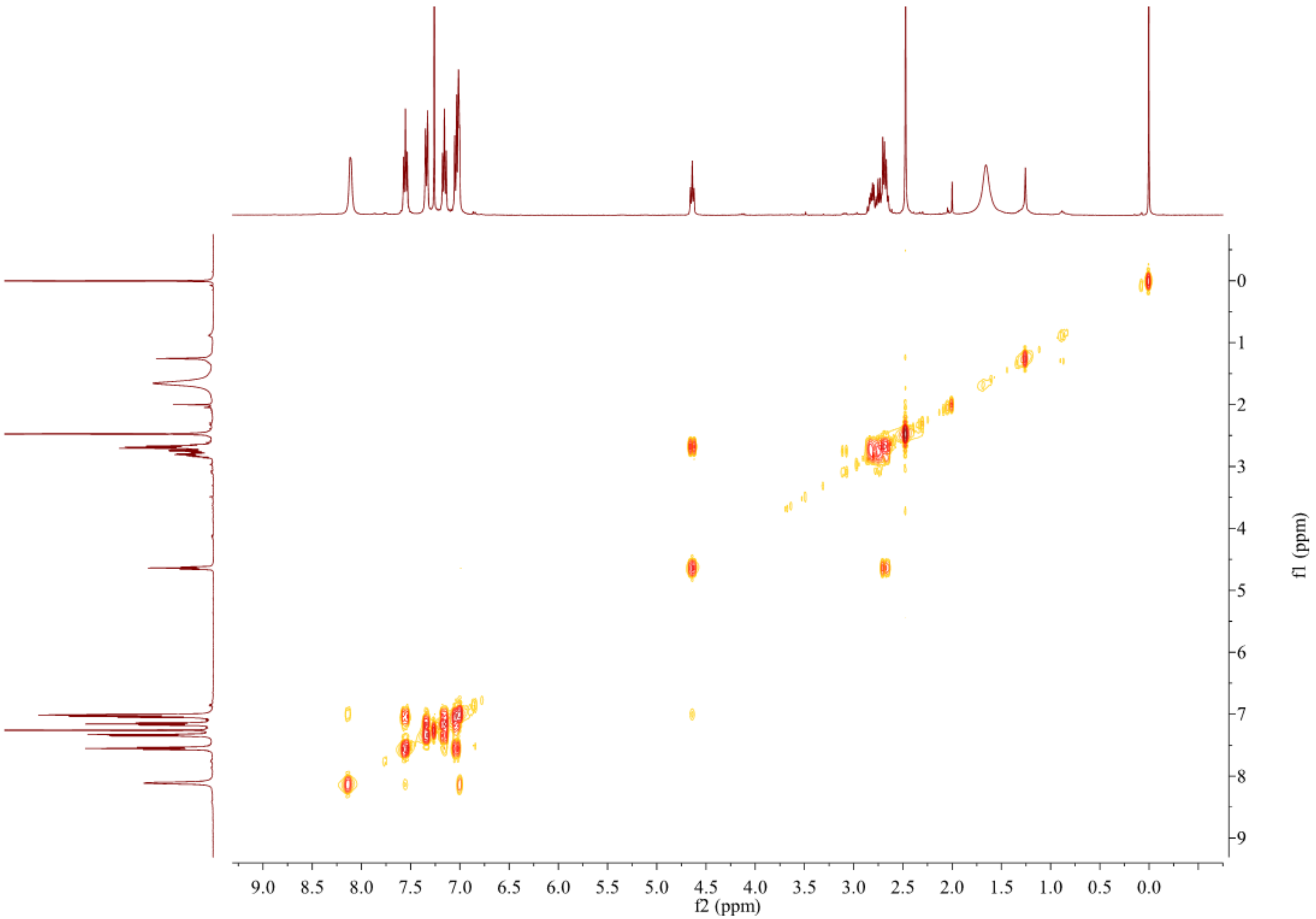

Figure S17. ^{1}H–^{1}H COSY spectrum of pseudboindole B (**2**) in $CDCl_3$.

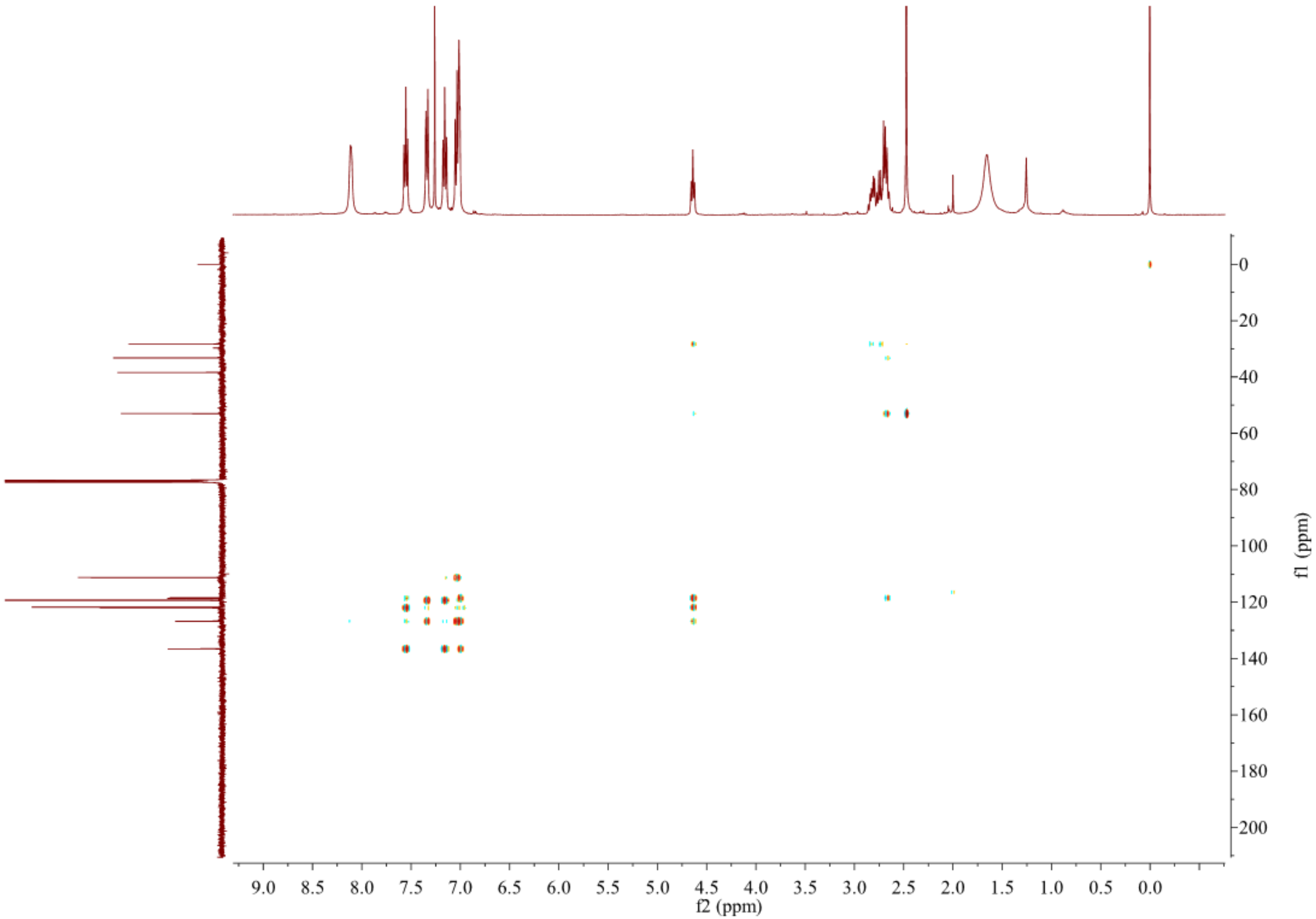

Figure S18. HMBC spectrum of pseudboindole B (**2**) in $CDCl_3$.

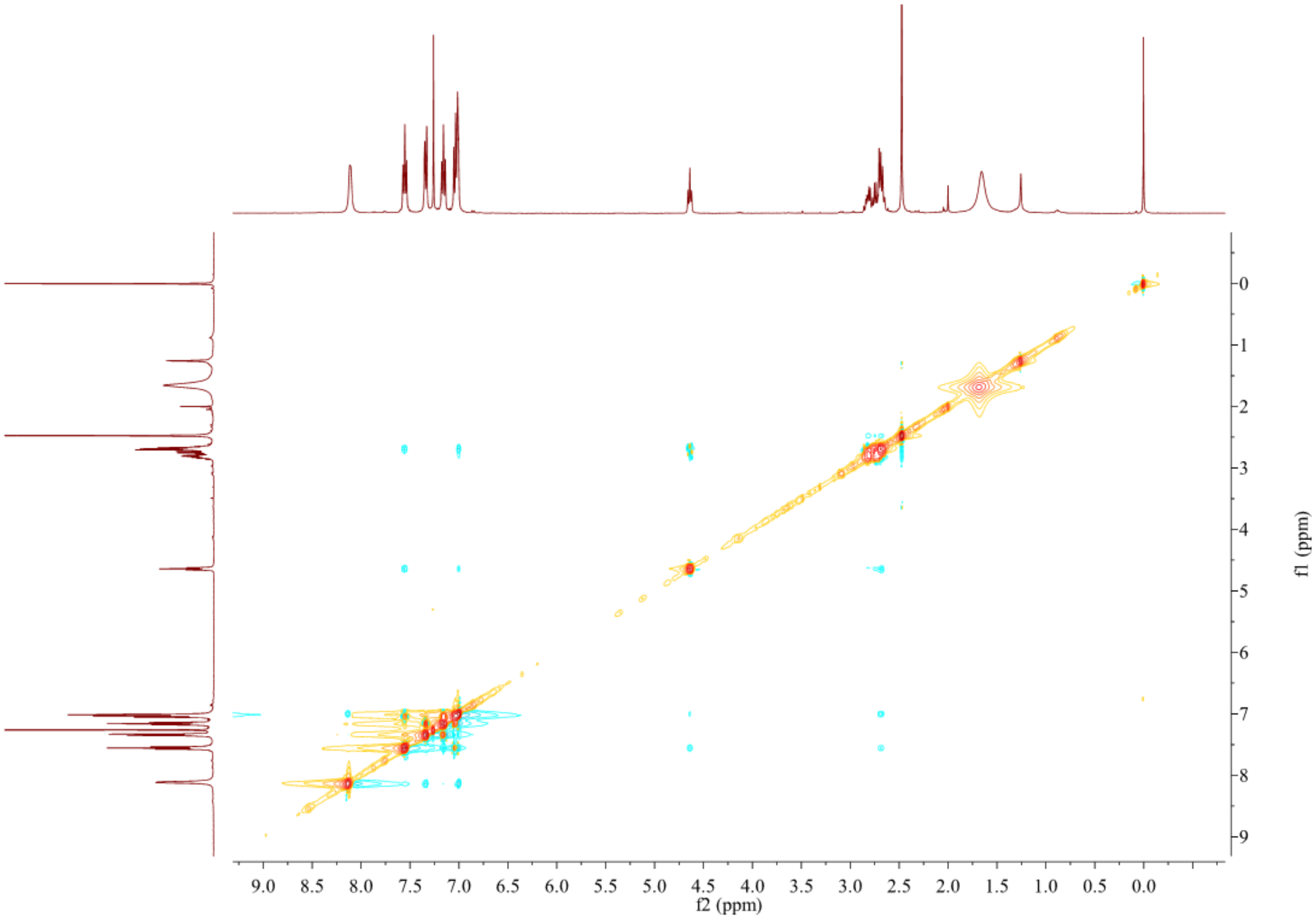

Figure S19. NOESY spectrum of pseudboindole B (**2**) in $CDCl_3$.

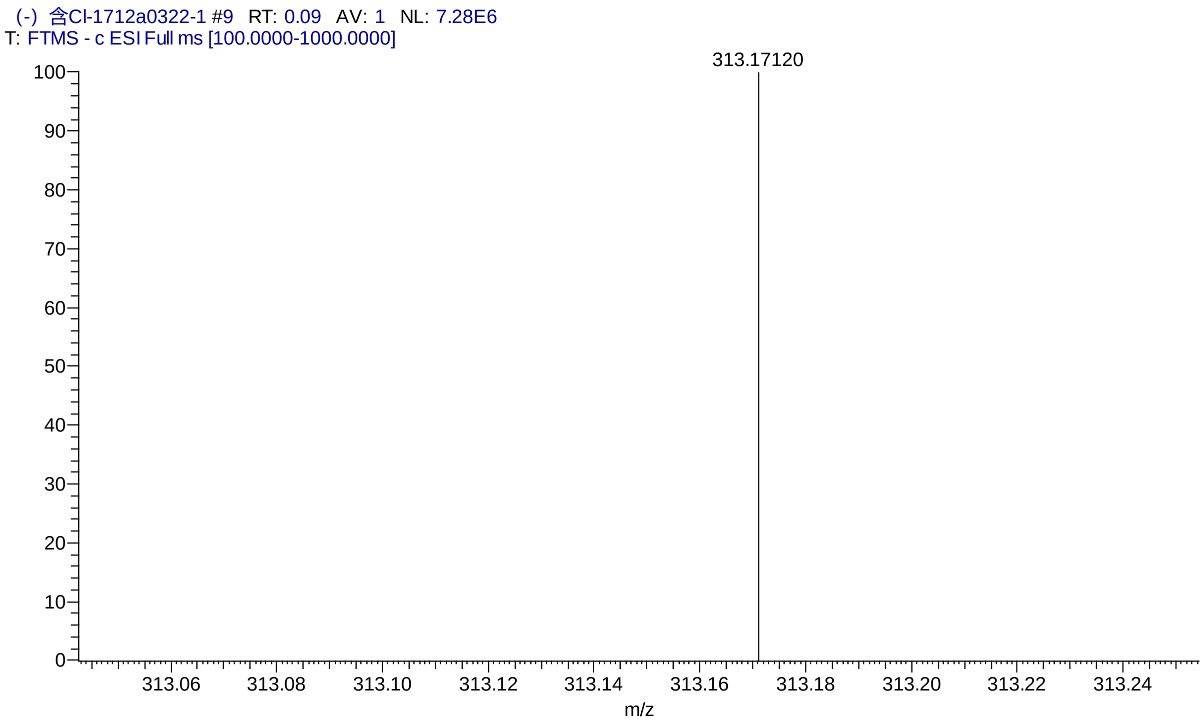

SPECTRUM - simulation:

m/z	Theo. Mass	Delta (ppm)	RDB equiv.	Composition
313.17120	313.17102	0.57	13.5	C22 H21 N2

Figure S20. HR-(-)ESI-MS spectrum of 3,3'-cyclohexylidenebis(1*H*-indole) (**3**).

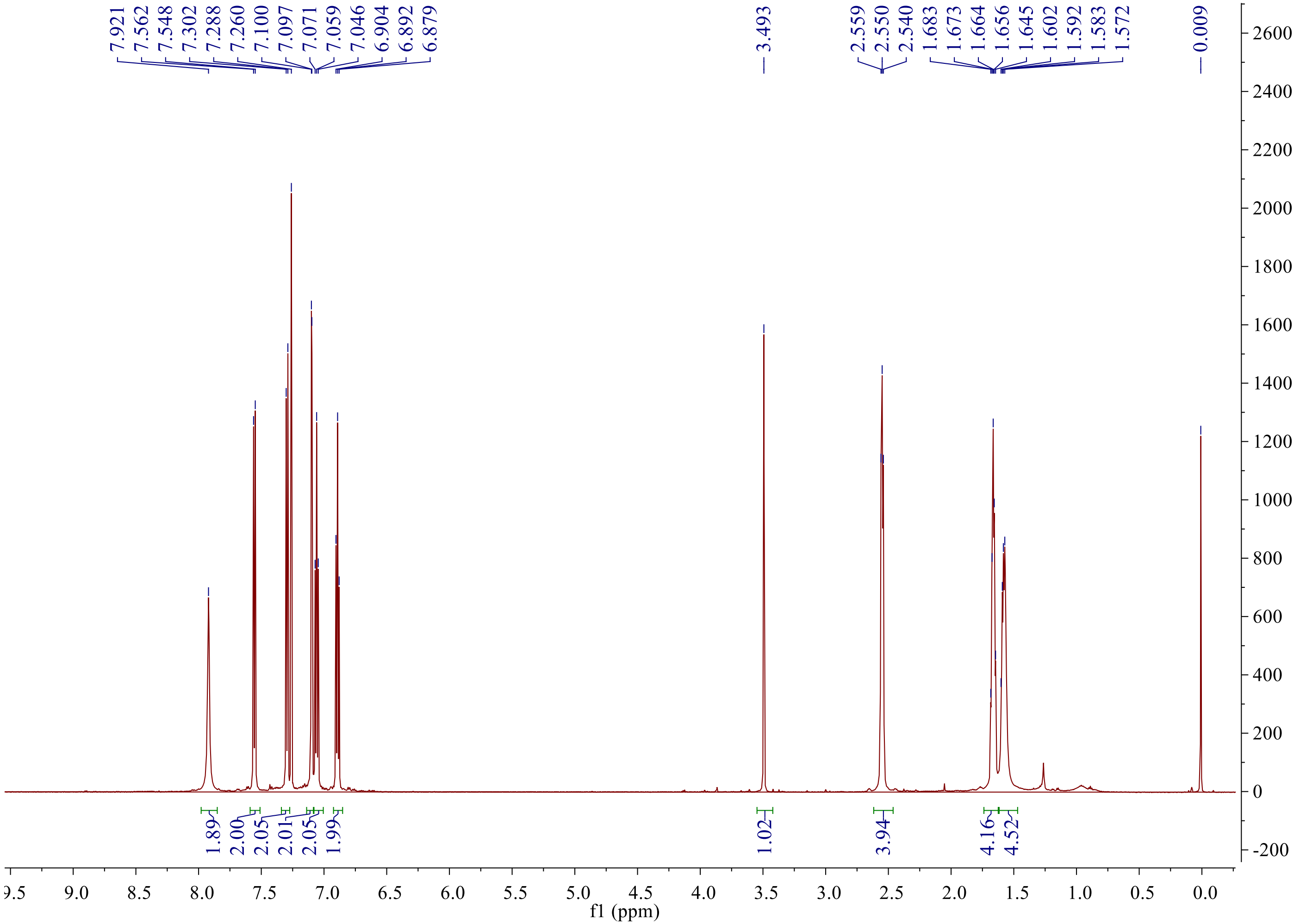

Figure S21. ^{1}H NMR spectrum of 3,3'-cyclohexylidenebis(1*H*-indole) (**3**) in $CDCl_3$ (600 MHz).

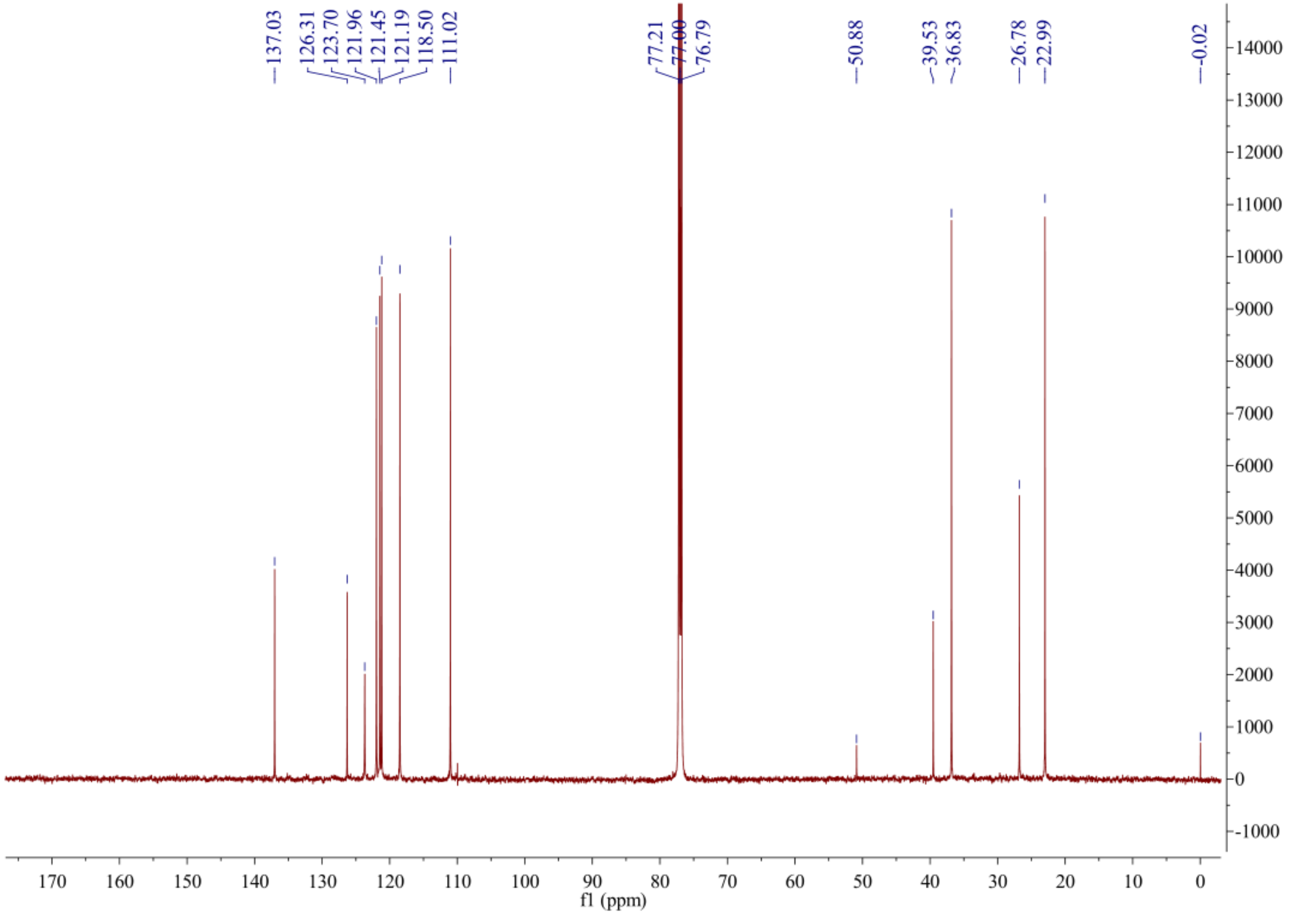

Figure S22. ^{13}C NMR spectrum of 3,3'-cyclohexylidenebis(1*H*-indole) (**3**) in $CDCl_3$ (150 MHz).

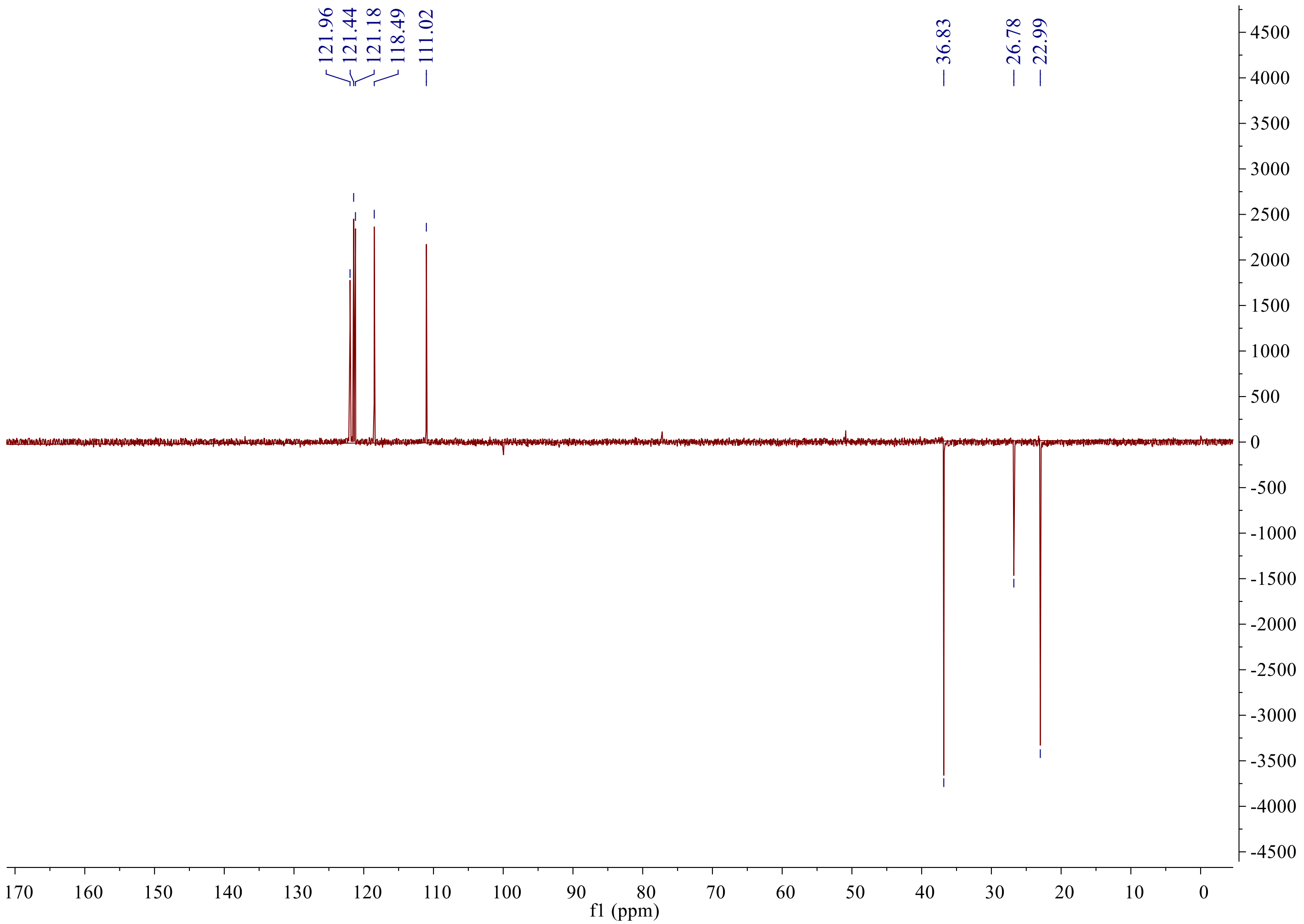

Figure S23. DEPT 135 spectrum of 3,3'-cyclohexylidenebis(1*H*-indole) (**3**) in $CDCl_3$ (150 MHz).

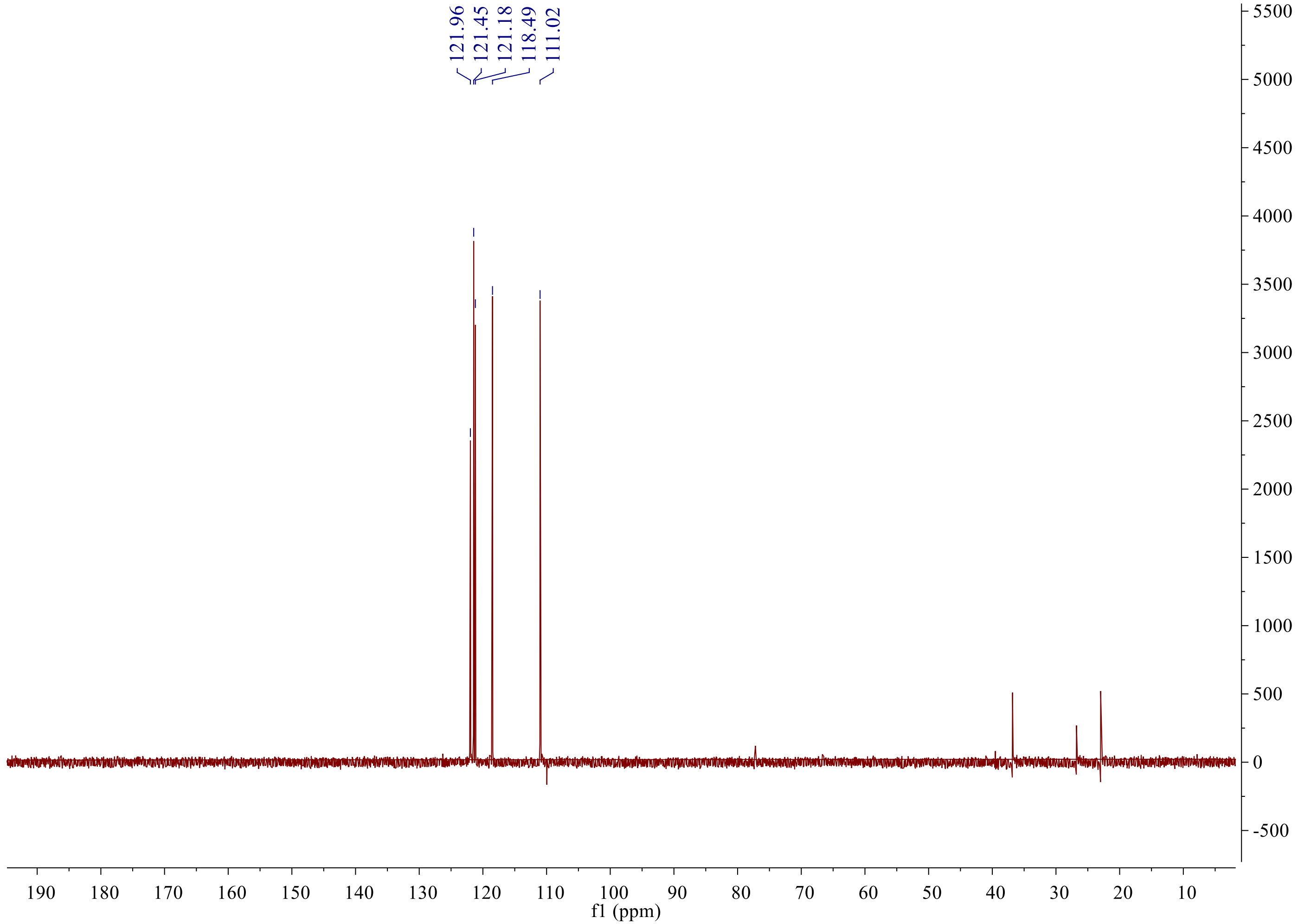

Figure S24. DEPT 90 spectrum of 3,3'-cyclohexylidenebis(1*H*-indole) (**3**) in $CDCl_3$ (150 MHz).

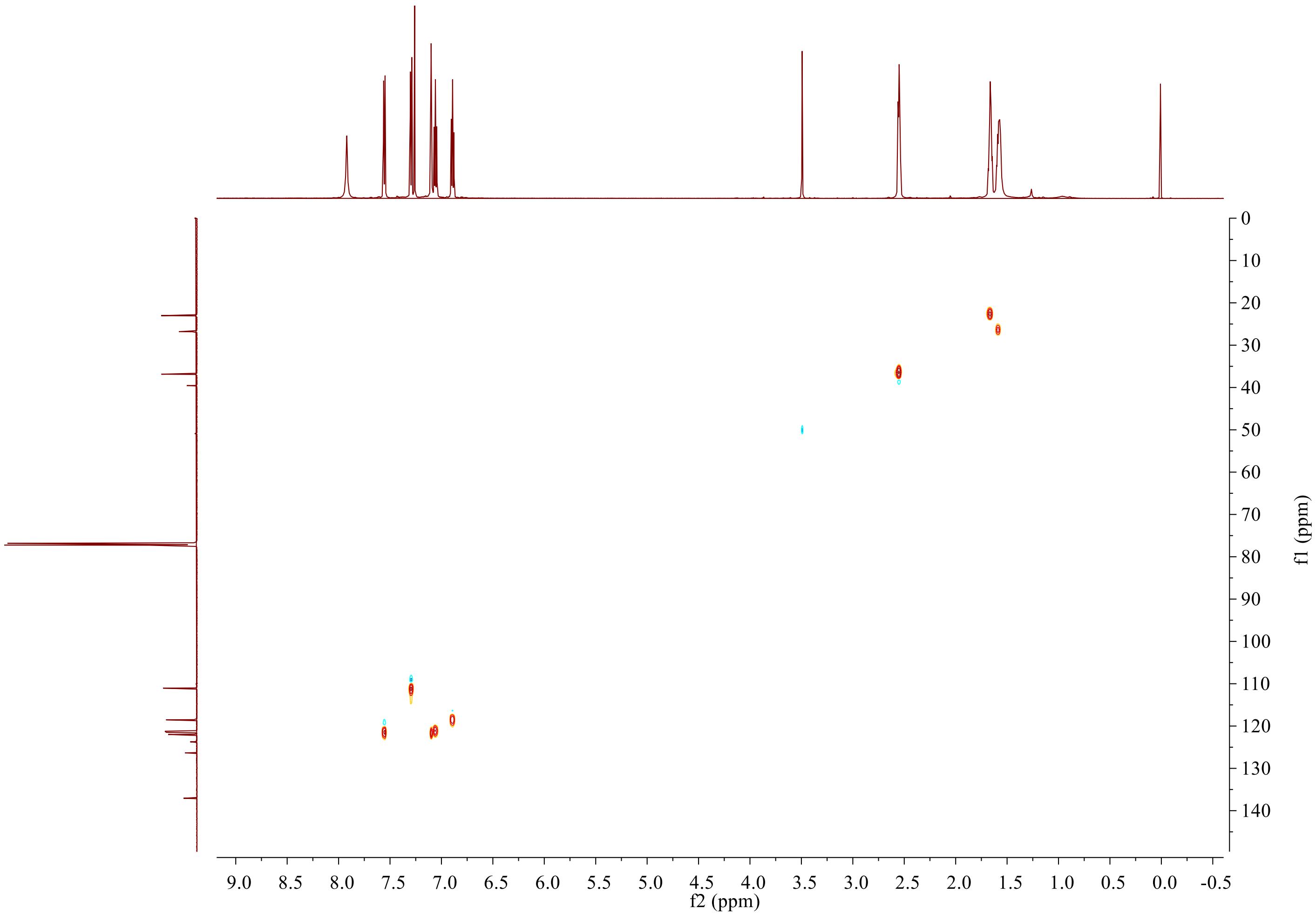

Figure S25. HMQC spectrum of 3,3'-cyclohexylidenebis(1*H*-indole) (**3**) in $CDCl_3$.

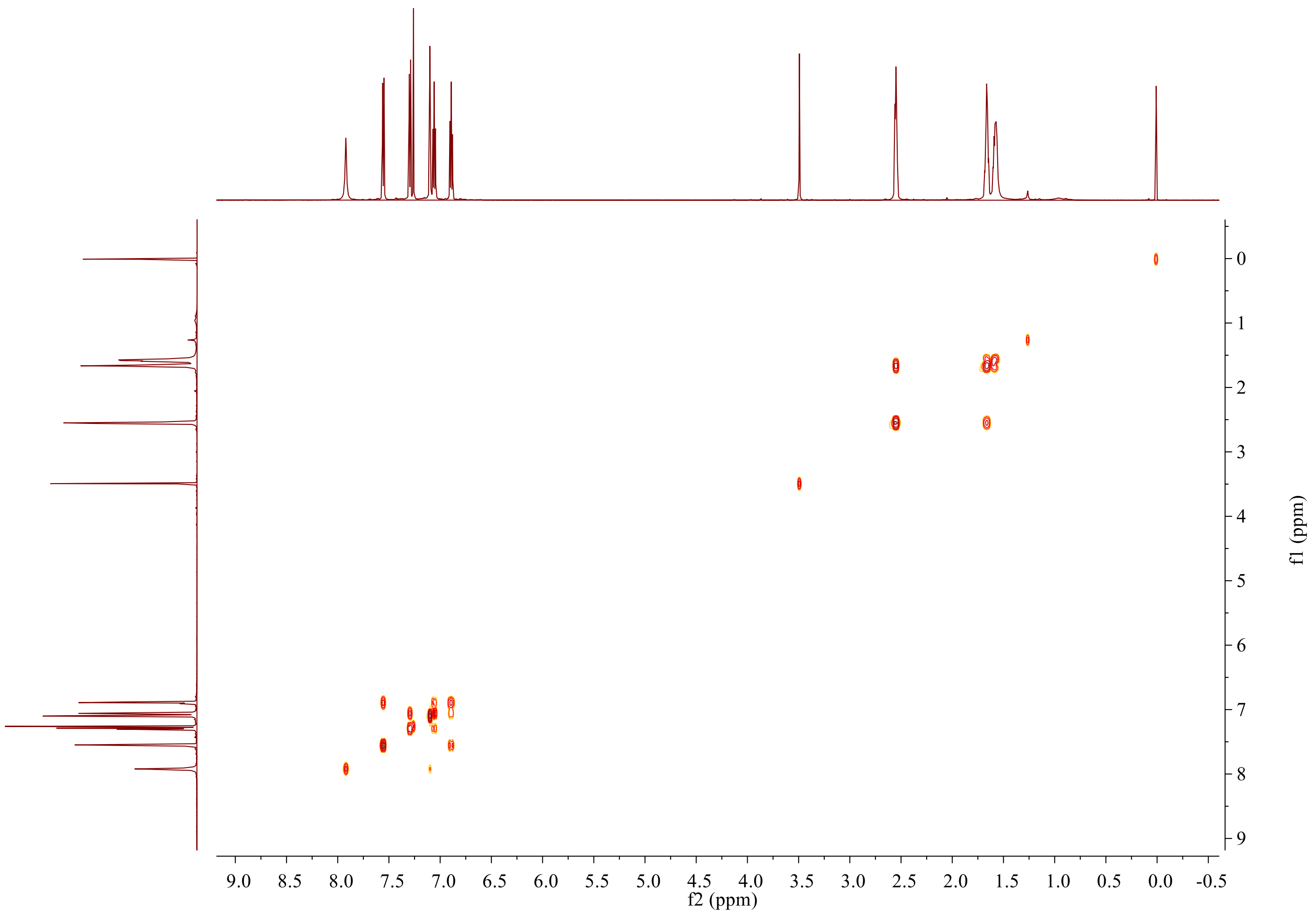

Figure S26. ^{1}H–^{1}H COSY spectrum of 3,3'-cyclohexylidenebis(1*H*-indole) (**3**) in $CDCl_3$.

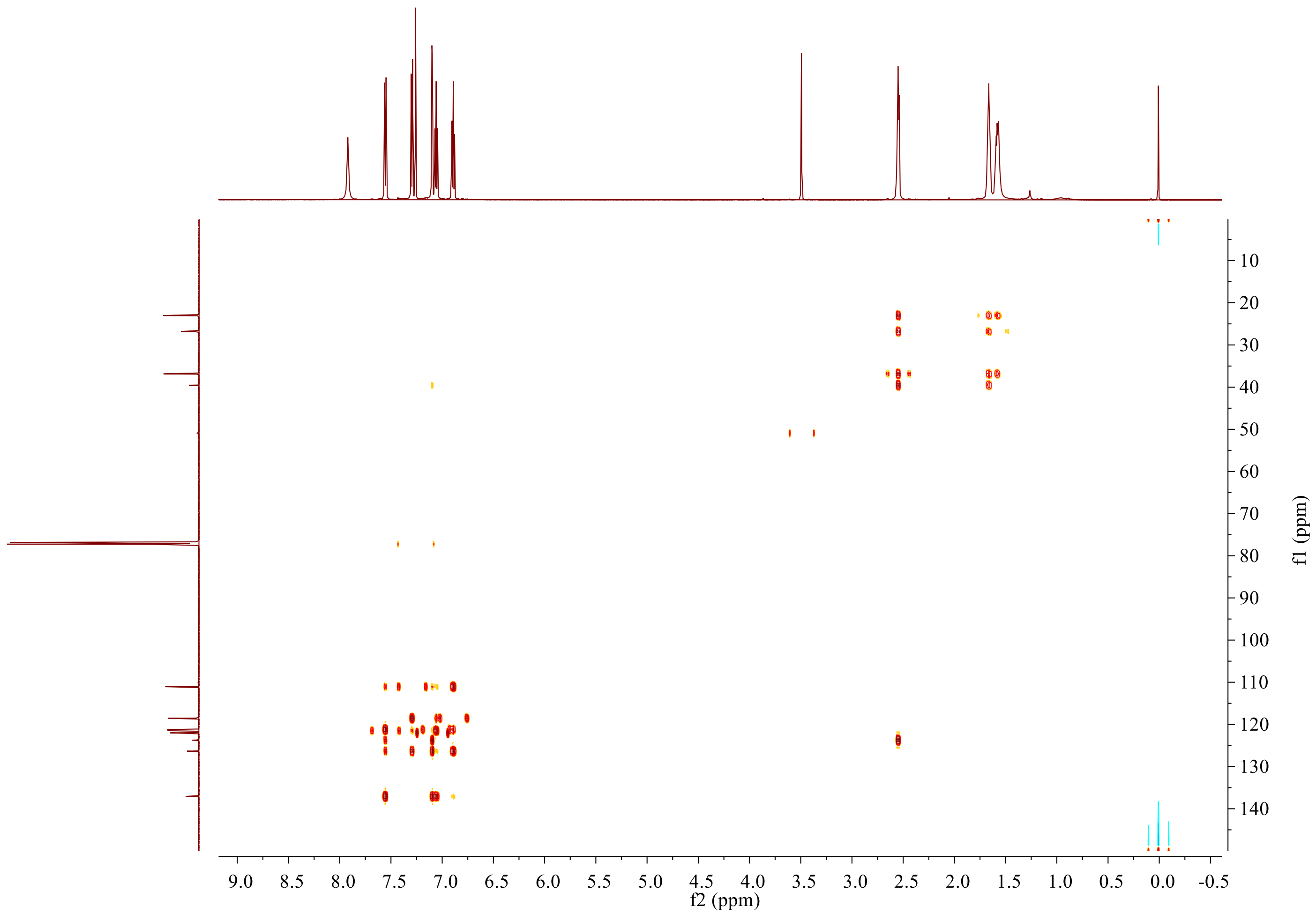

Figure S27. HMBC spectrum of 3,3'-cyclohexylidenebis(1*H*-indole) (**3**) in $CDCl_3$.

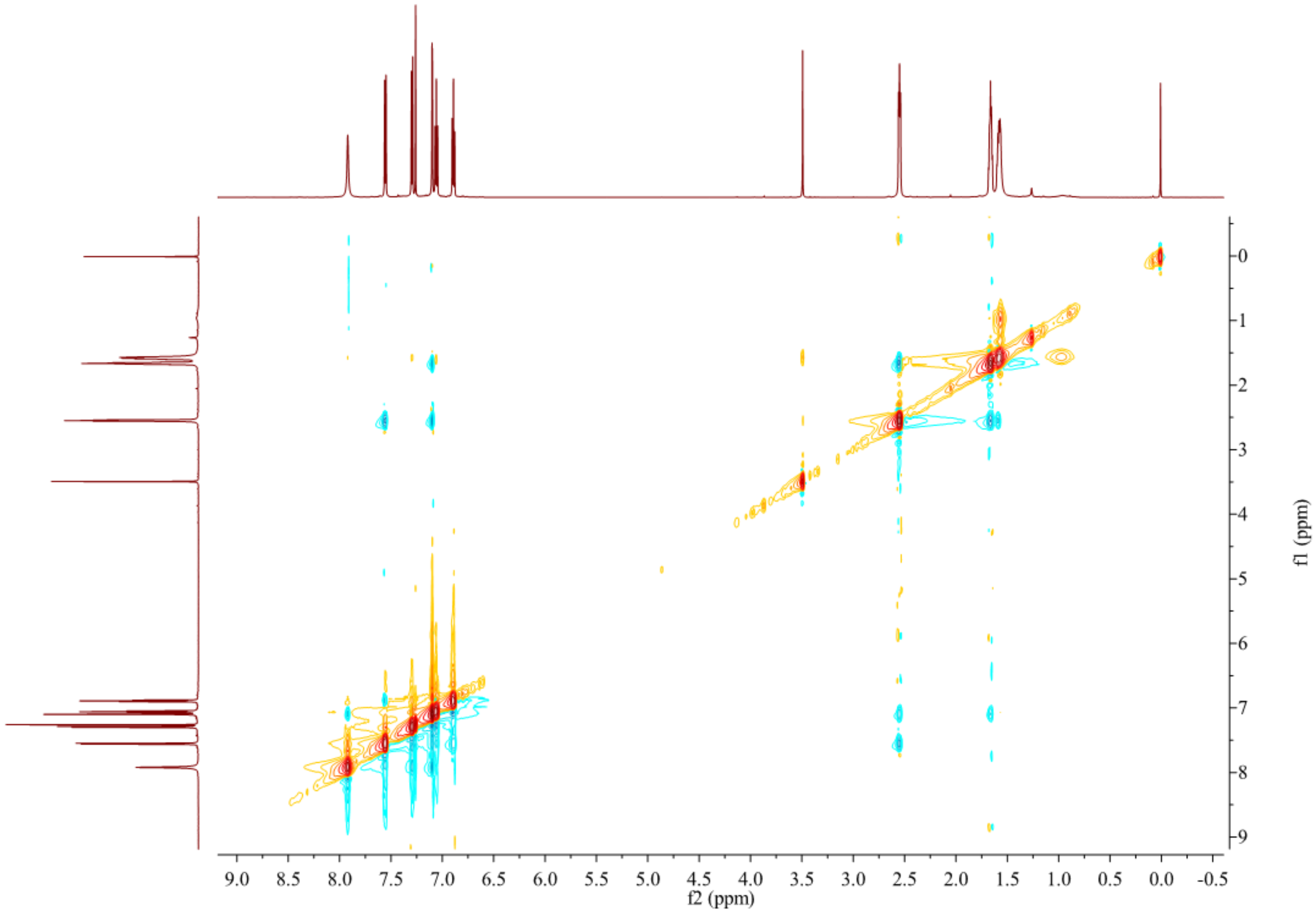

Figure S28. NOESY spectrum of 3,3'-cyclohexylidenebis(1*H*-indole) (**3**) in $CDCl_3$.

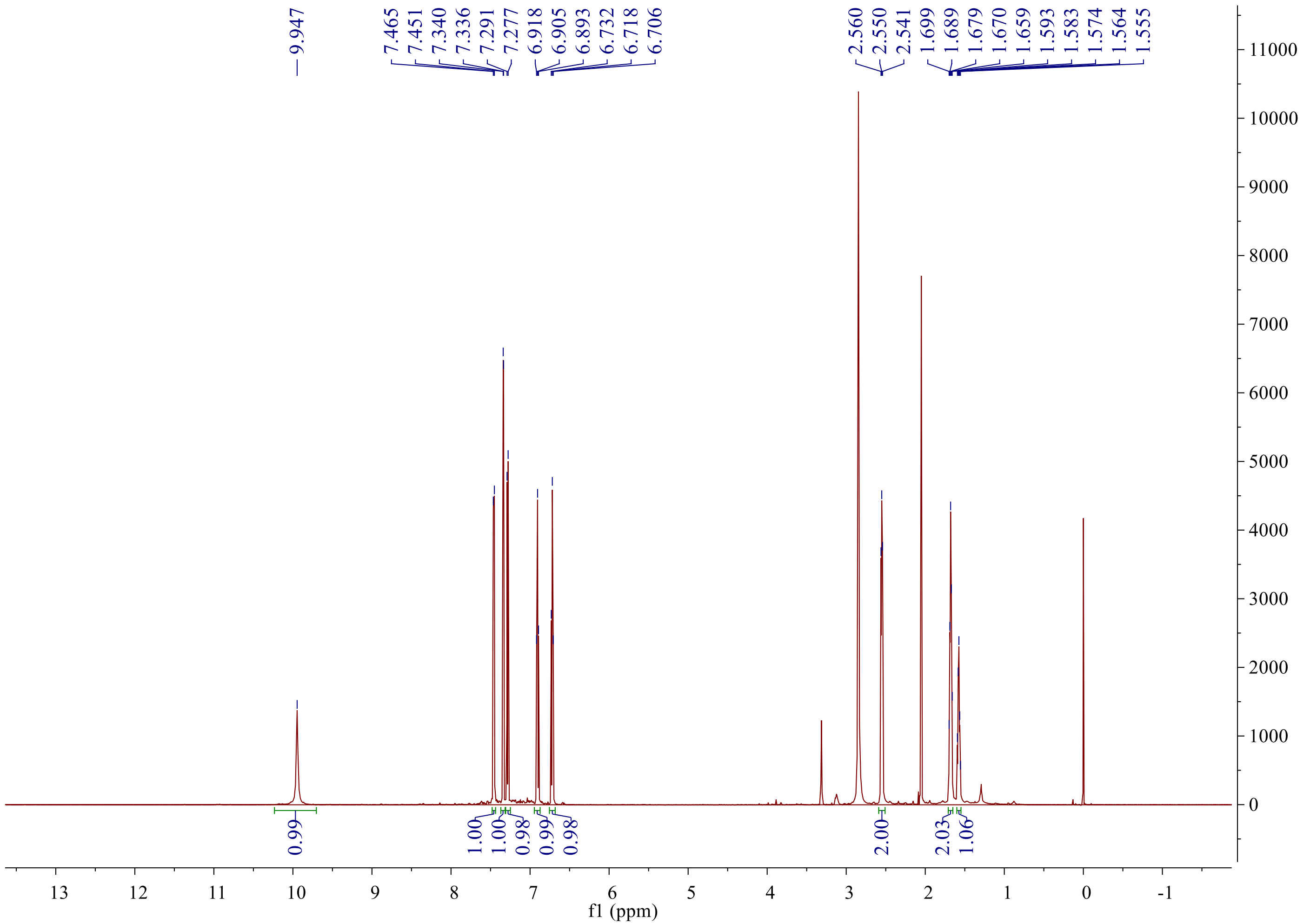

Figure S29. ^{1}H NMR spectrum of 3,3'-cyclohexylidenebis(1*H*-indole) (**3**) in acetone-d_6 (600 MHz).

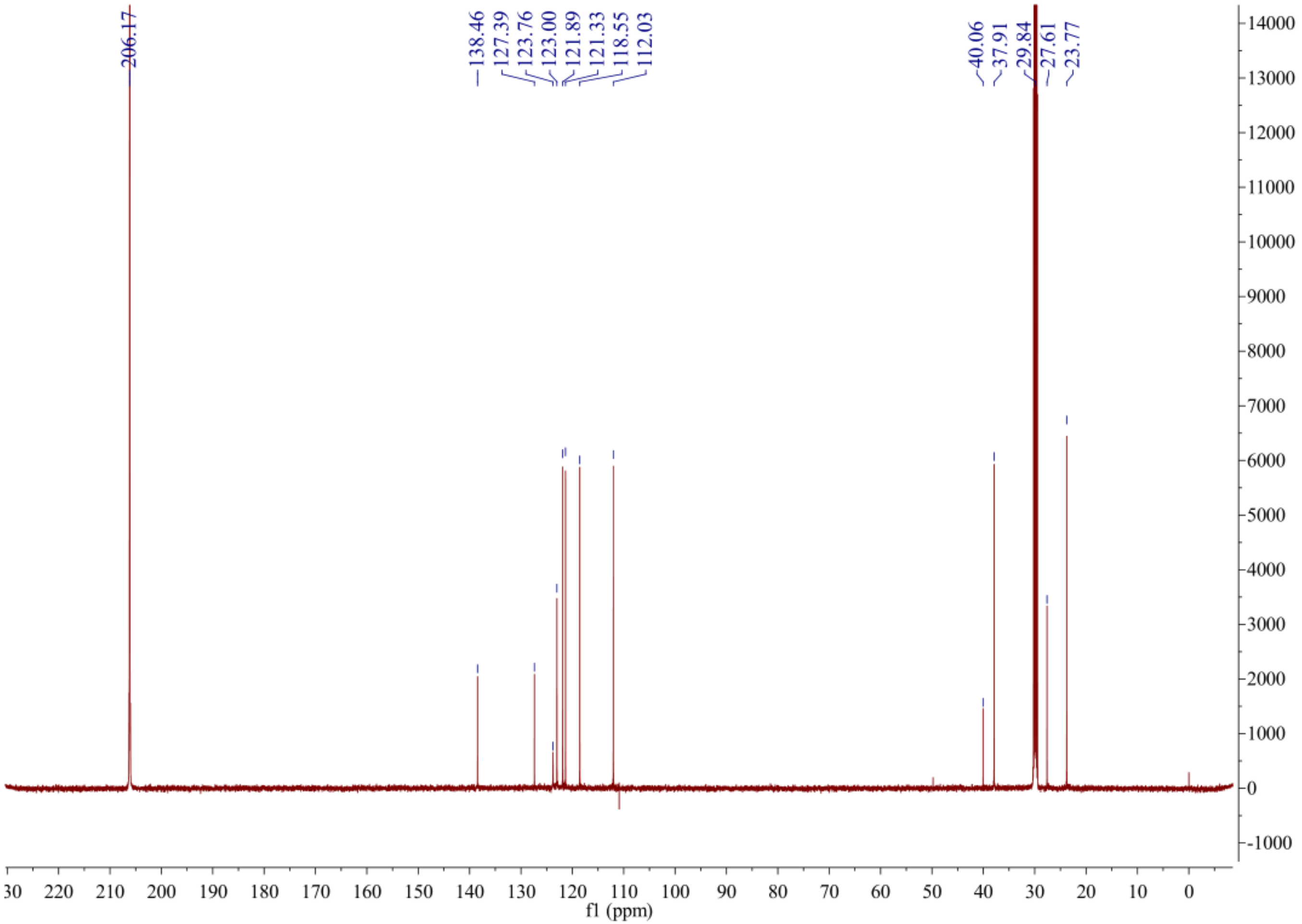

Figure S30. ^{13}C NMR spectrum of 3,3'-cyclohexylidenebis(1*H*-indole) (**3**) in acetone-d_6 (150 MHz).

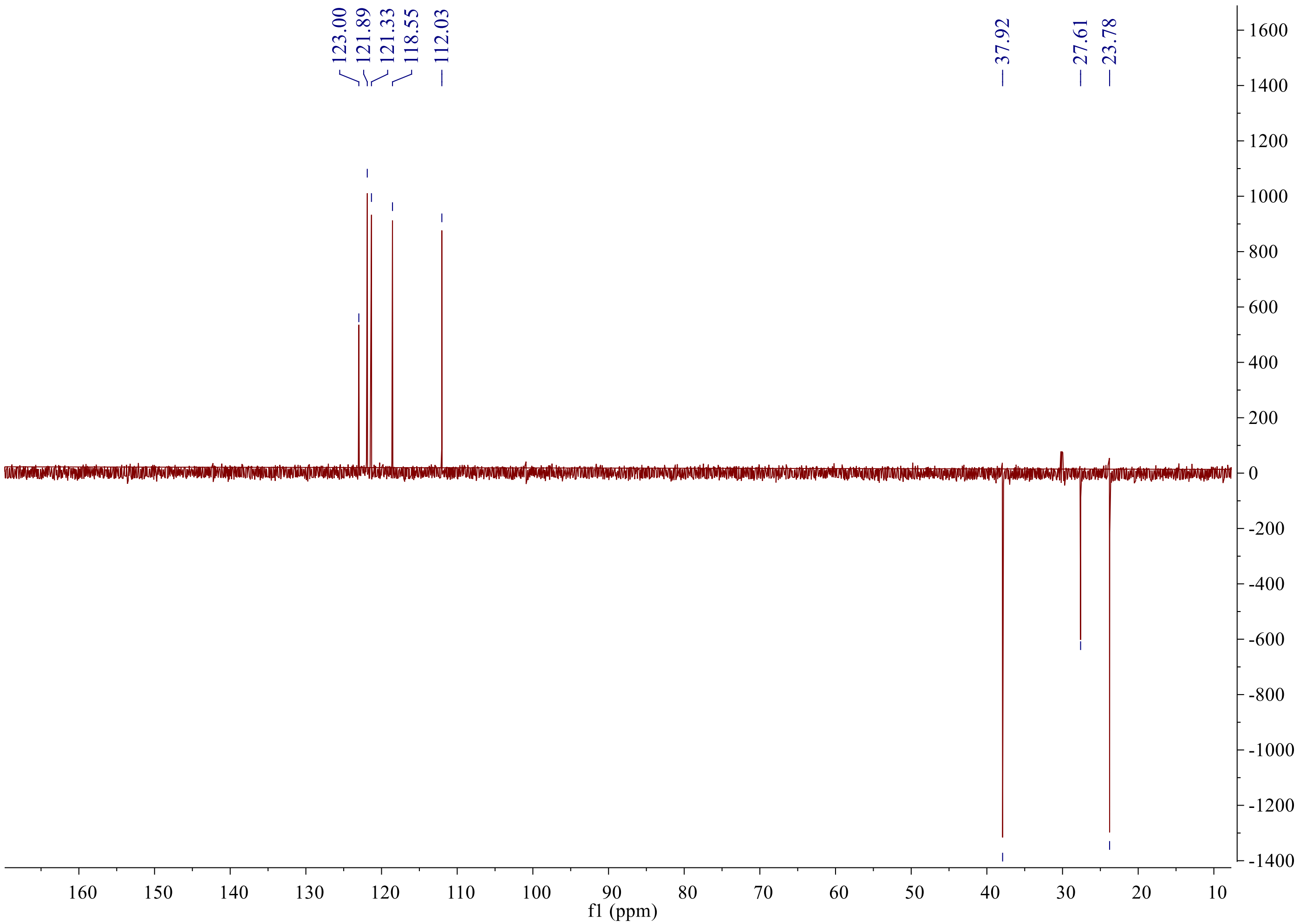

Figure S31. DEPT 135 spectrum of 3,3'-cyclohexylidenebis(1*H*-indole) (**3**) in acetone-d_6 (150 MHz).

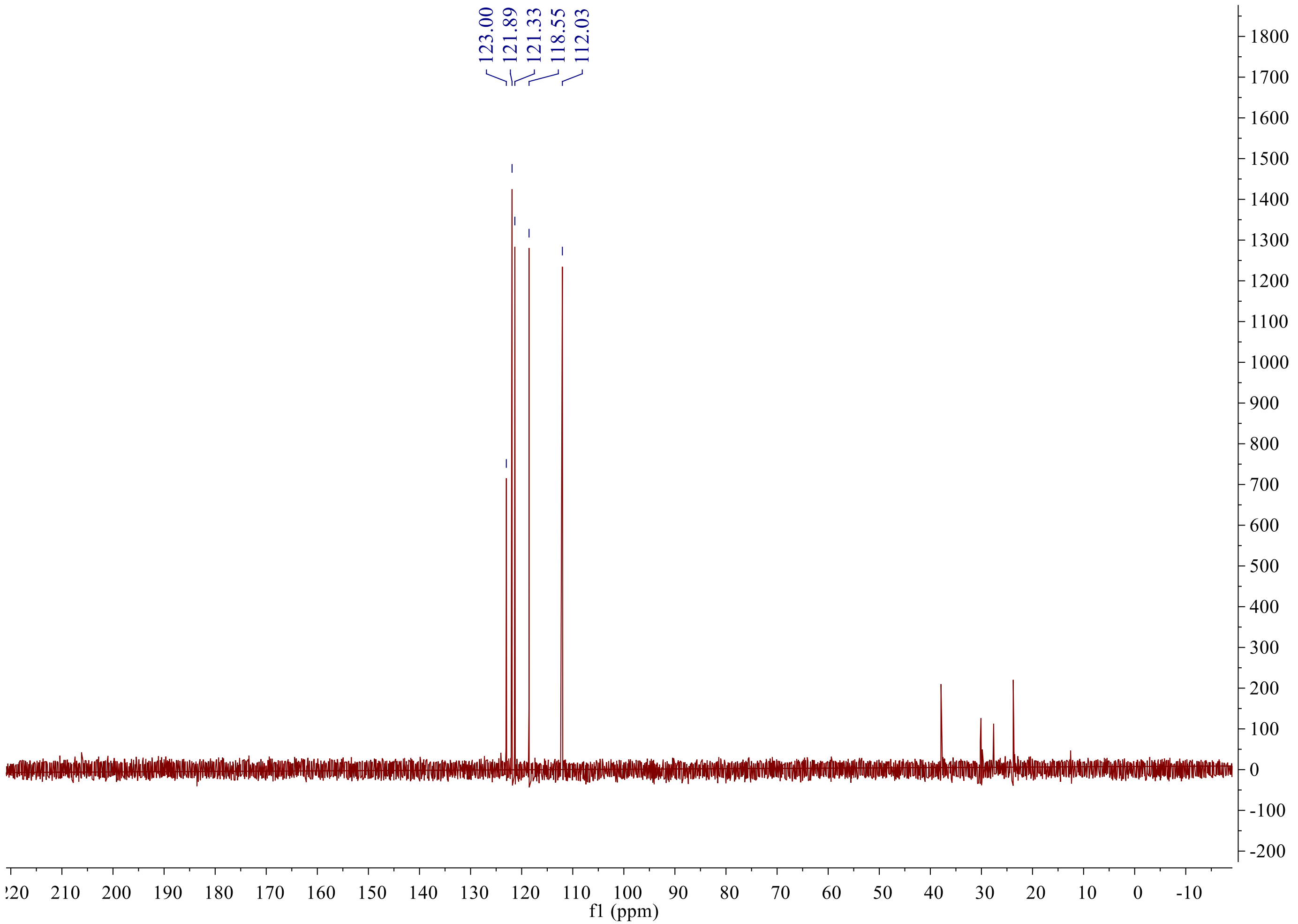

Figure S32. DEPT 90 spectrum of 3,3'-cyclohexylidenebis(1*H*-indole) (**3**) in acetone-d_6 (150 MHz).

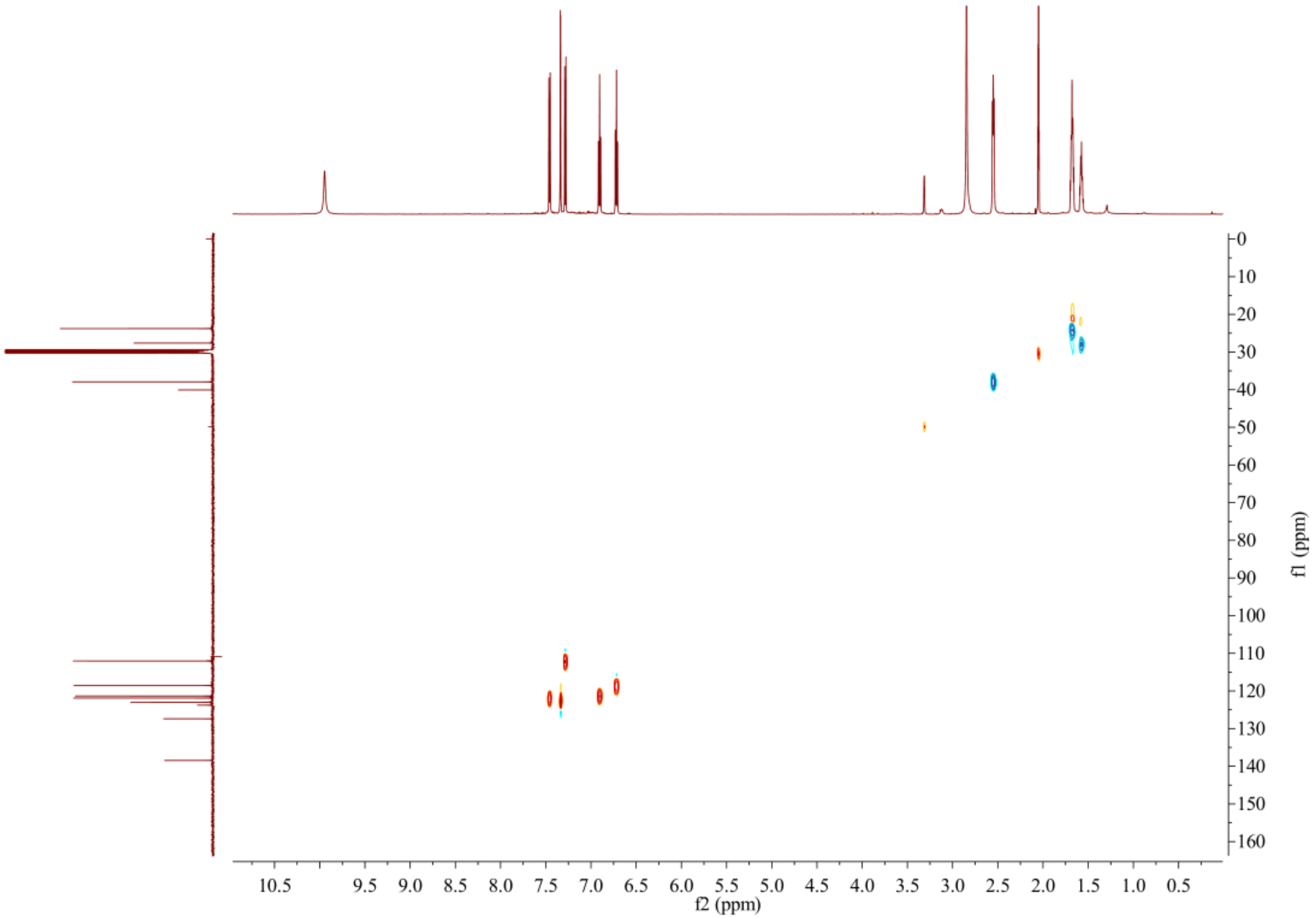

Figure S33. HMQC spectrum of 3,3'-cyclohexylidenebis(1*H*-indole) (**3**) in acetone-d_6.

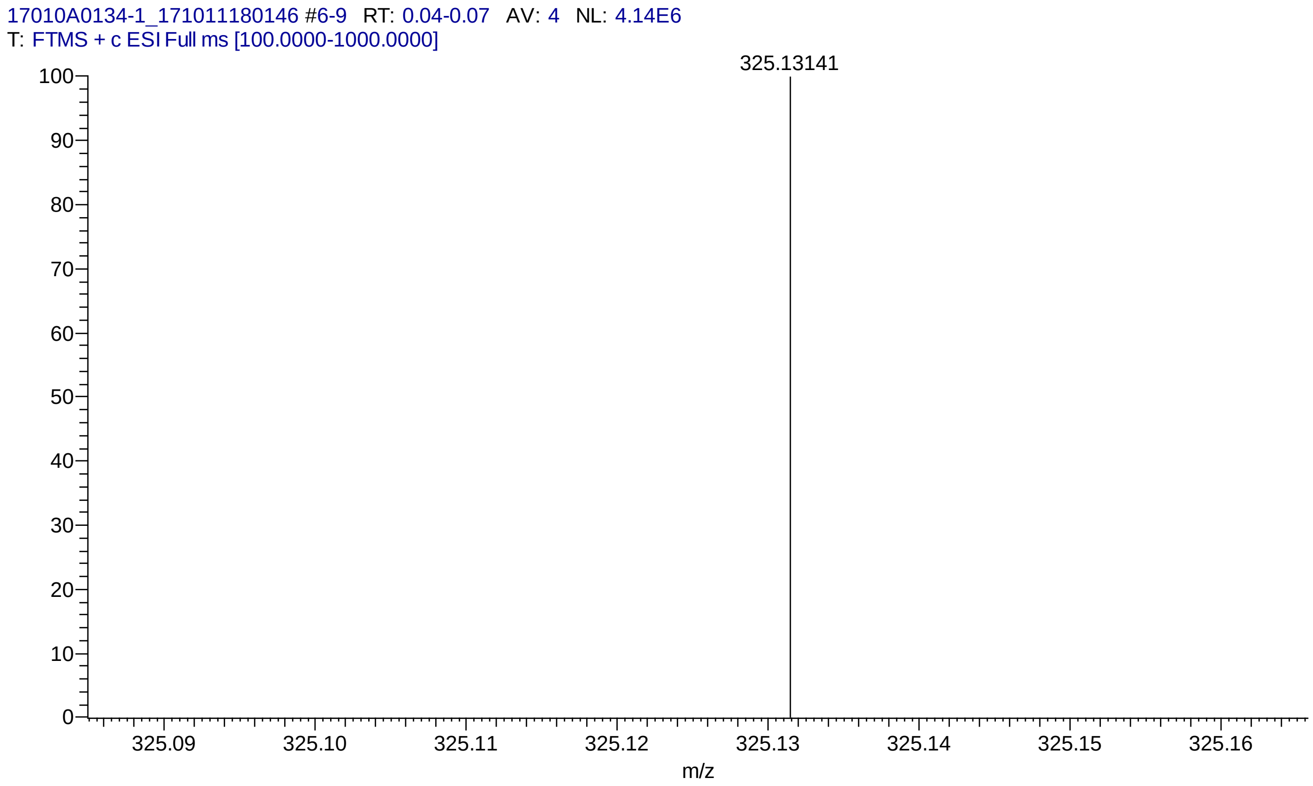

SPECTRUM - simulation:

m/z	Theo. Mass	Delta (ppm)	RDB equiv.	Composition
325.1314	325.13113	0.85	12.5	C20 H18 O N2 Na

Figure S34. HR-(+)ESI-MS spectrum of 3,3-bis(3-indolyl) butan-2-one (**4**).

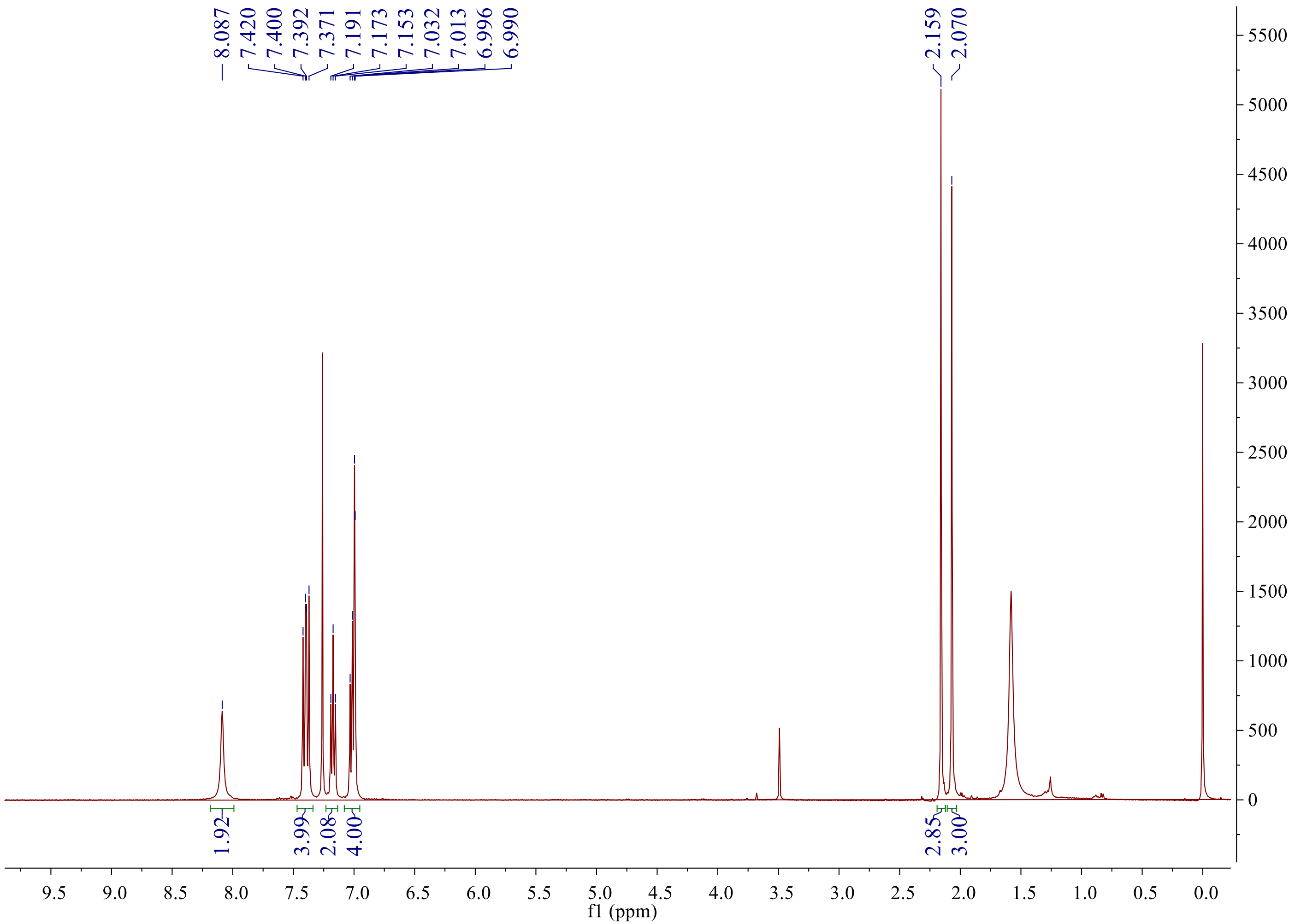

Figure S35. ^{1}H NMR spectrum of 3,3- bis(3-indolyl) butan-2-one (**4**) in $CDCl_3$ (400 MHz).

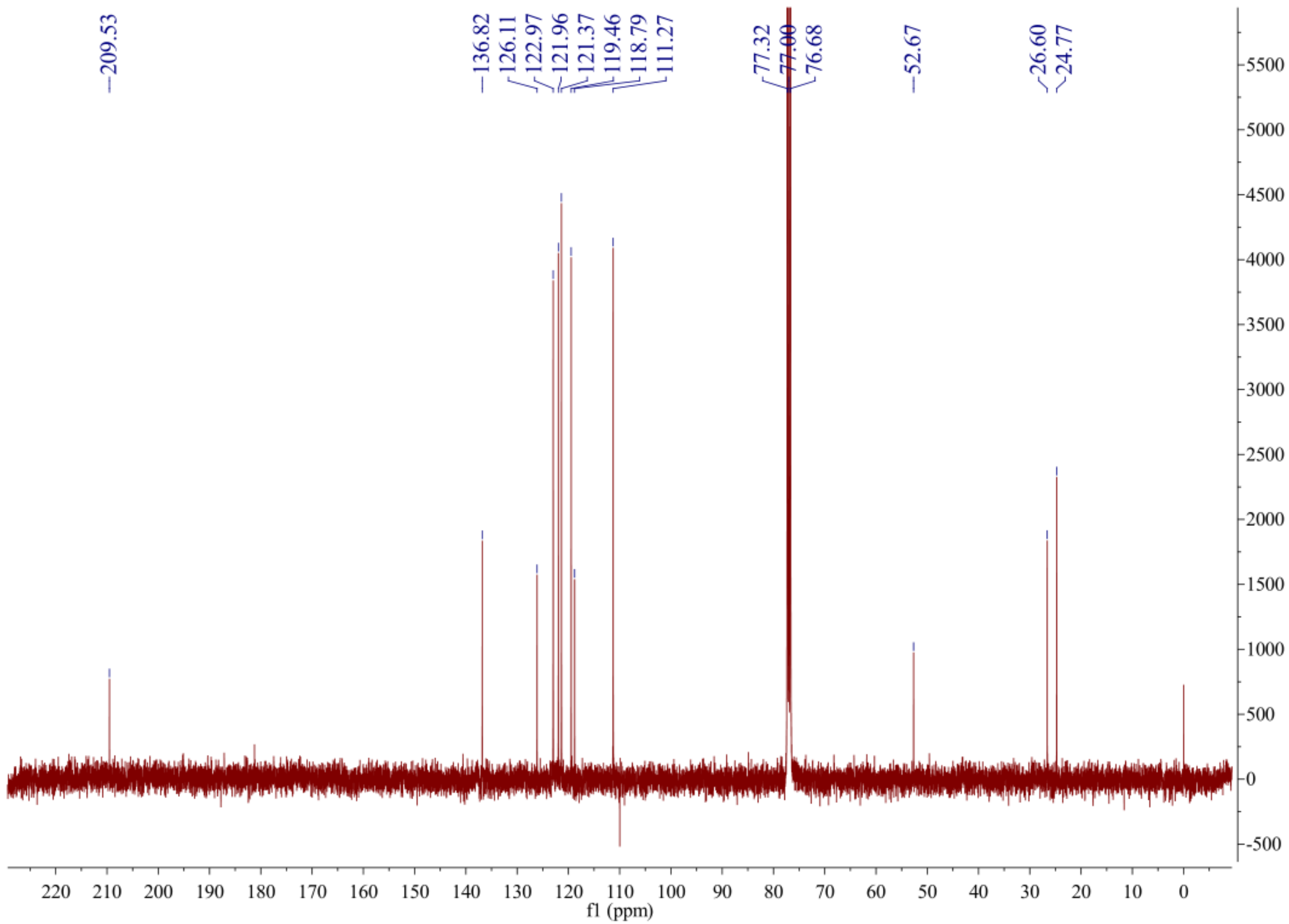

Figure S36. ^{13}C NMR spectrum of 3,3- bis(3-indolyl) butan-2-one (**4**) in $CDCl_3$ (100 MHz).

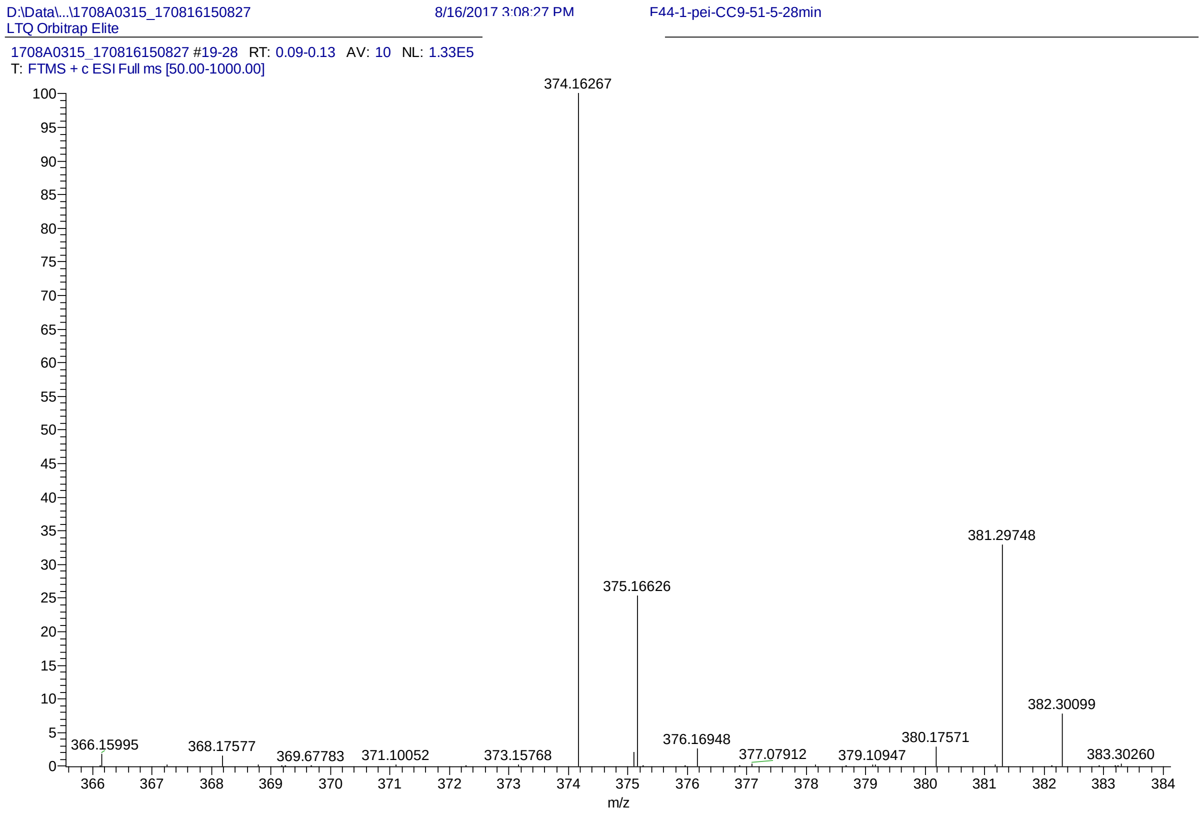

SPECTRUM - simulation:

m/z	Theo. Mass	Delta (ppm)	RDB equiv.	Composition
374.16267	374.16277	−0.26	15.5	C24 H21 N3 Na

Figure S37. HR-(+)ESI-MS spectrum of 2-[2,2-di(1*H*-indol-3-yl) ethyl] aniline (**5**).

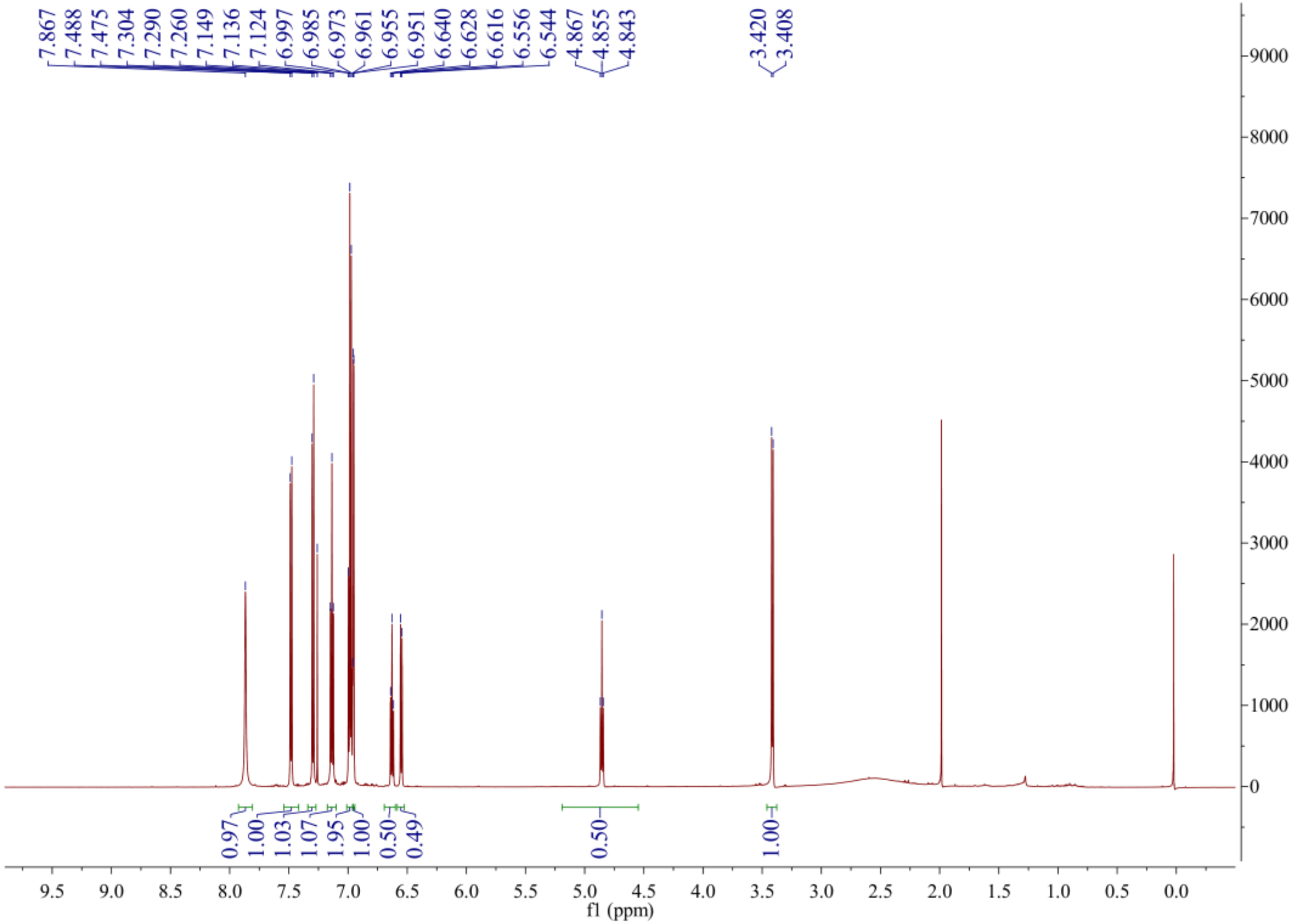

Figure S38. ^{1}H NMR spectrum of 2-[2,2-di(1*H*-indol-3-yl) ethyl] aniline (**5**) in $CDCl_3$ (600 MHz).

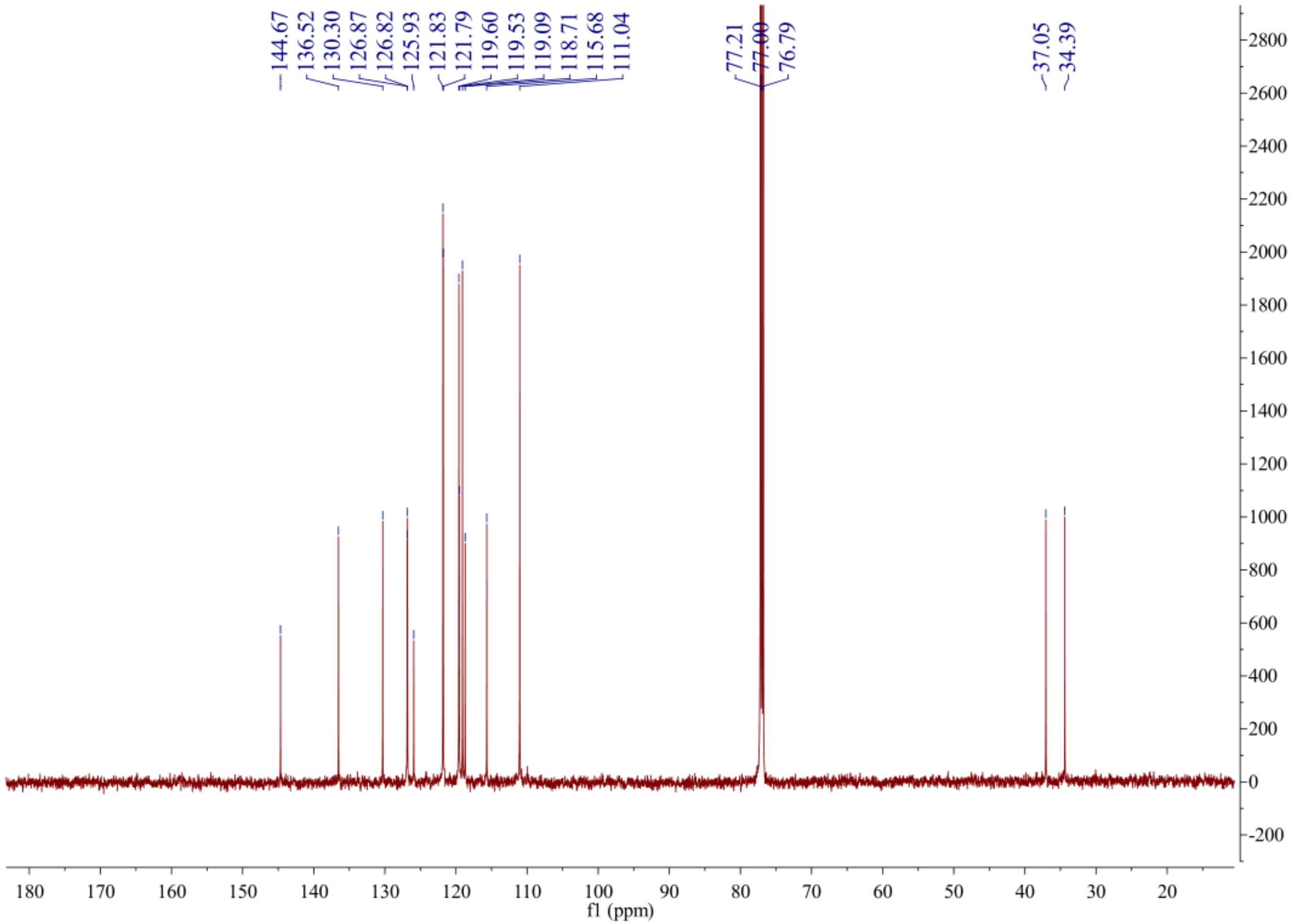

Figure S39. ^{13}C NMR spectrum of 2-[2,2-di(1*H*-indol-3-yl) ethyl] aniline (**5**) in $CDCl_3$ (150 MHz).

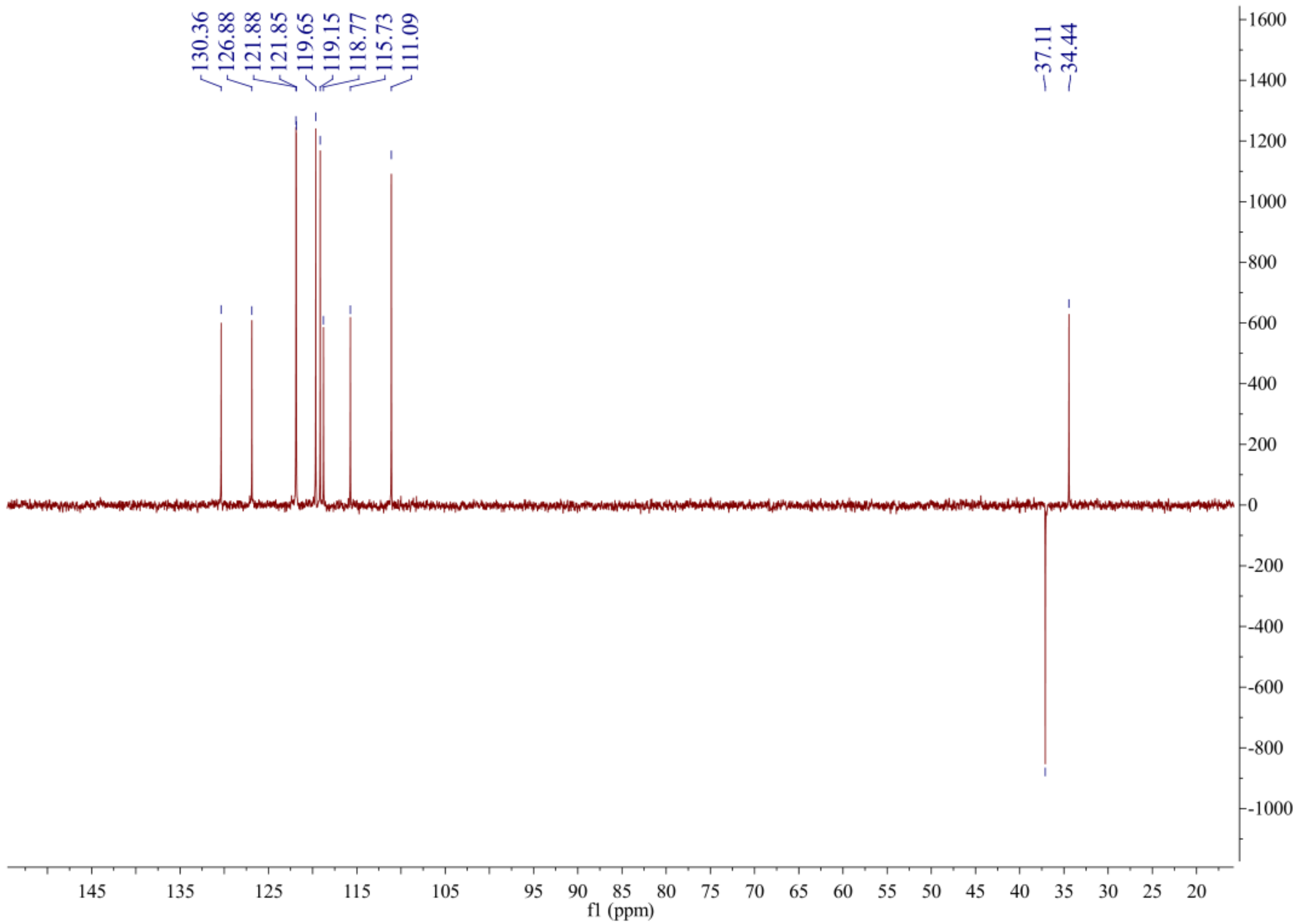

Figure S40. DEPT 135 spectrum of 2-[2,2-di(1*H*-indol-3-yl) ethyl] aniline (**5**) in $CDCl_3$ (150 MHz).

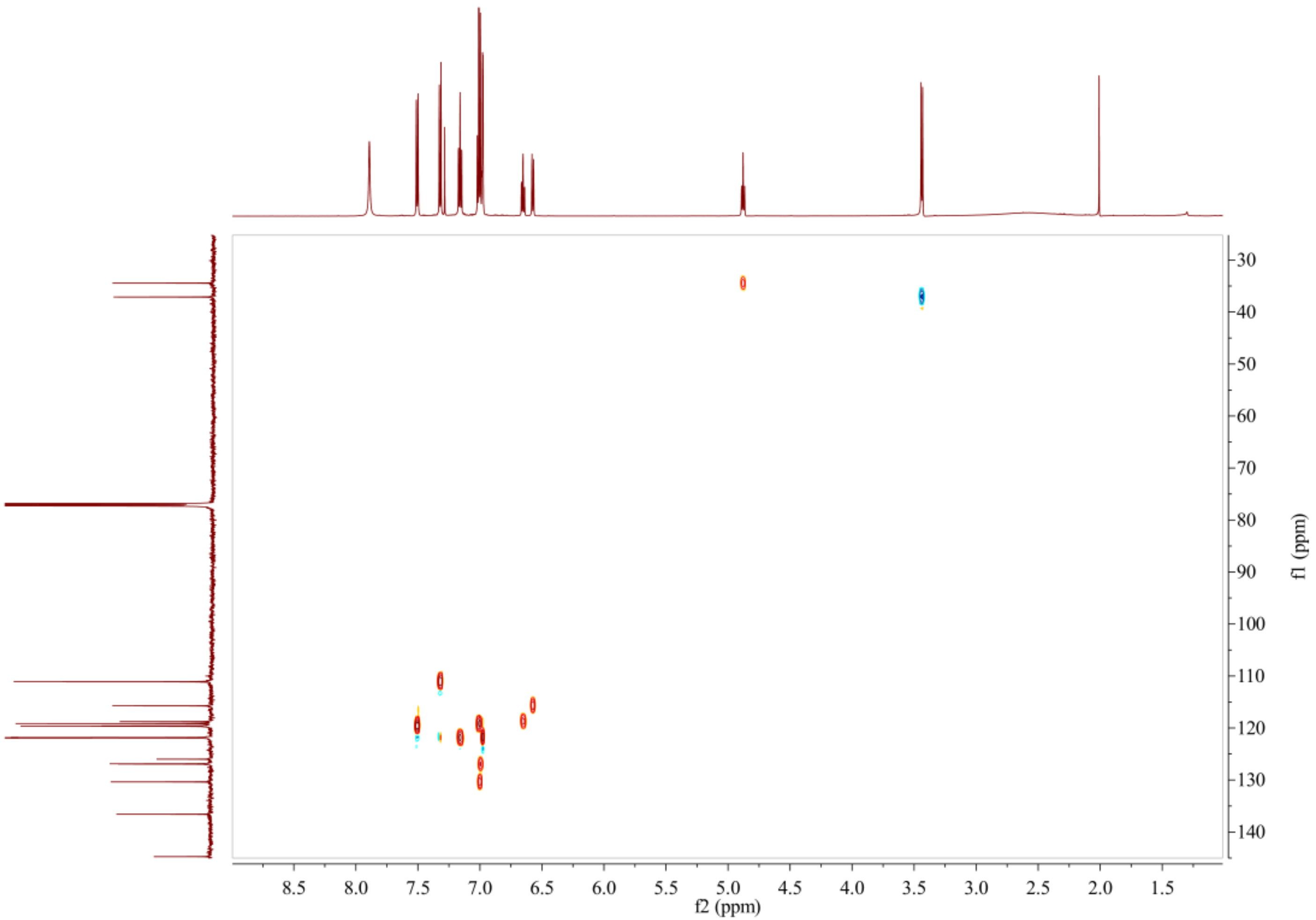

Figure S41. HMQC spectrum of 2-[2,2-di(1*H*-indol-3-yl) ethyl] aniline (**5**) in $CDCl_3$.

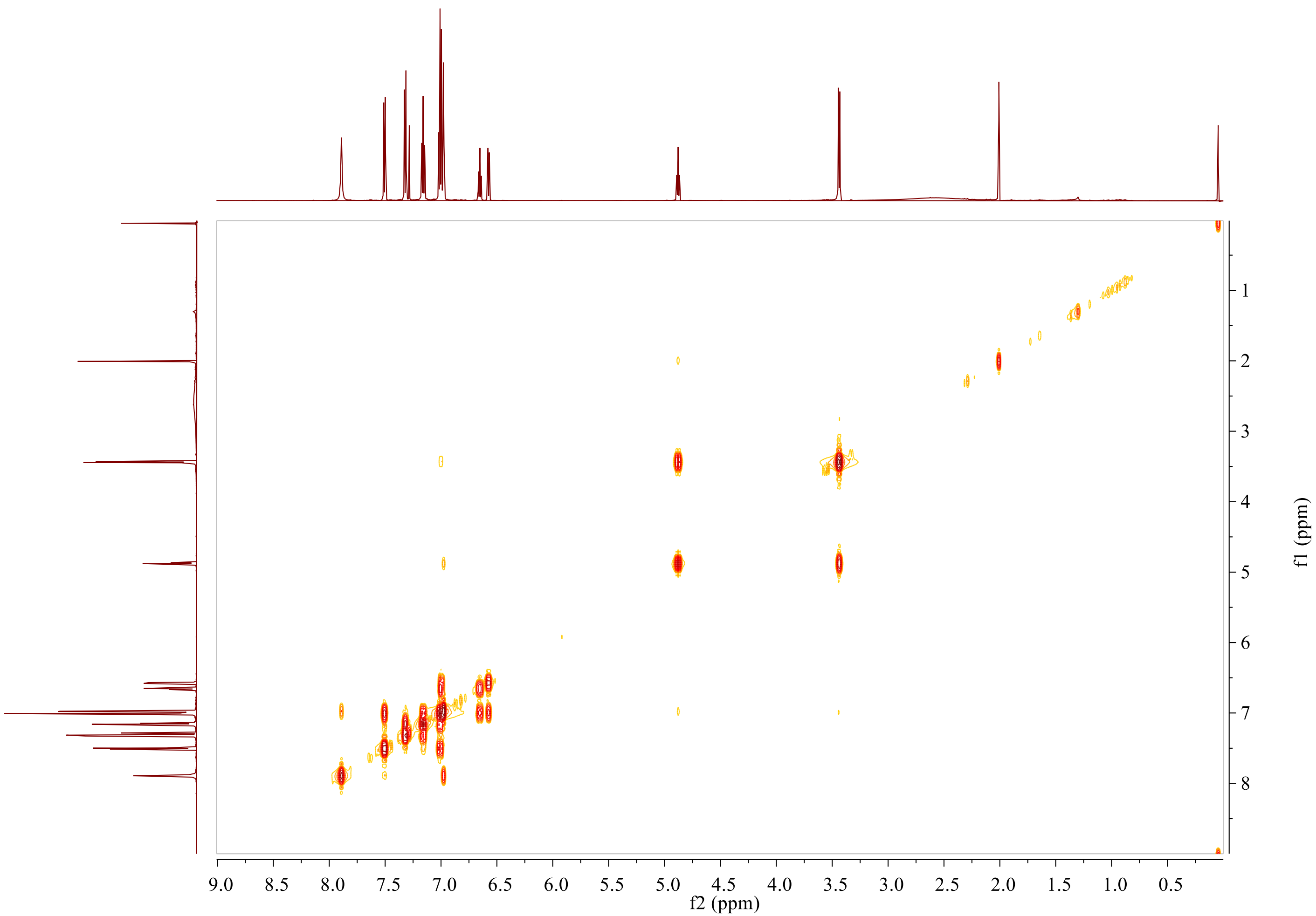

Figure S42. ^{1}H–^{1}H COSY spectrum of 2-[2,2-di(1*H*-indol-3-yl) ethyl] aniline (**5**) in $CDCl_3$.

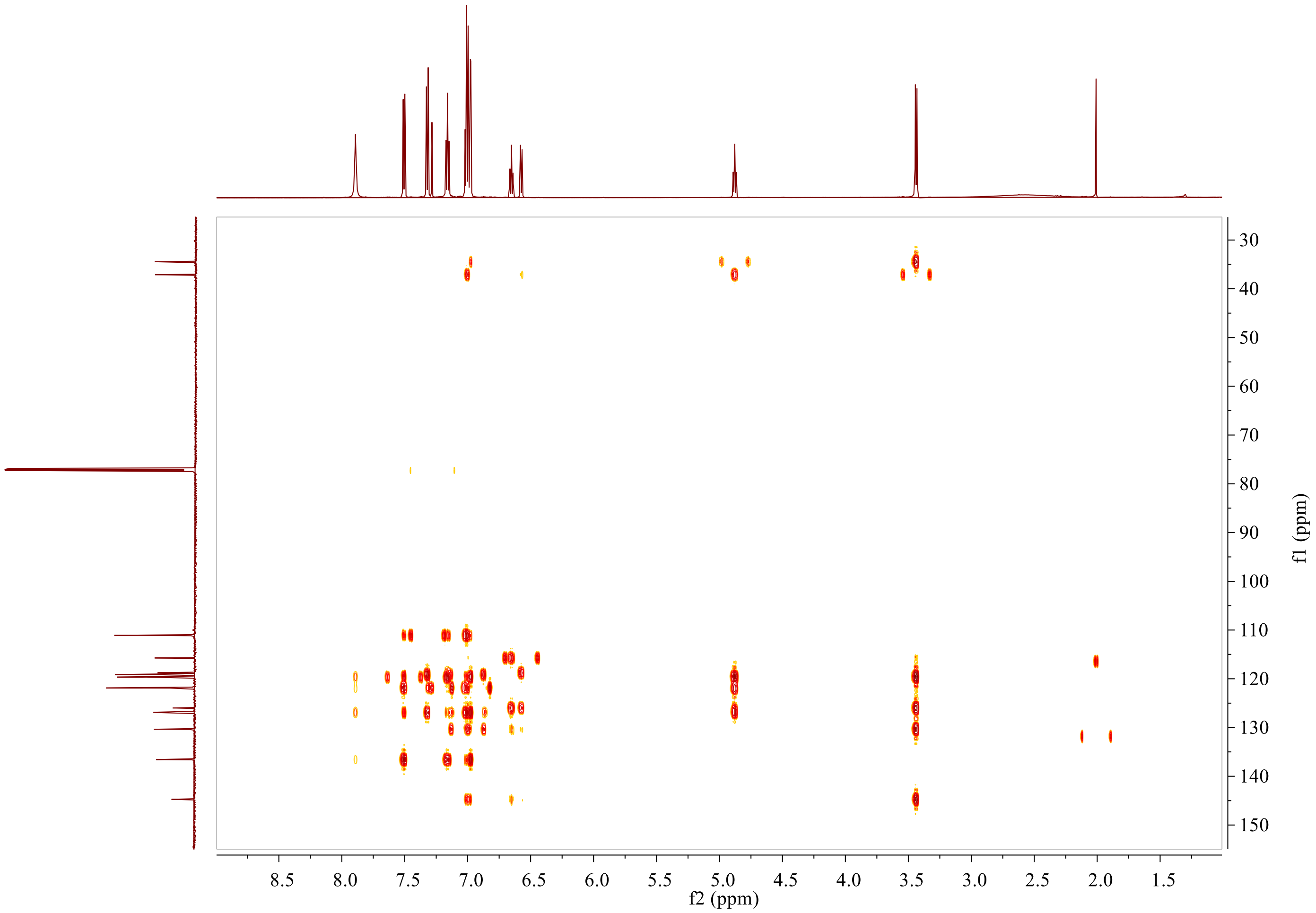

Figure S43. HMBC spectrum of 2-[2,2-di(1*H*-indol-3-yl) ethyl] aniline (**5**) in $CDCl_3$.

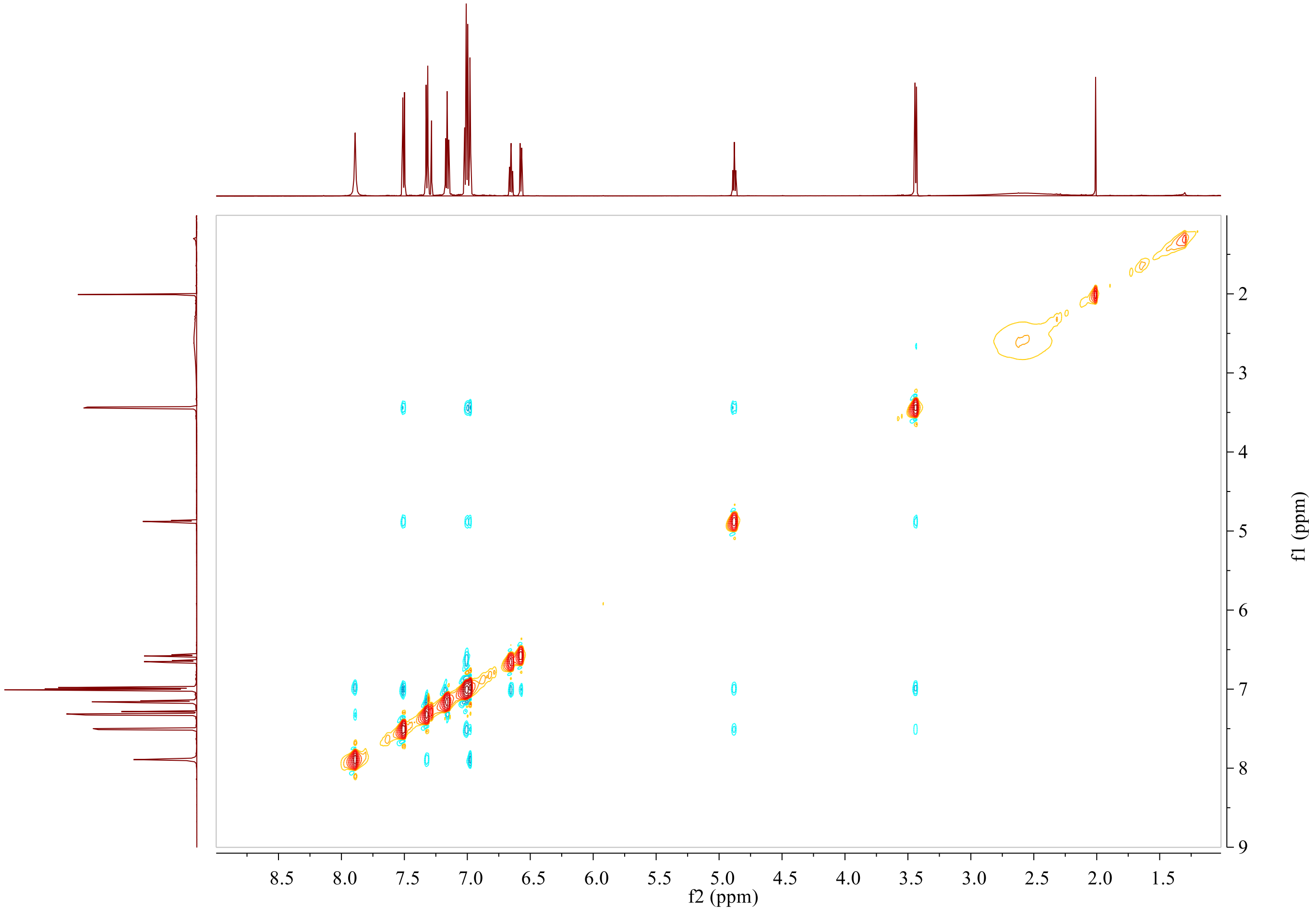

Figure S44. NOESY spectrum of2-[2,2-di(1*H*-indol-3-yl) ethyl] aniline (**5**) in $CDCl_3$.

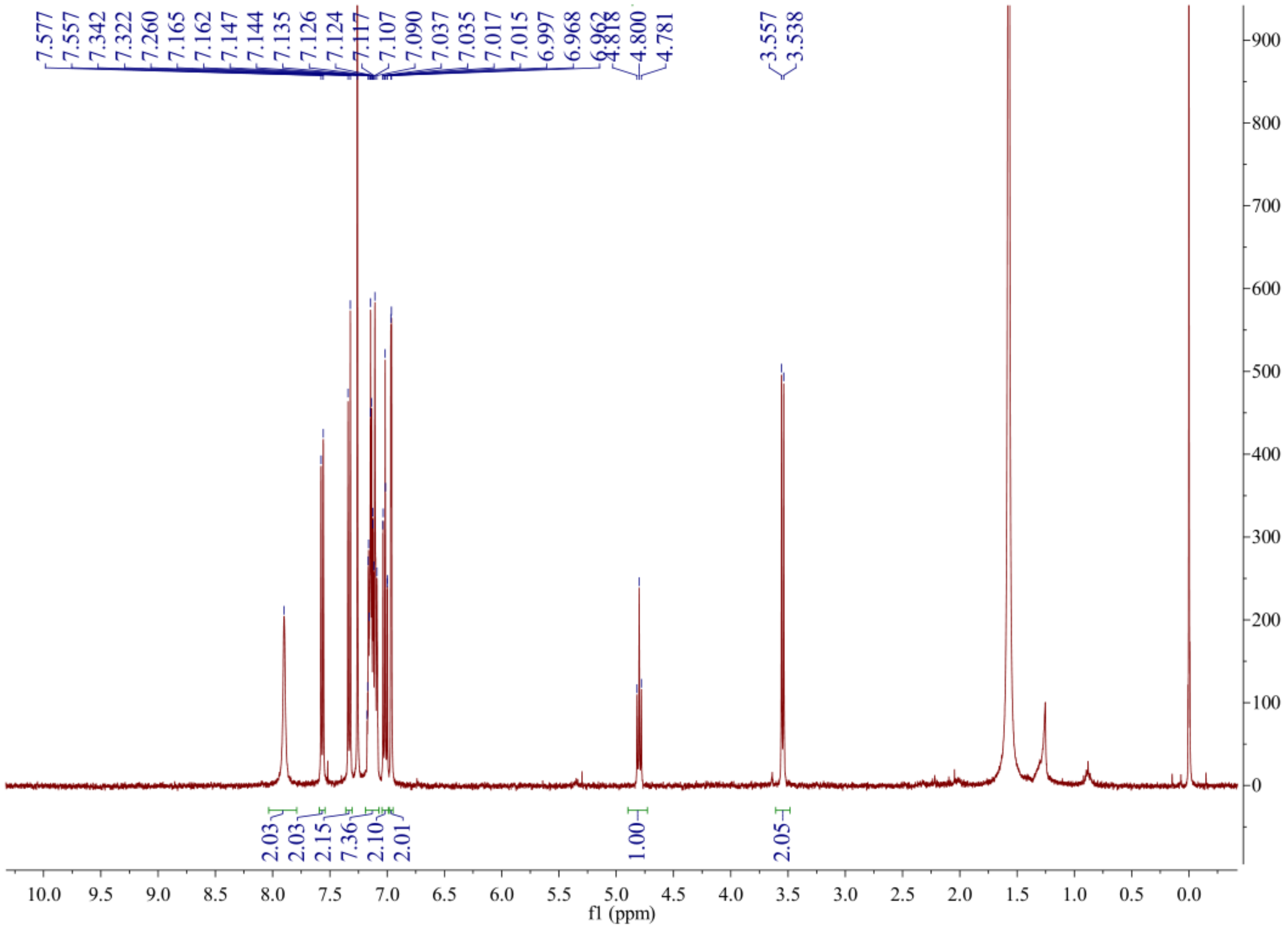

Figure S45. ^{1}H NMR spectrum of 3,3'-Diindolyl(phenyl)methane (**6**) in $CDCl_3$ (400 MHz).

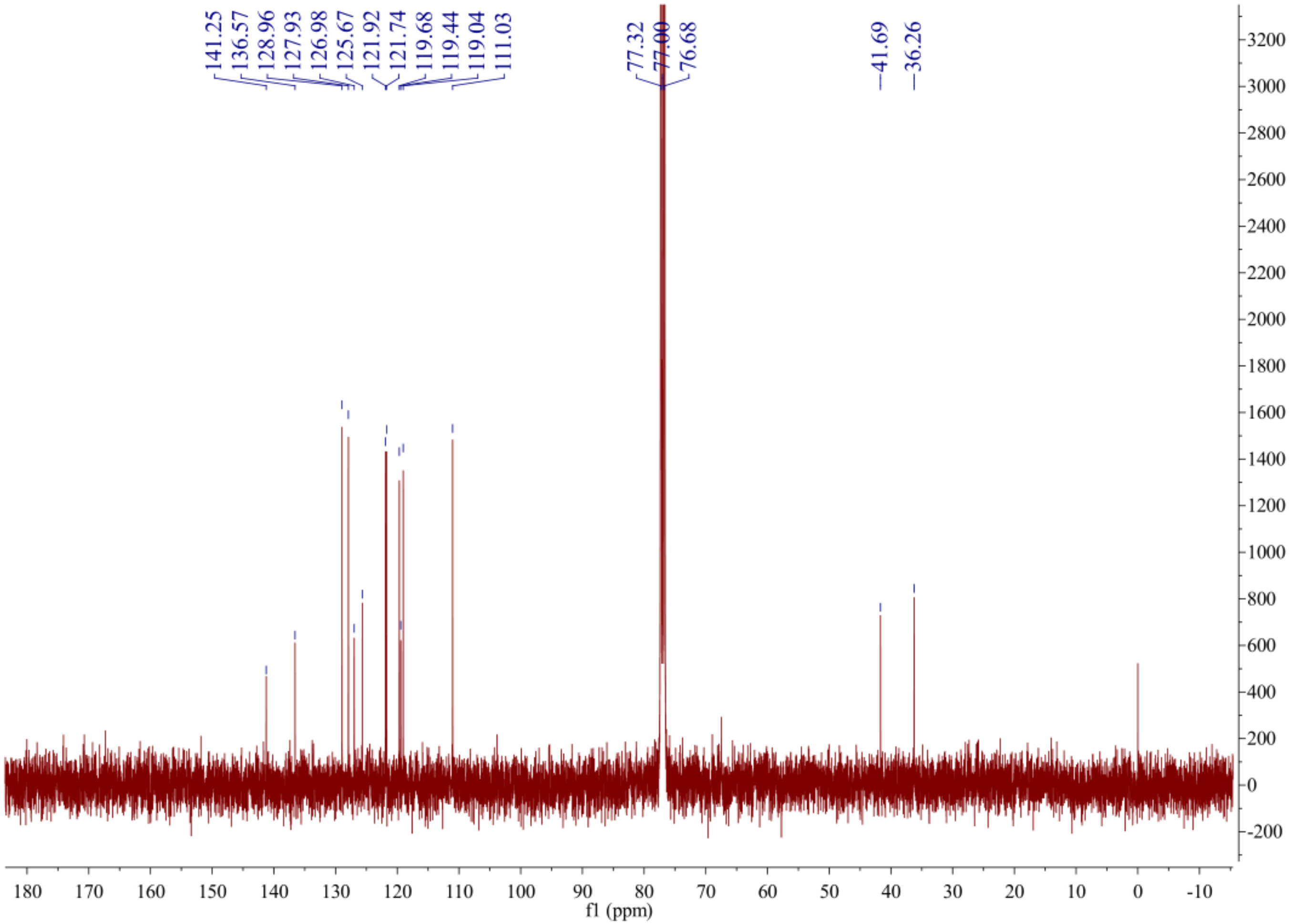

Figure S46. ^{13}C NMR spectrum of 3,3'-Diindolyl(phenyl)methane (**6**) in $CDCl_3$ (100 MHz).

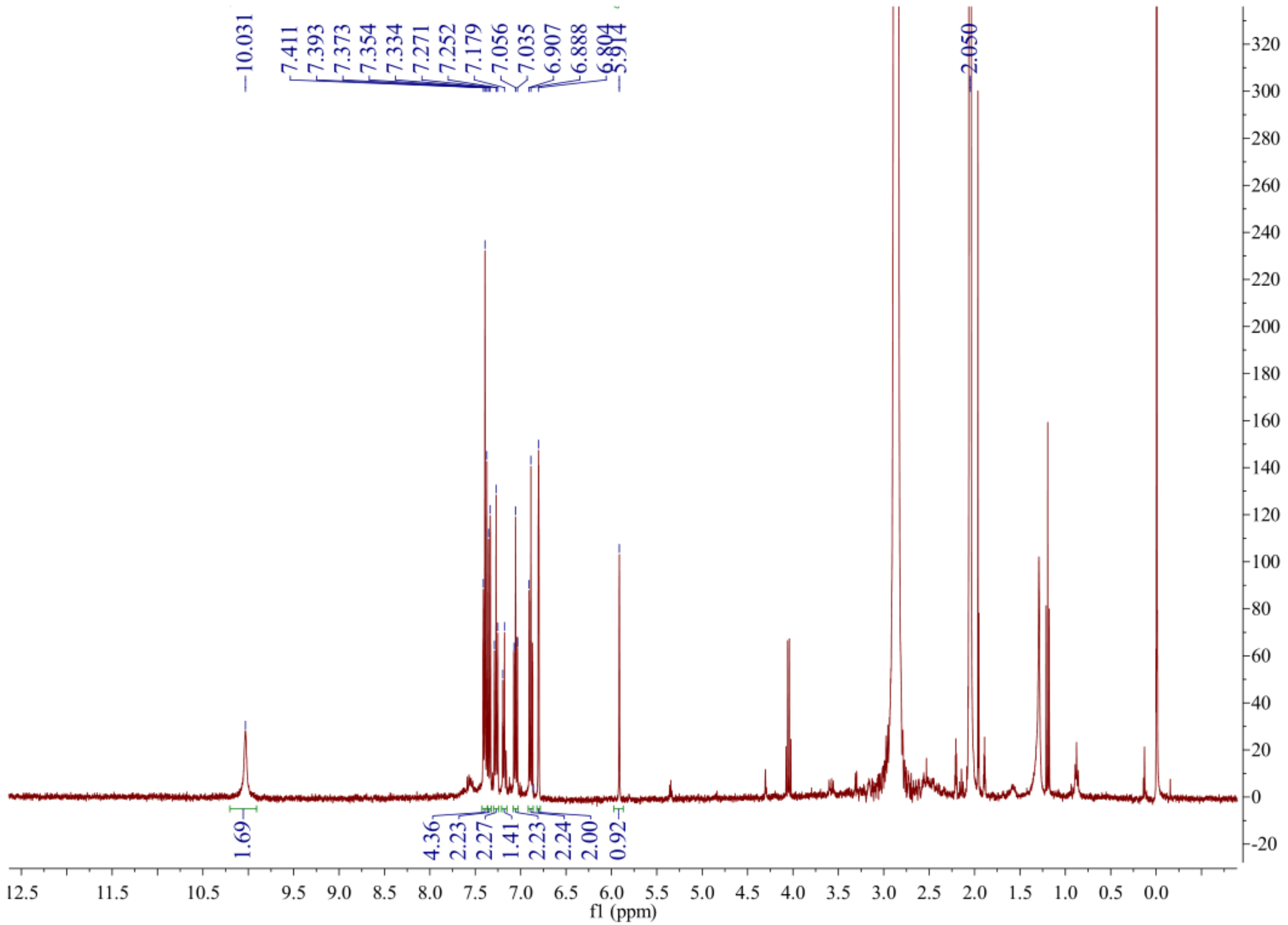

Figure S47. ^{1}H NMR spectrum of 1,1-(3,3'-Diindolyl)-2-phenylethane (**7**) in acetone-d_6 (400 MHz).

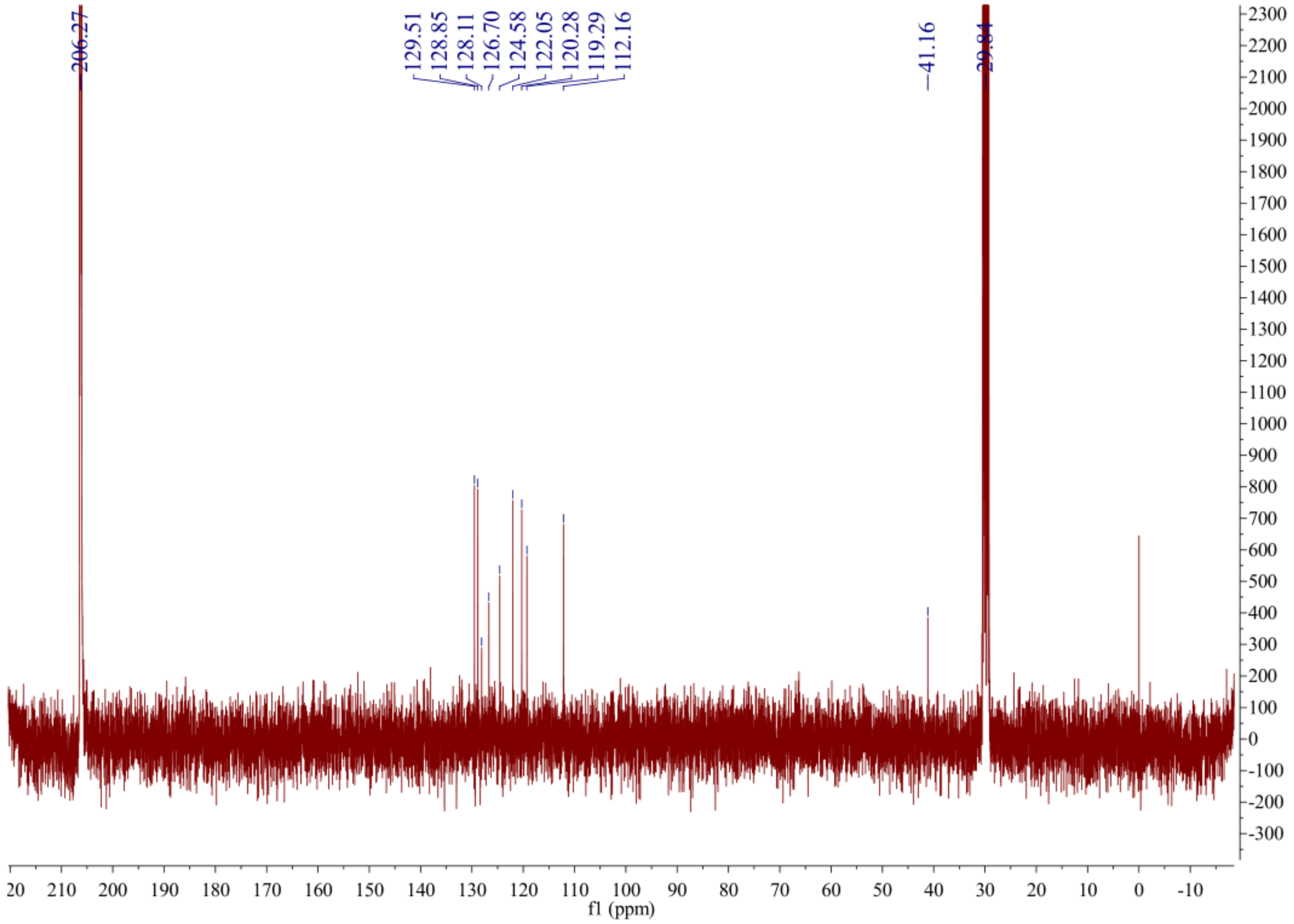

Figure S48. ^{13}C NMR spectrum of 1,1-(3,3'-Diindolyl)-2-phenylethane (**7**) in acetone-d_6 (100 MHz).

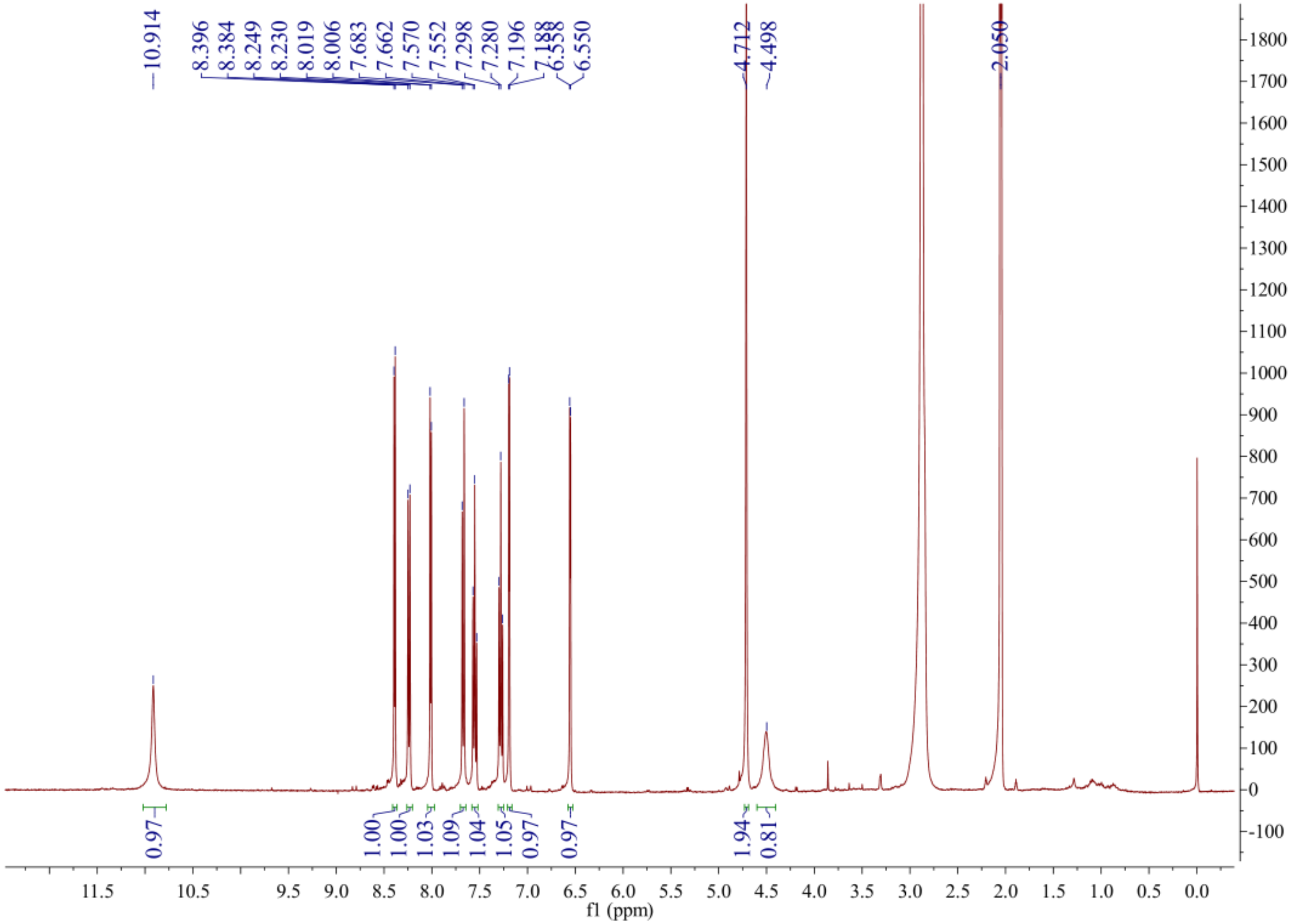

Figure S49. ^{1}H NMR spectrum of perlolyrin (**8**) in acetone-d_6 (400 MHz).

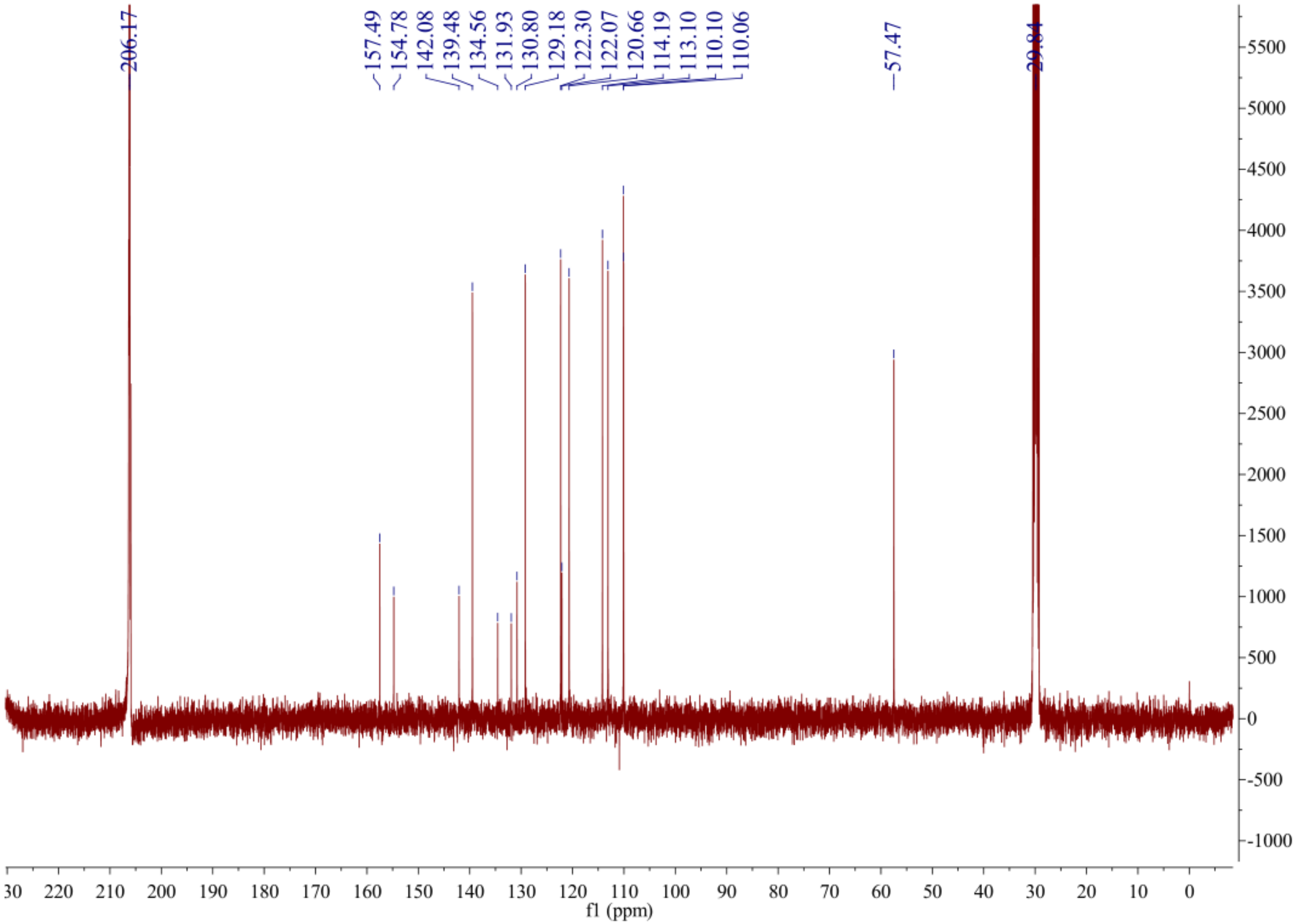

Figure S50. ^{13}C NMR spectrum of perlolyrin (**8**) in acetone-d_6 (100 MHz).

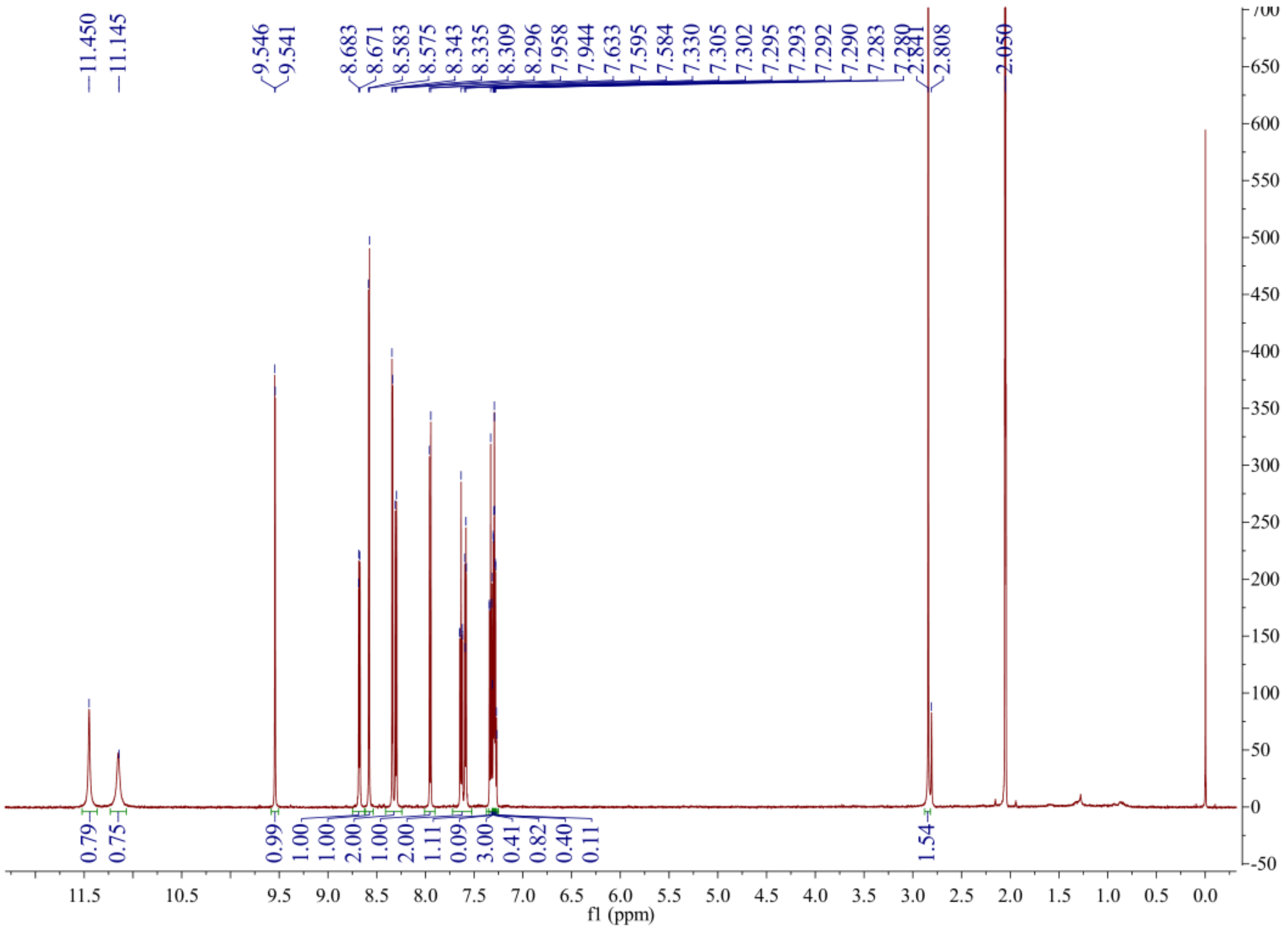

Figure S51. ^{1}H NMR spectrum of pityriacitrin (**9**) in acetone-d_6 (600 MHz).

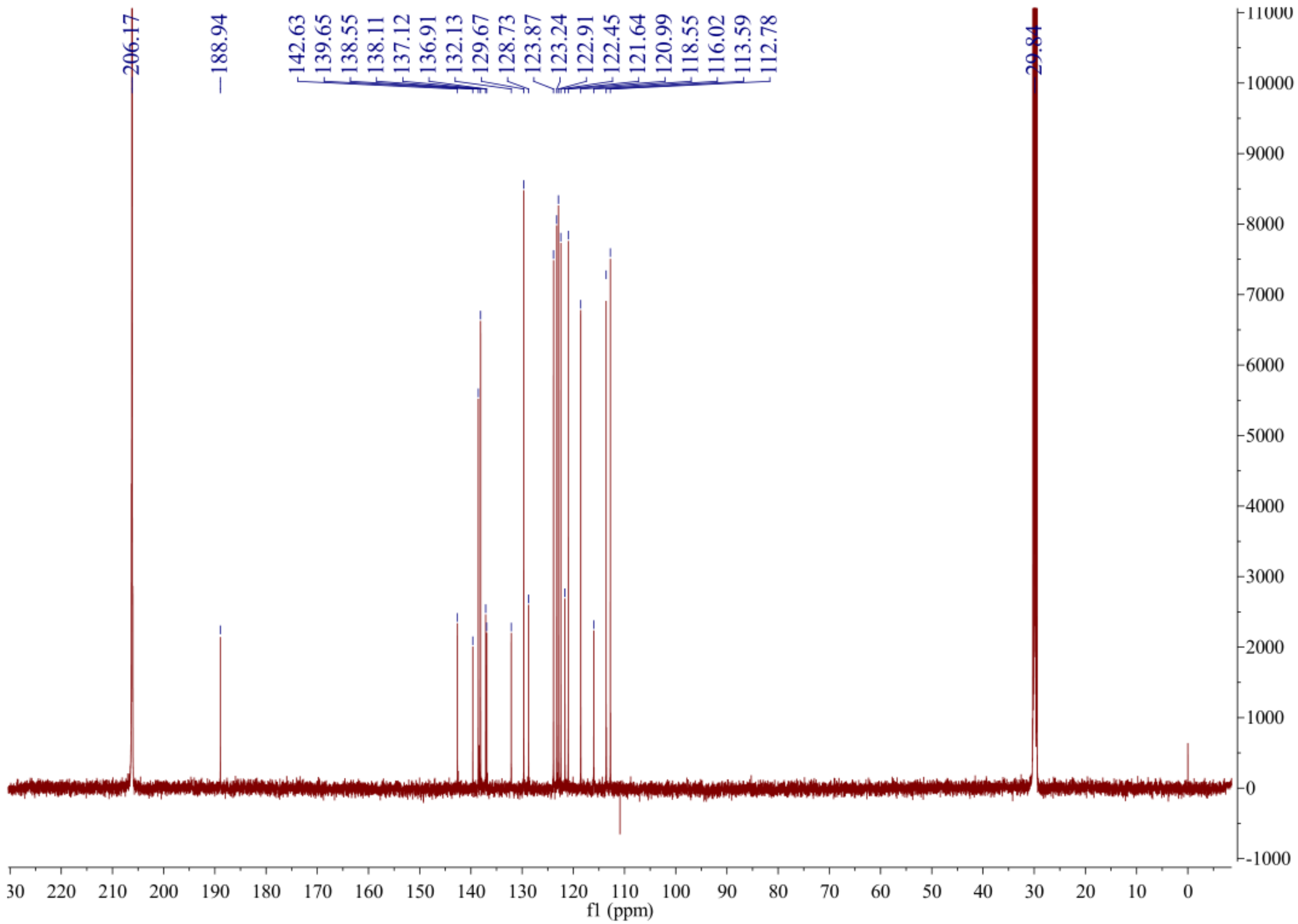

Figure S52. ^{13}C NMR spectrum of pityriacitrin (**9**) in acetone-d_6 (150 MHz).

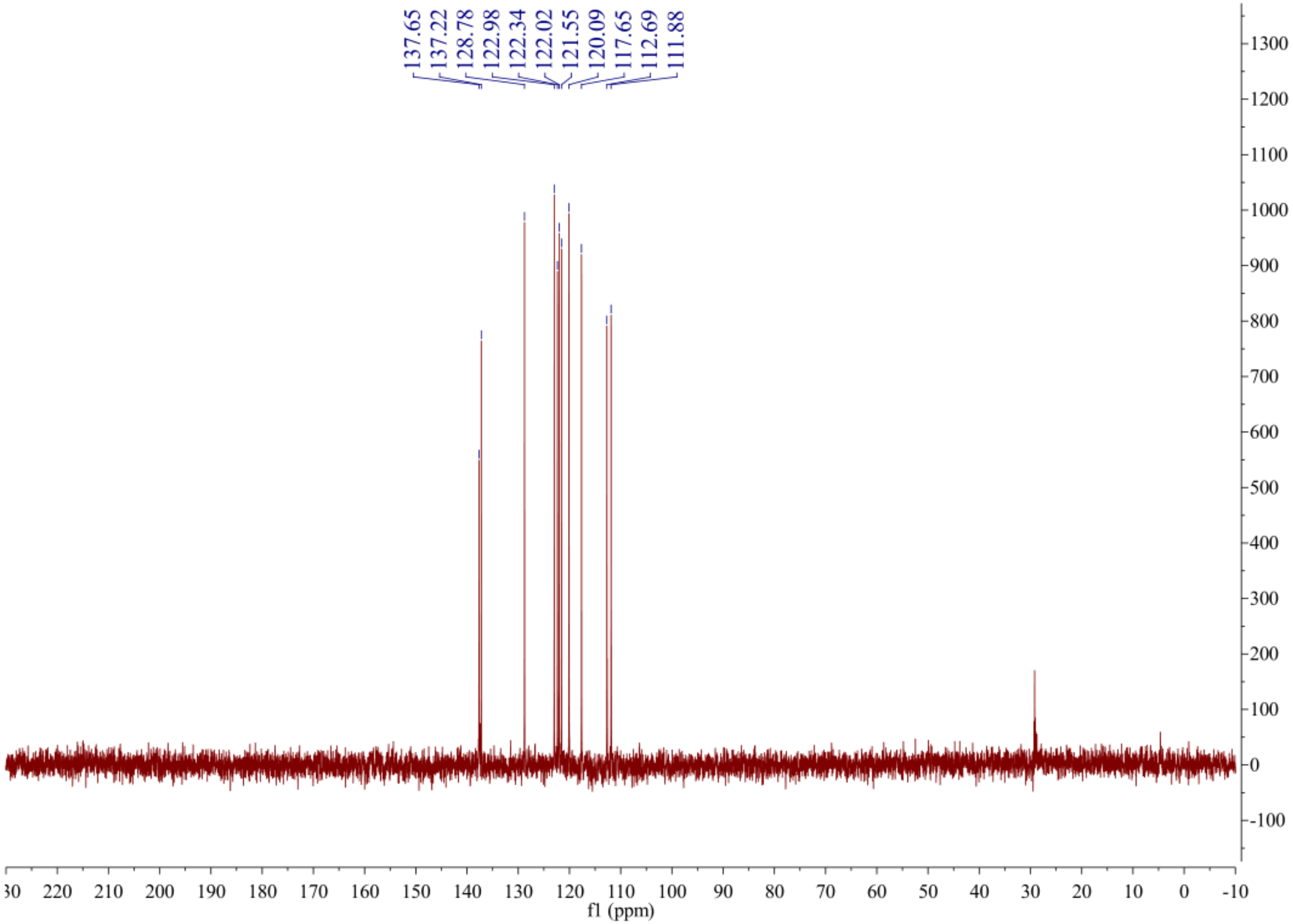

Figure S53. DEPT 135 spectrum of pityriacitrin (**9**) in acetone-d_6 (150 MHz).

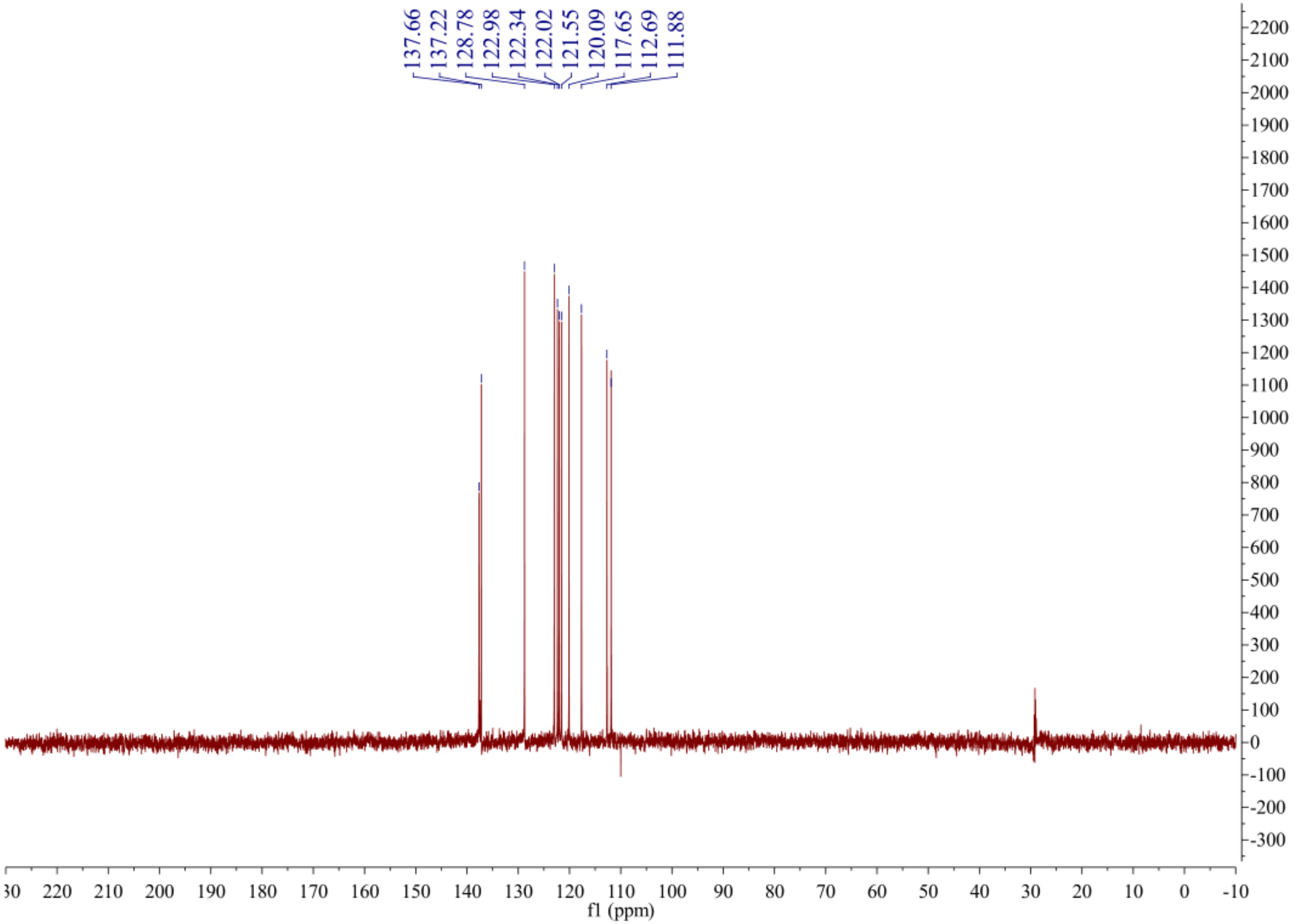

Figure S54. DEPT 90 spectrum of pityriacitrin (**9**) in acetone-d_6 (150 MHz).

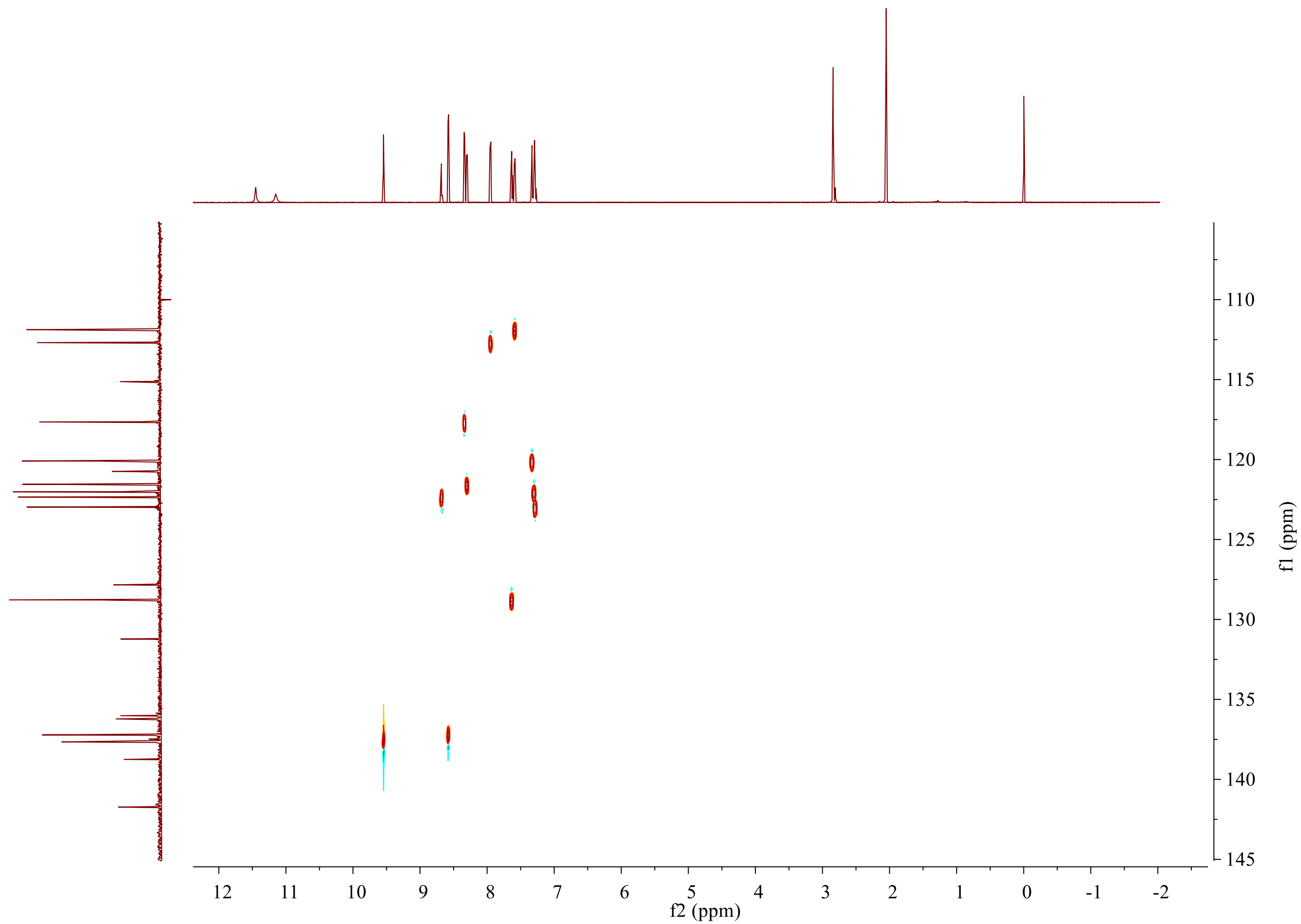

Figure S55. HMQC spectrum of pityriacitrin (**9**) in acetone-d_6.

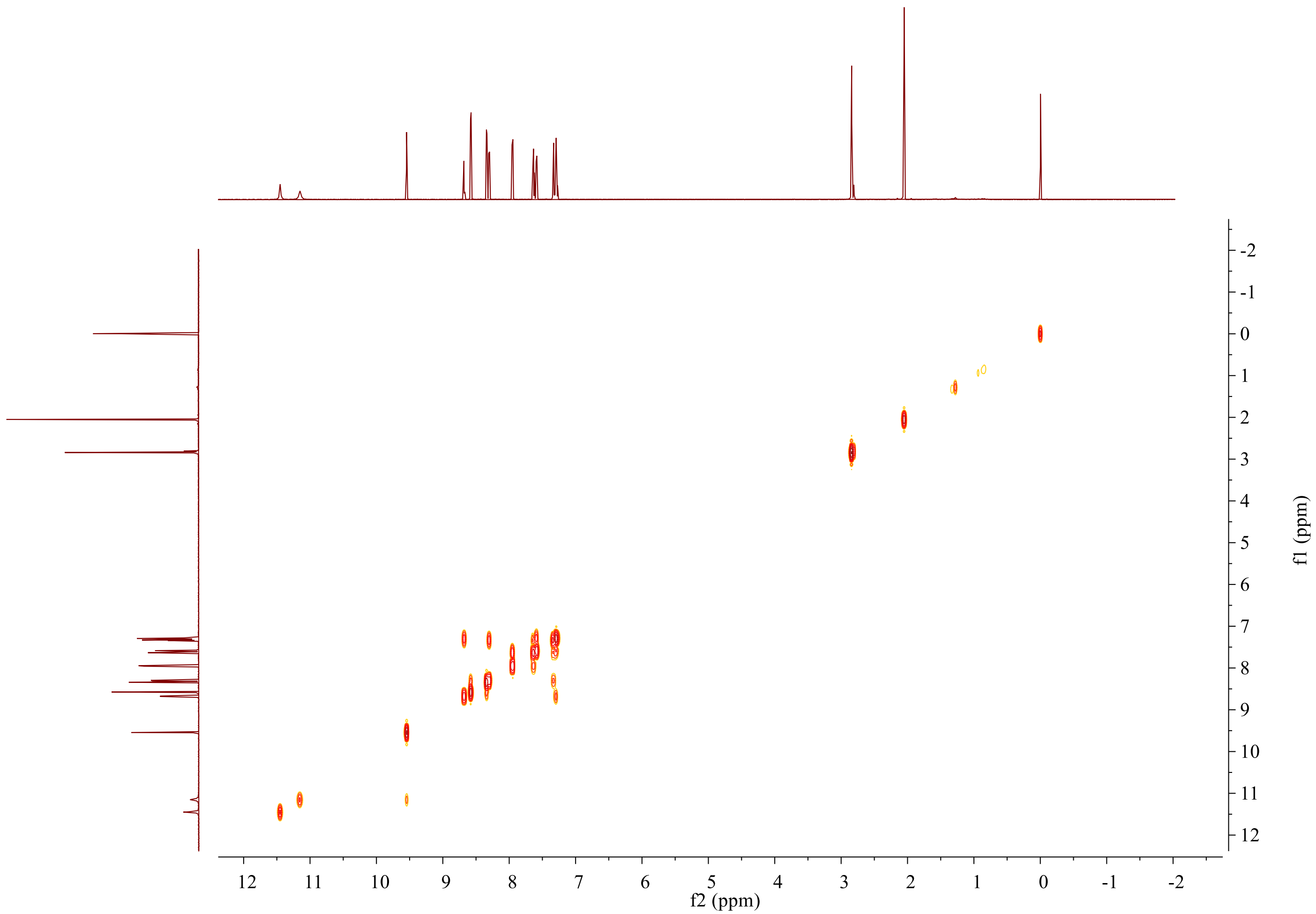

Figure S56. ^{1}H–^{1}H COSY spectrum of pityriacitrin (**9**) in acetone-d_6.

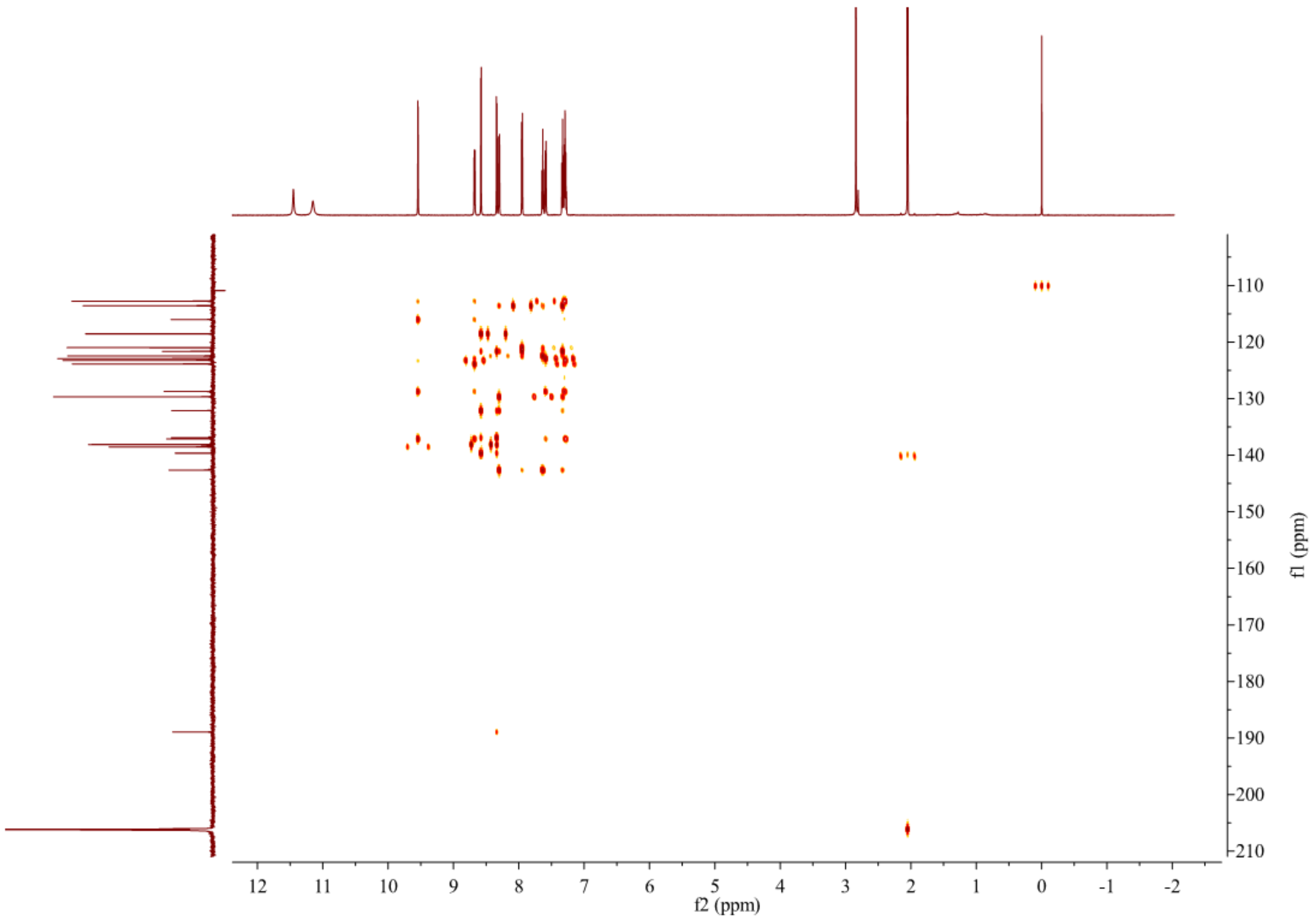

Figure S57. HMBC spectrum of pityriacitrin (**9**) in acetone-d_6.

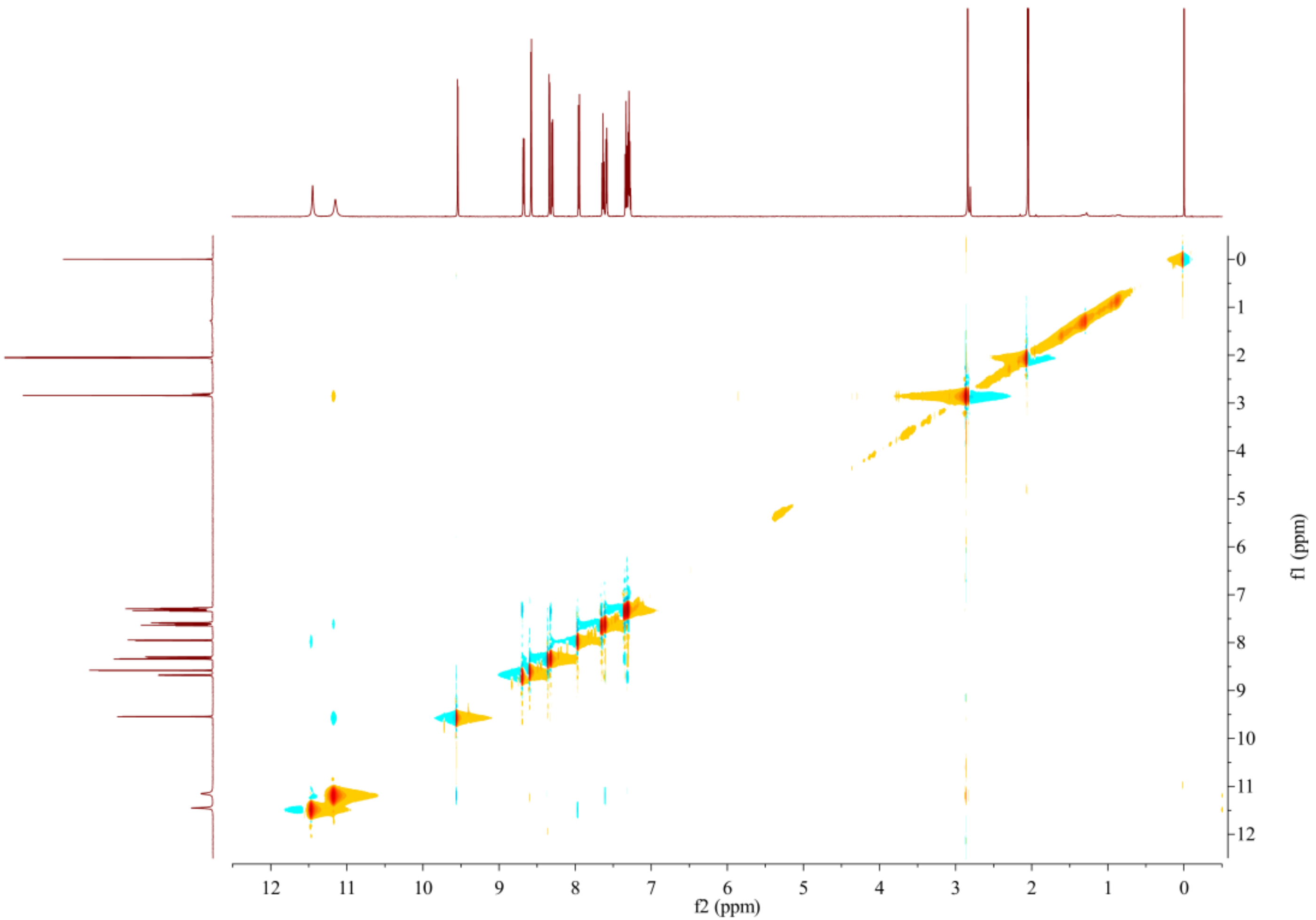

Figure S58. NOESY spectrum of pityriacitrin (**9**) in acetone-d_6.

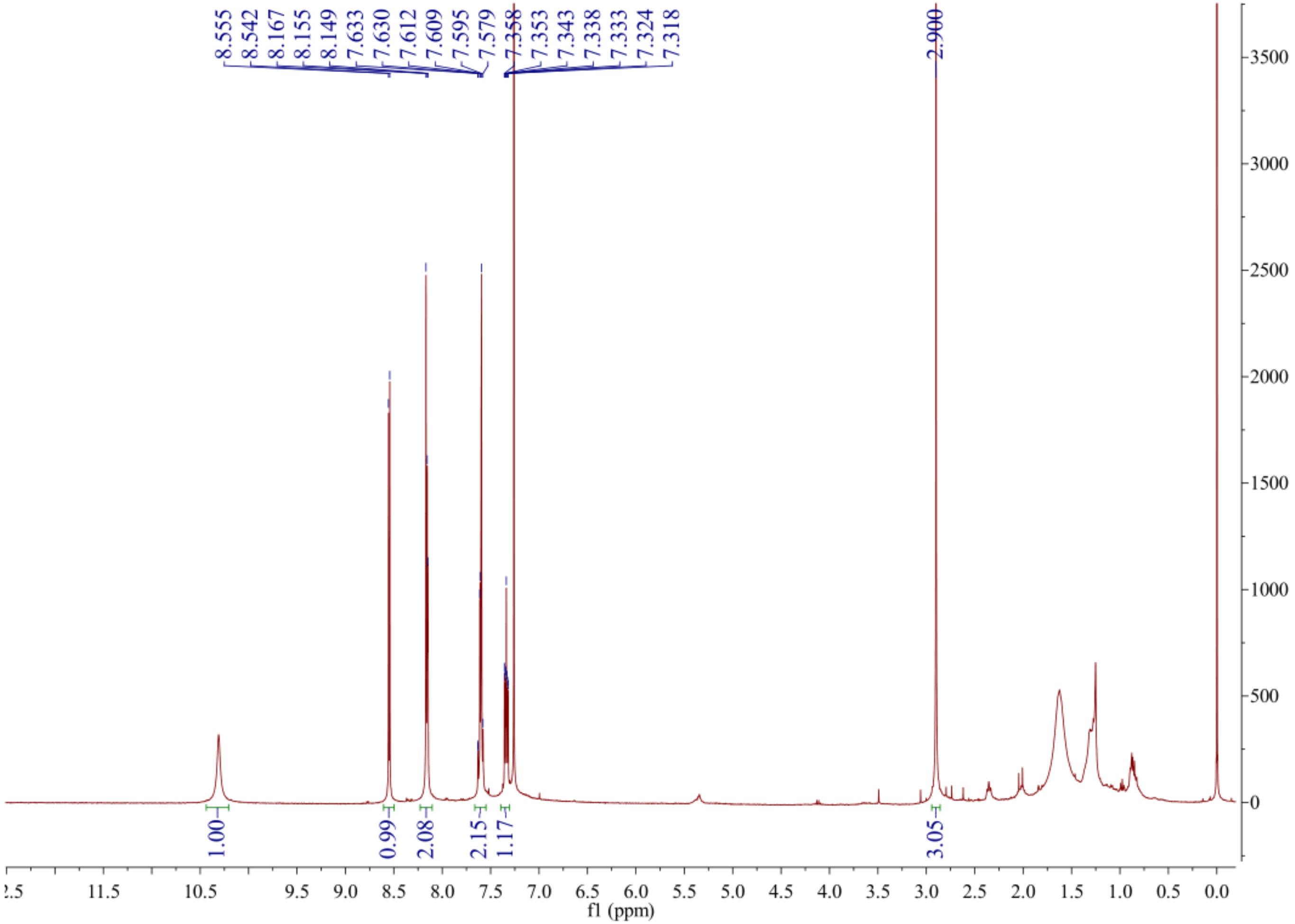

Figure S59. ^{1}H NMR spectrum of 1-acetyl-β-carboline (**10**) in $CDCl_3$ (400 MHz).

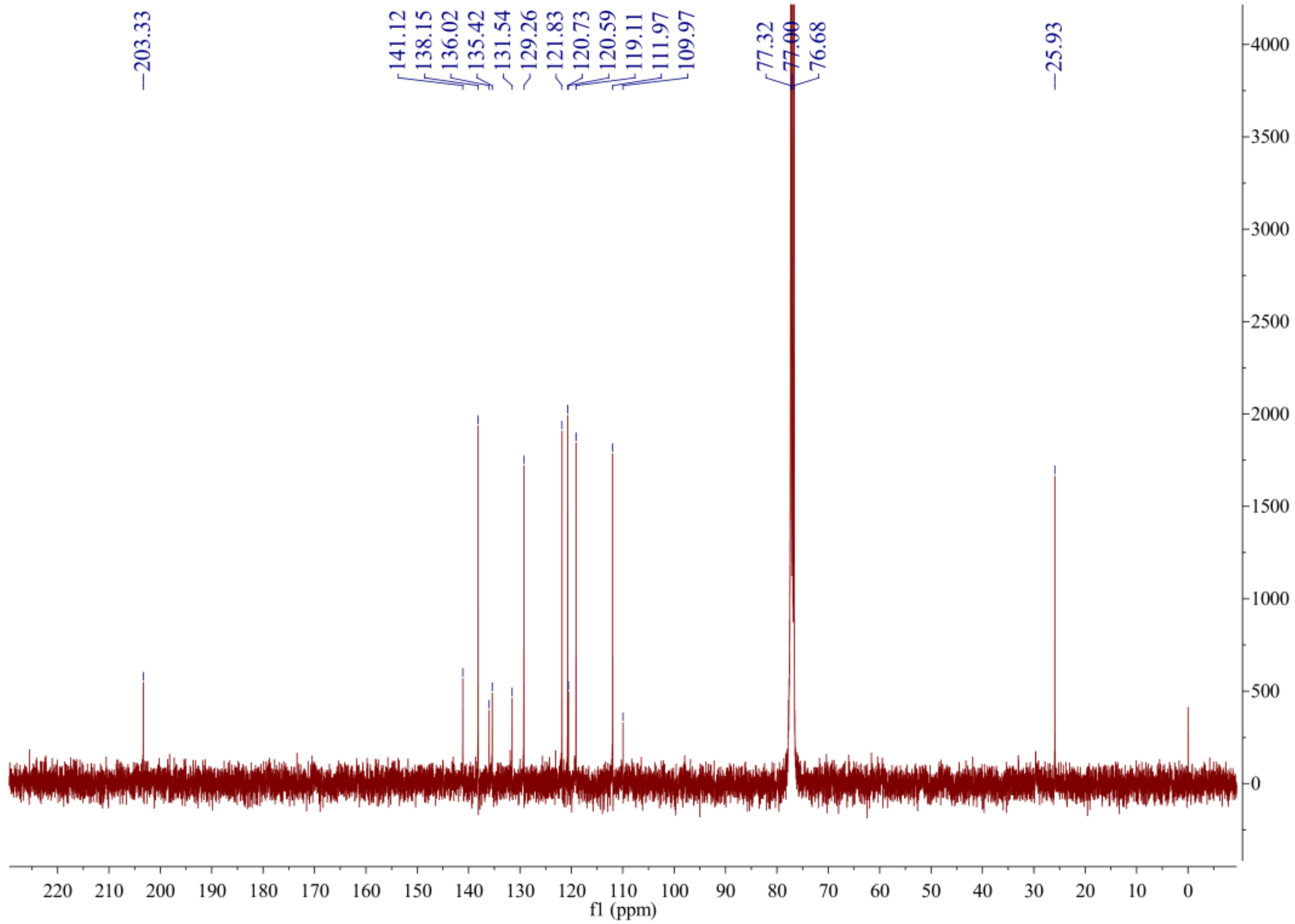

Figure S60. ^{13}C NMR spectrum of 1-acetyl-β-carboline (**10**) in $CDCl_3$ (100 MHz).

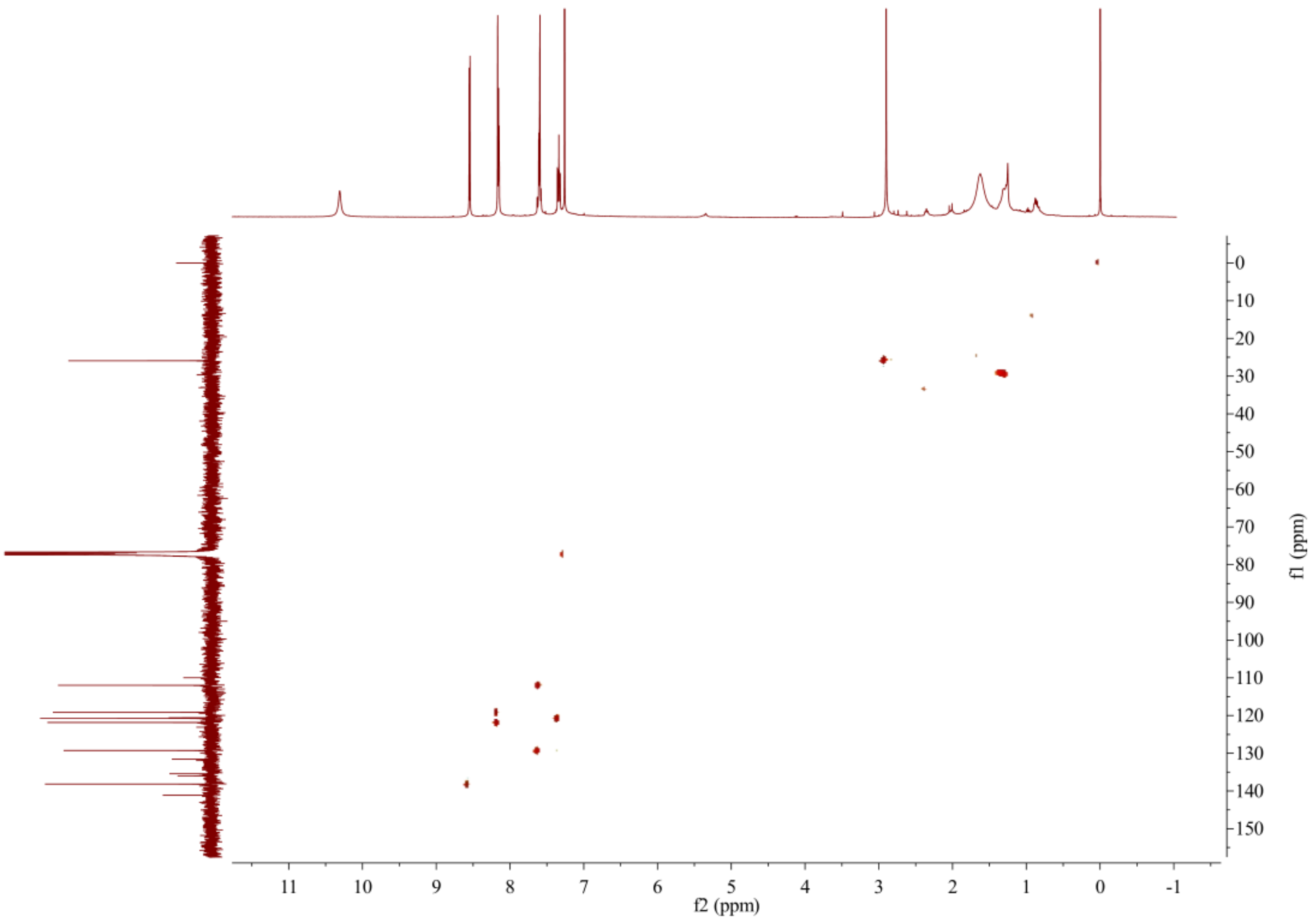

Figure S61. HMQC spectrum of 1-acetyl-β-carboline (**10**) in $CDCl_3$.

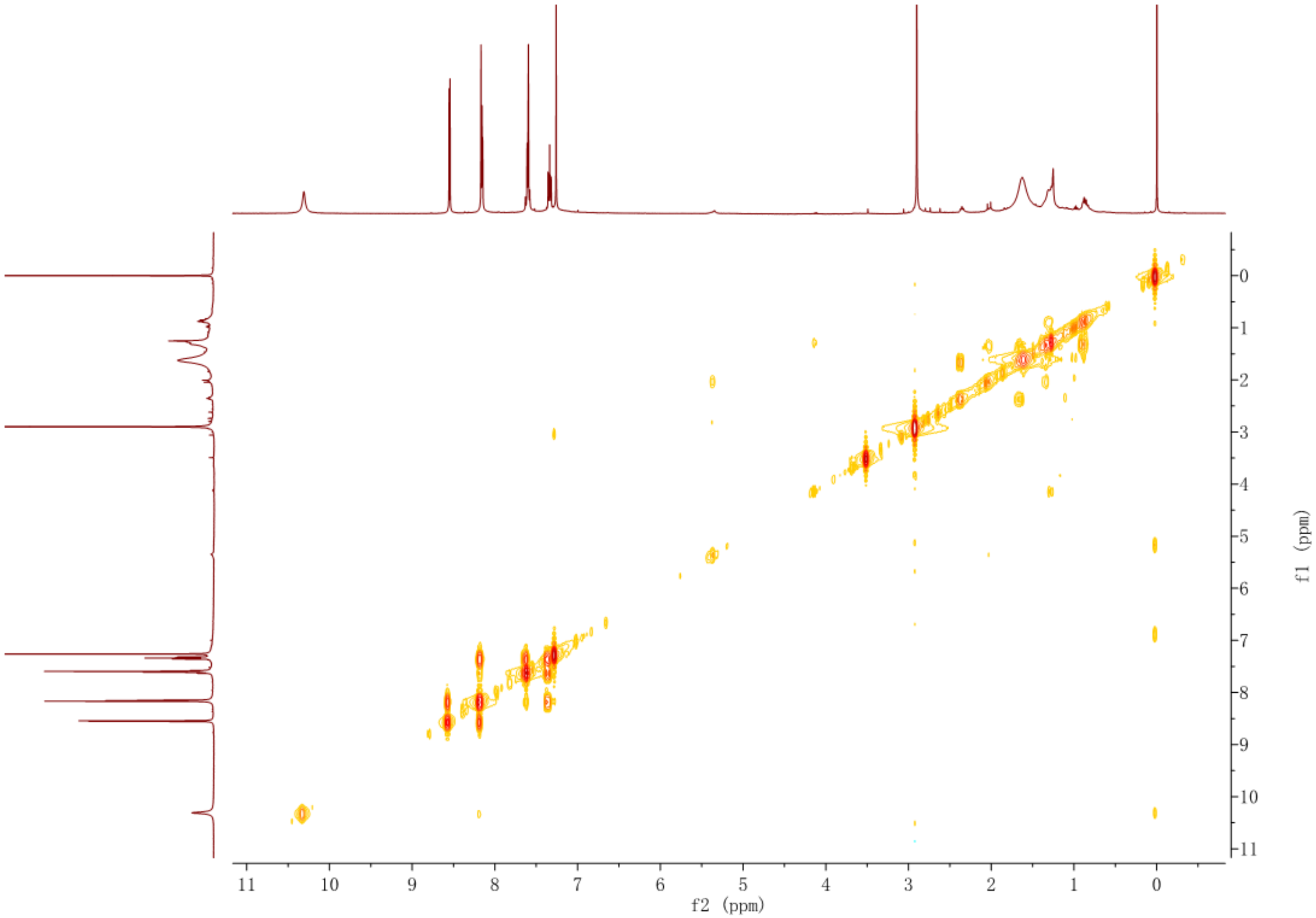

Figure S62. ^1H–^1H COSY spectrum of 1-acetyl-β-carboline (**10**) in $CDCl_3$.

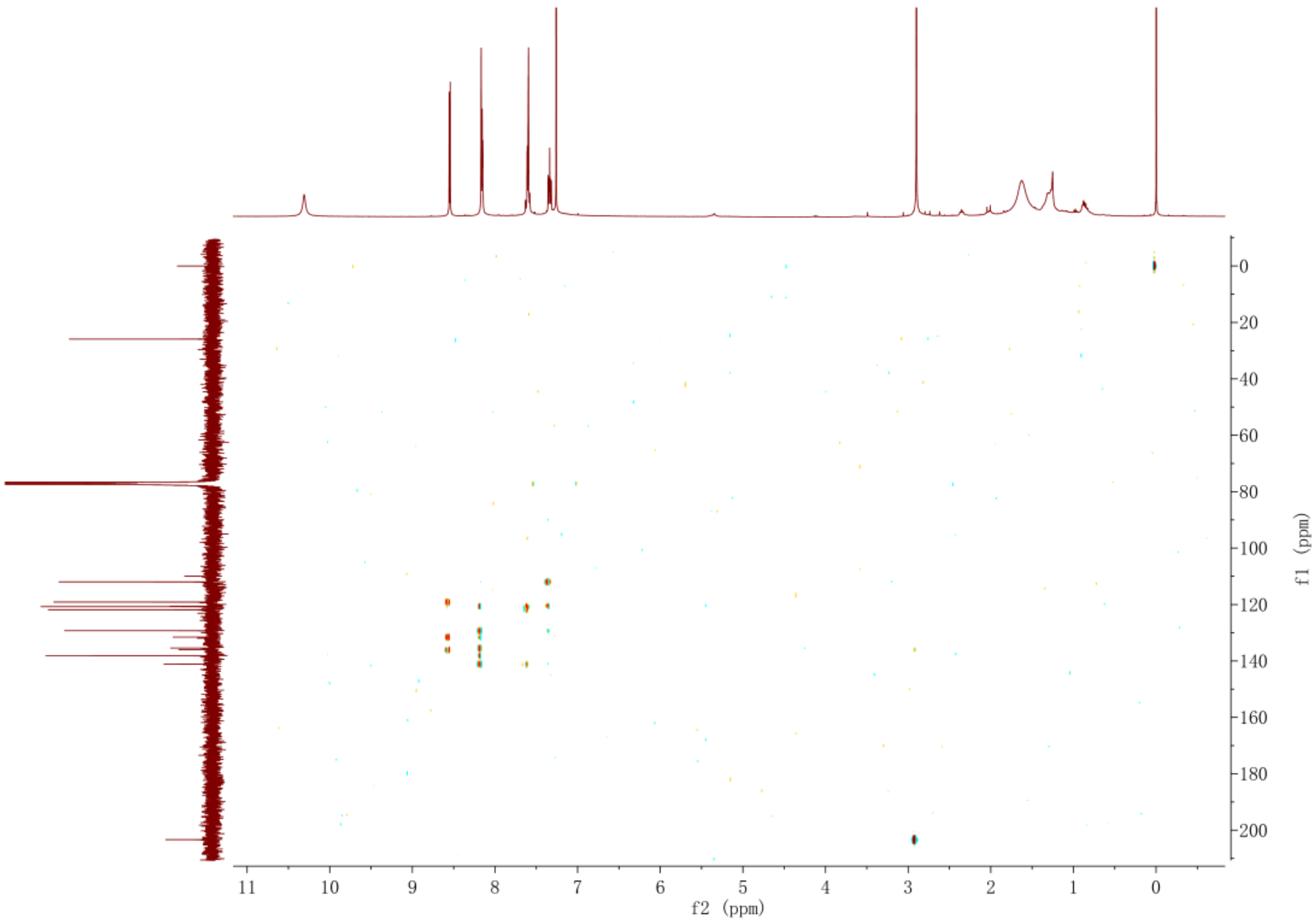

Figure S63. HMBC spectrum of 1-acetyl-β-carboline (**10**) in $CDCl_3$.

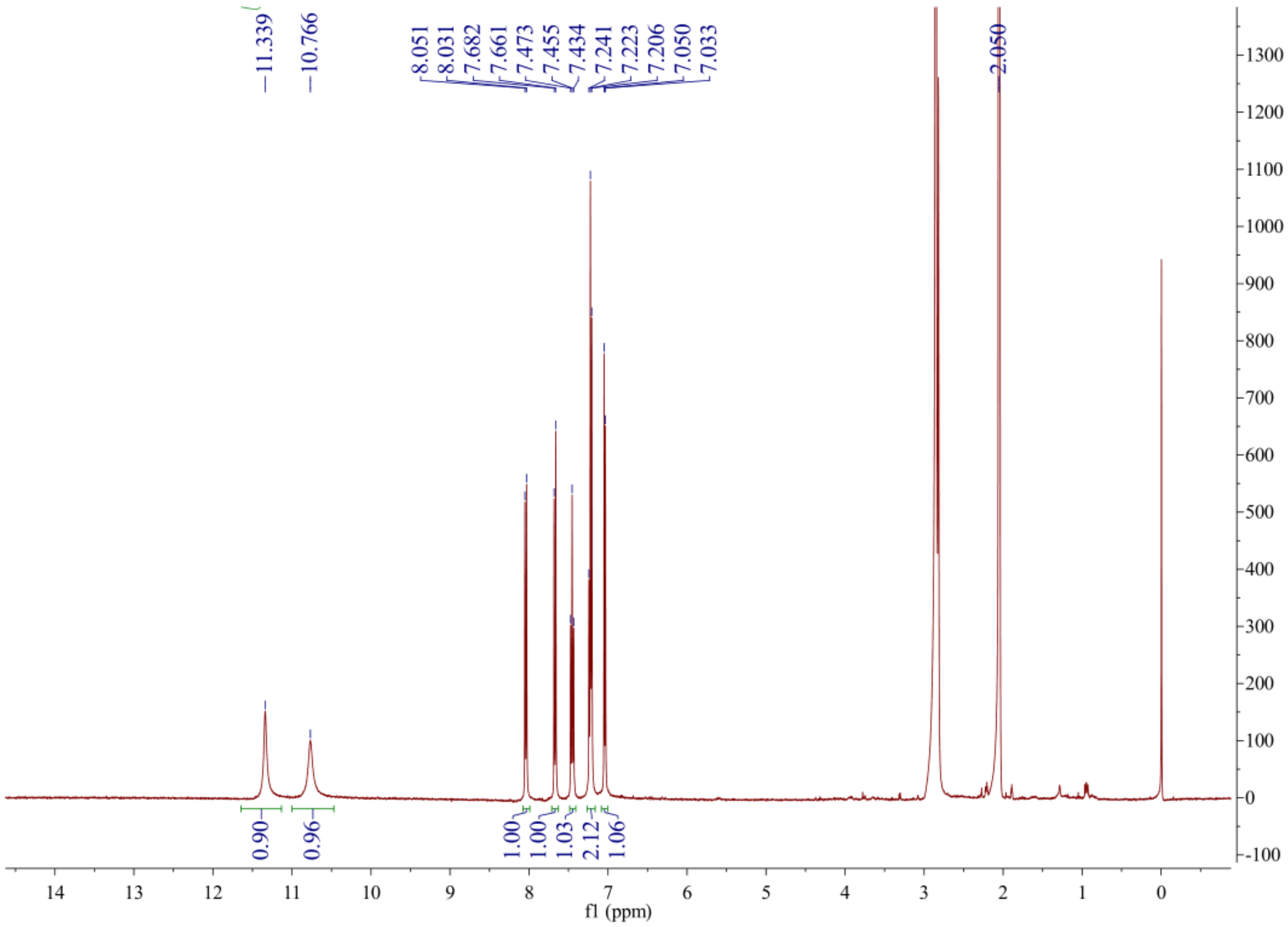

Figure S64. ^{1}H NMR spectrum of 3-hydroxy-β-carboline (**11**) in acetone-d_6 (400 MHz).

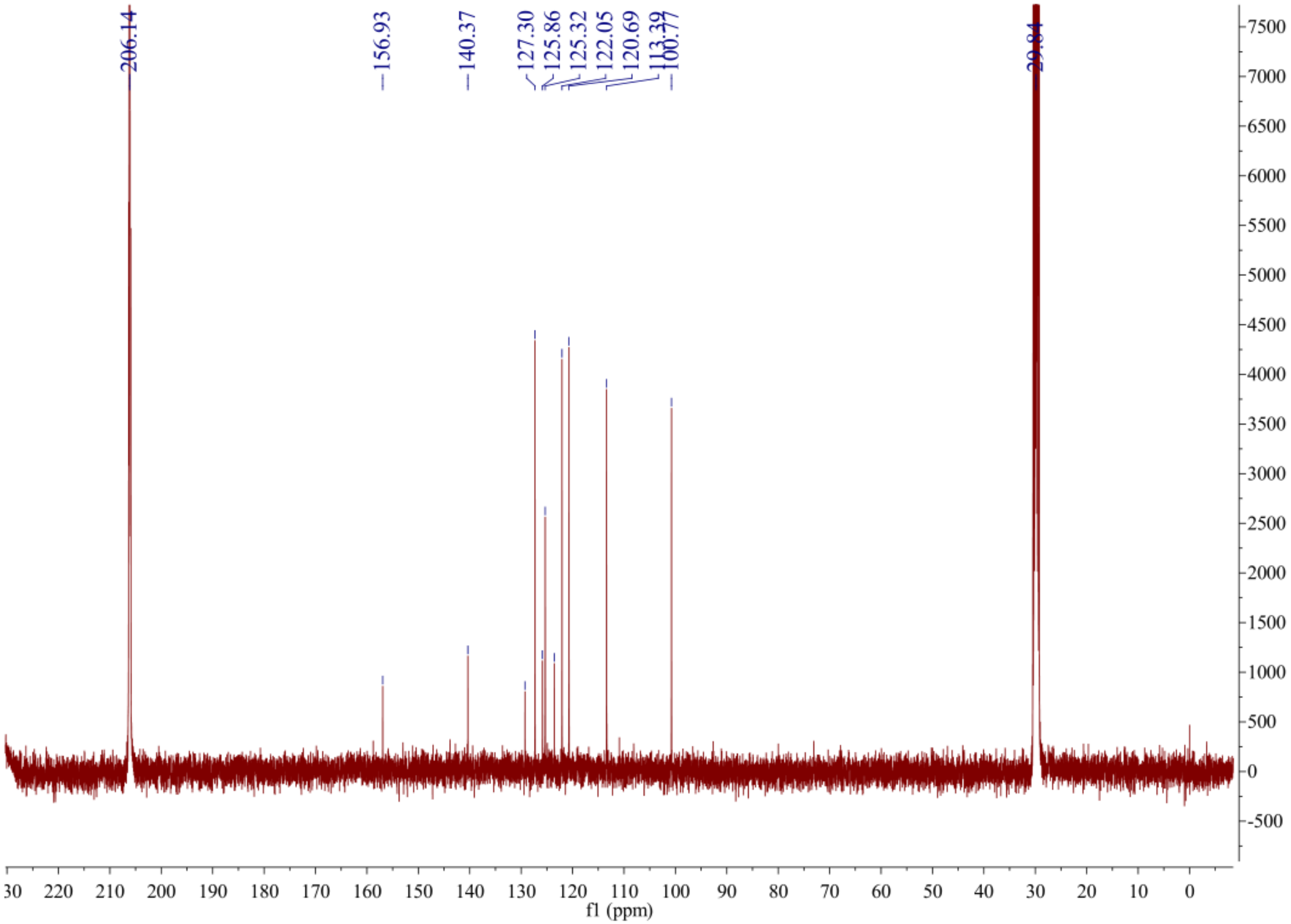

Figure S65. ^{13}C NMR spectrum of 3-hydroxy-β-carboline (**11**) in acetone-d_6 (100 MHz).

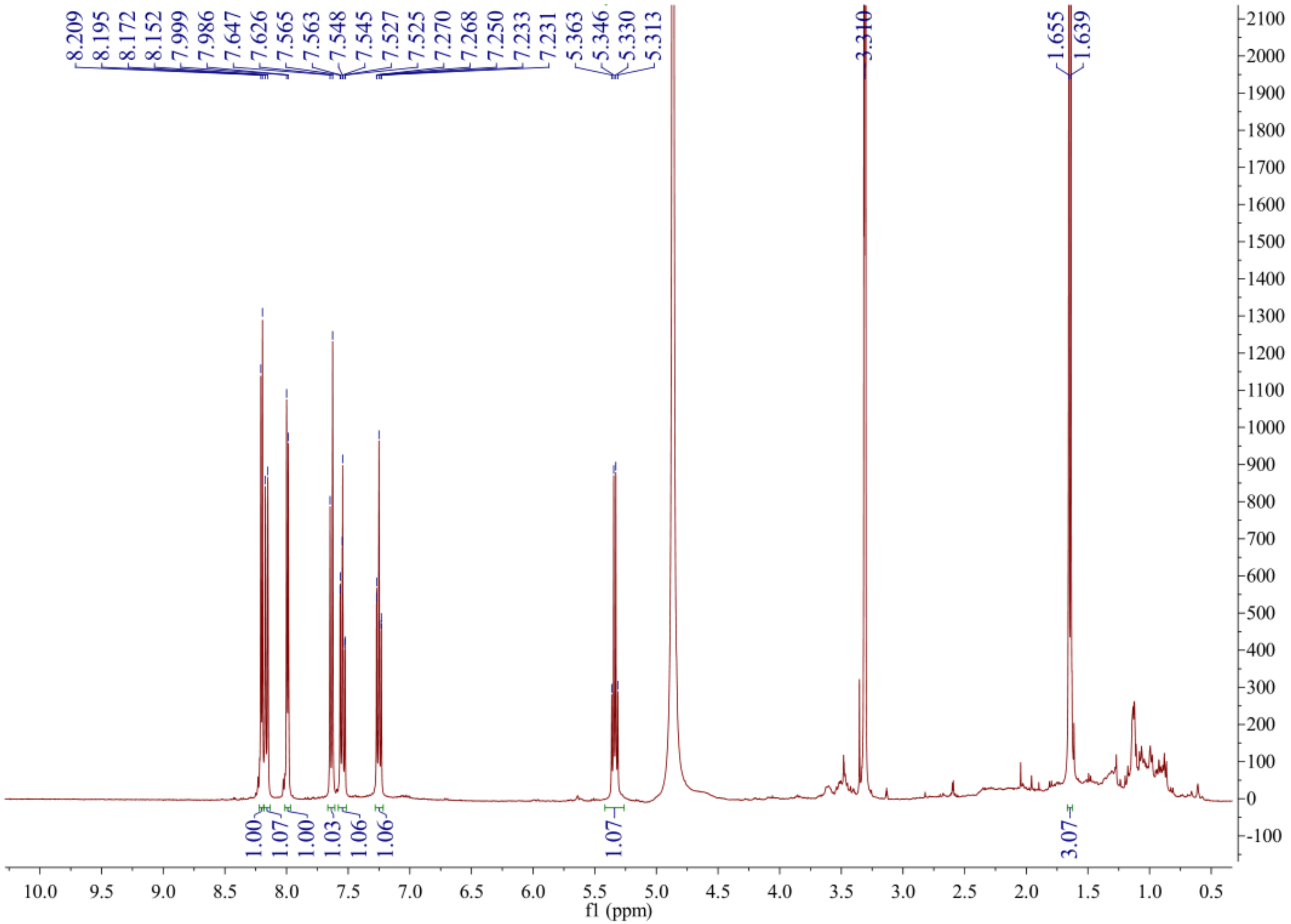

Figure S66. ^{1}H NMR spectrum of 1-(9*H*-pyrido [3,4-b] indol-1-yl) ethan-1-ol (**12**) in methanol-d_4 (400 MHz).

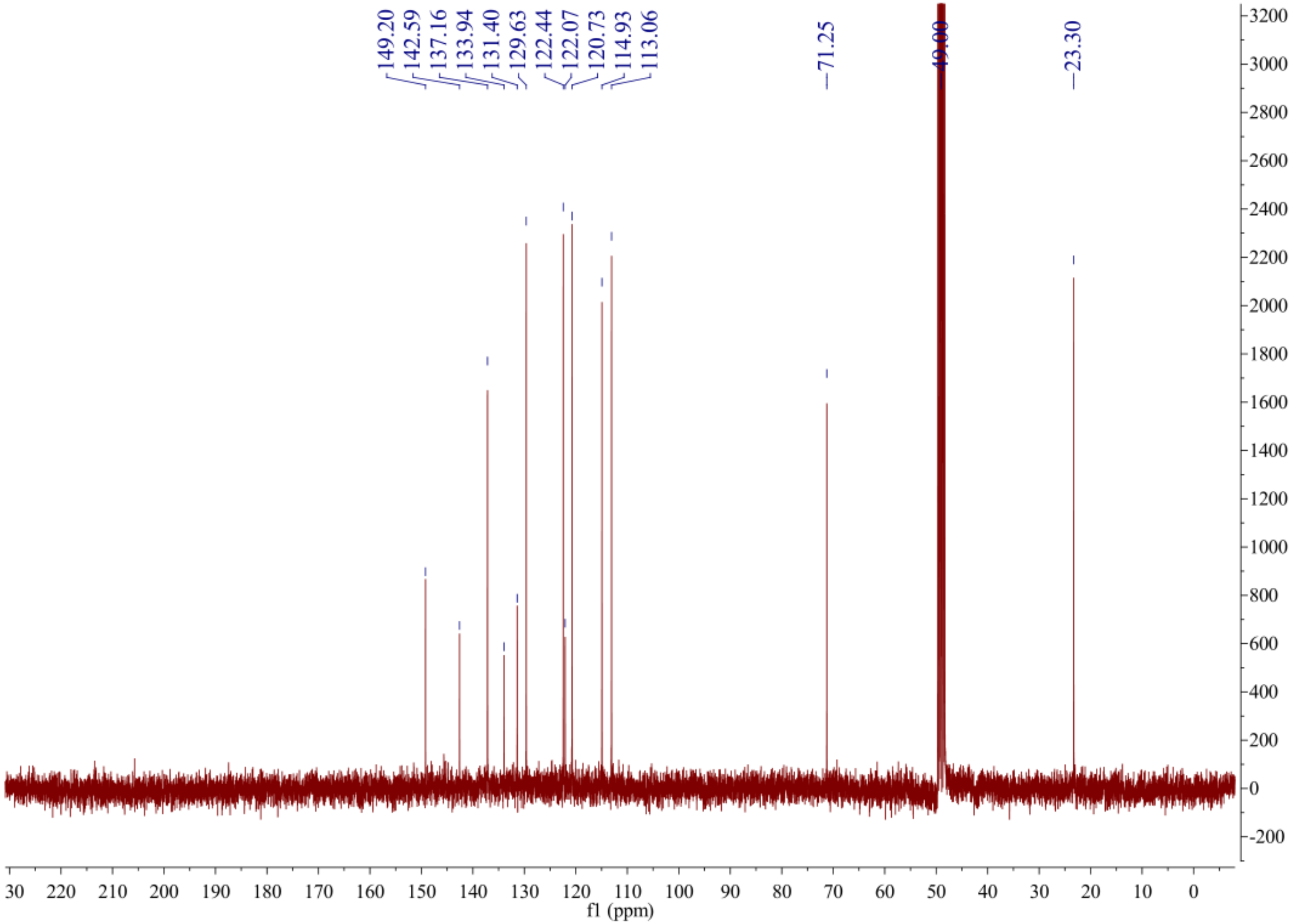

Figure S67. ^{13}C NMR spectrum of 1-(9*H*-pyrido [3,4-b] indol-1-yl) ethan-1-ol (**12**) in methanol-d_4 (100 MHz).

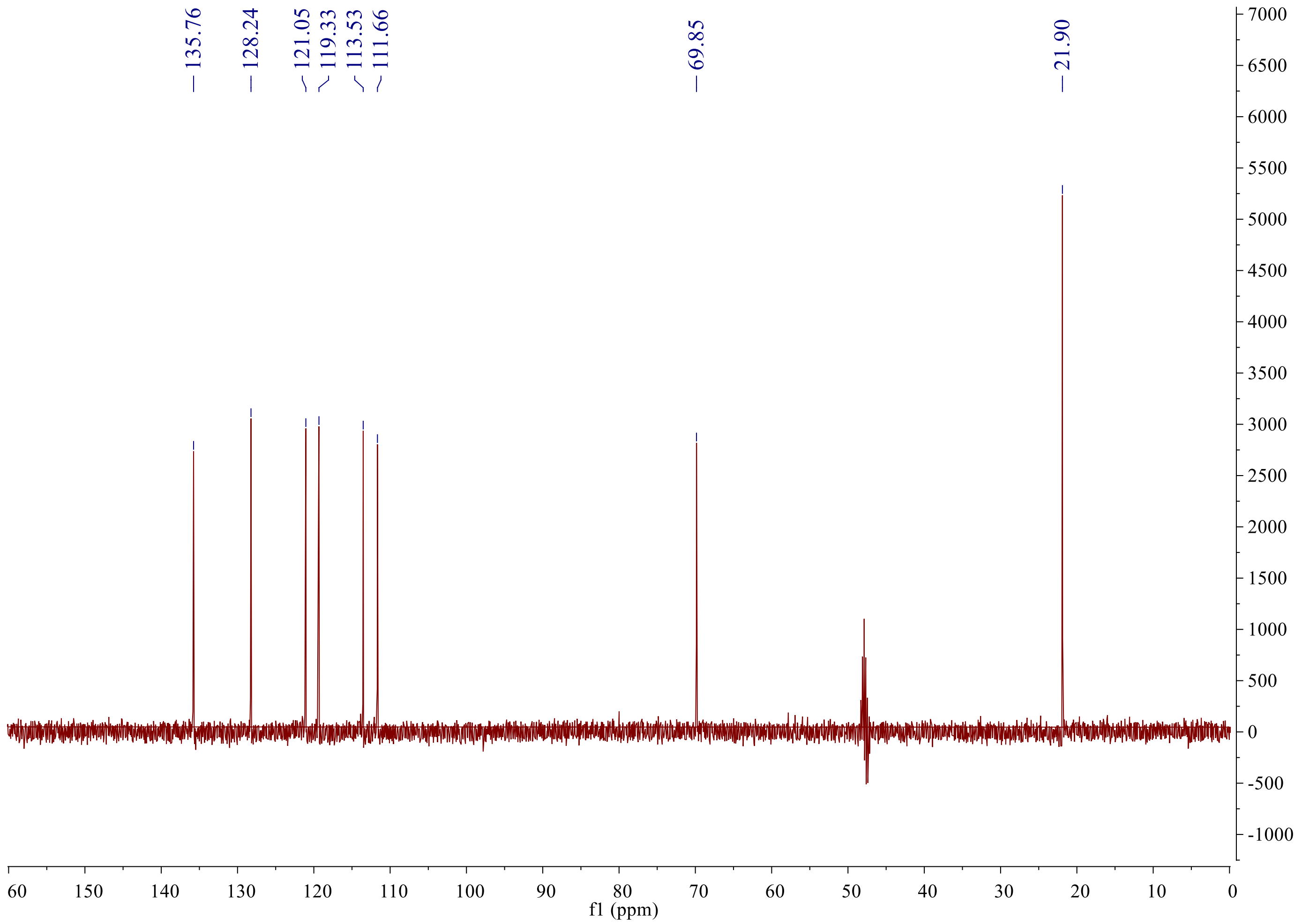

Figure S68. DEPT 135 spectrum of 1-(9*H*-pyrido [3,4-b] indol-1-yl) ethan-1-ol (**12**) in methanol-d_4 (100 MHz).

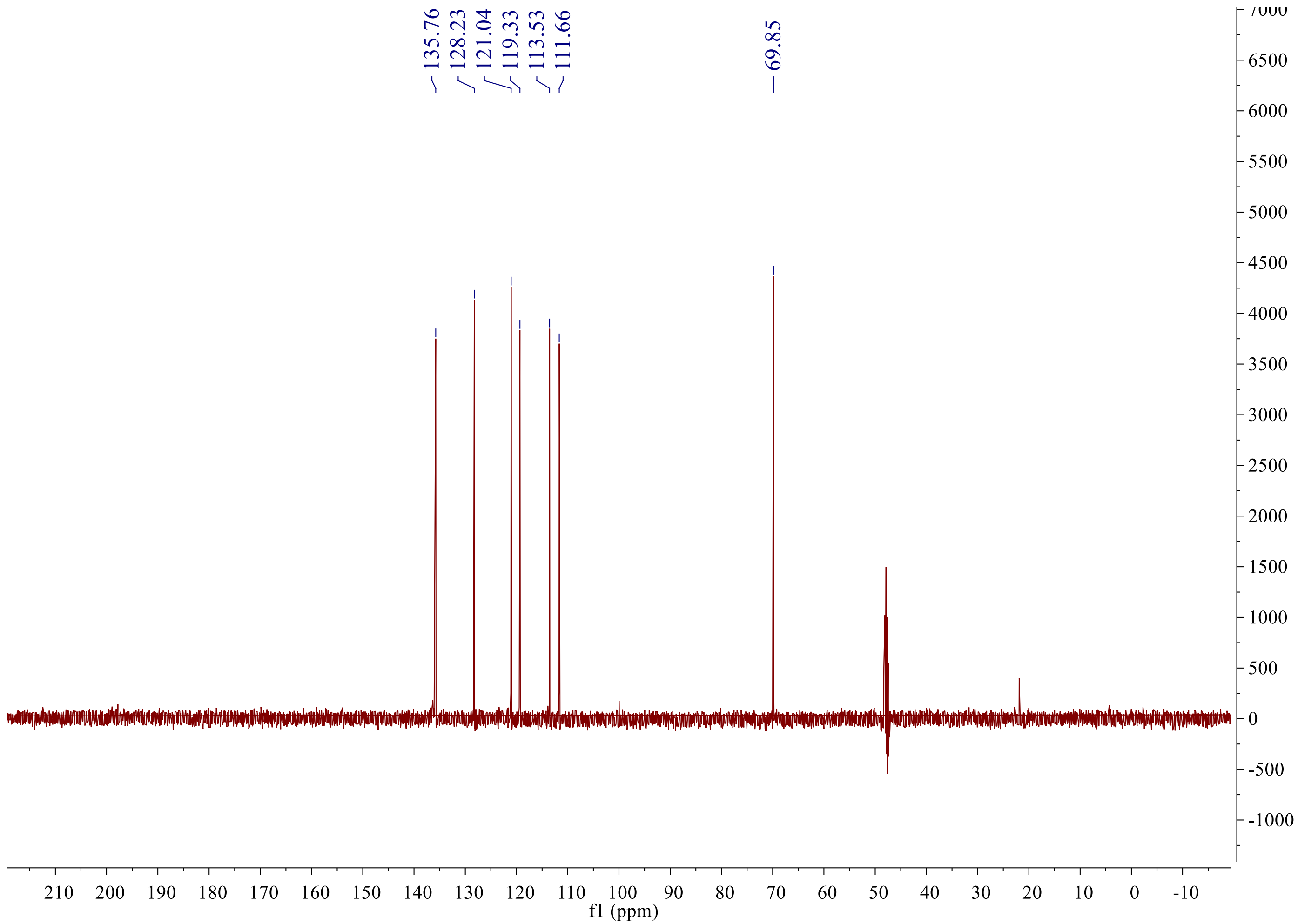

Figure S69. DEPT 90 spectrum of 1-(9*H*-pyrido [3,4-b] indol-1-yl) ethan-1-ol (**12**) in methanol-d_4 (100 MHz).

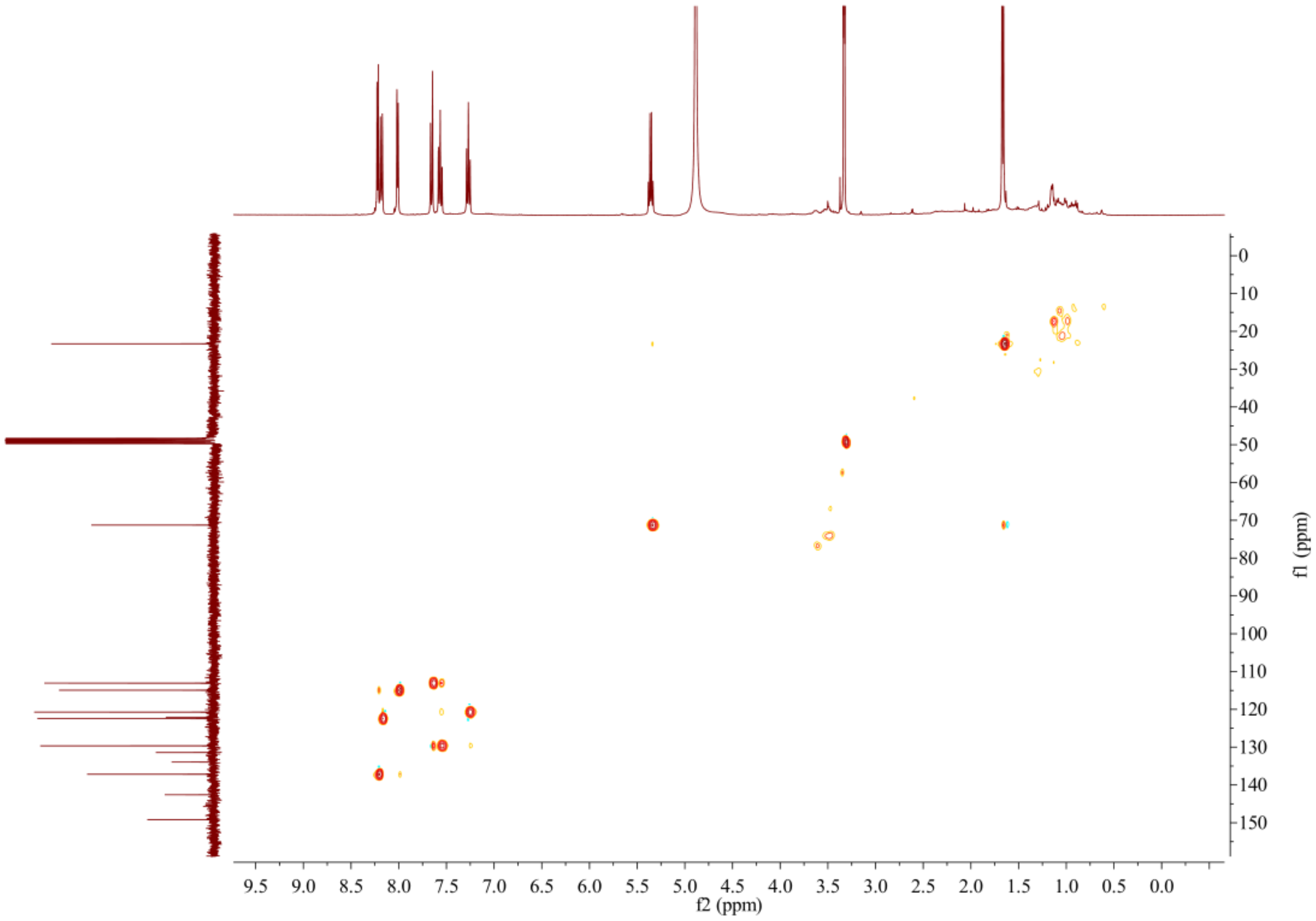

Figure S70. HMQC spectrum of 1-(9*H*-pyrido [3,4-b] indol-1-yl) ethan-1-ol (**12**) in methanol-d_4.

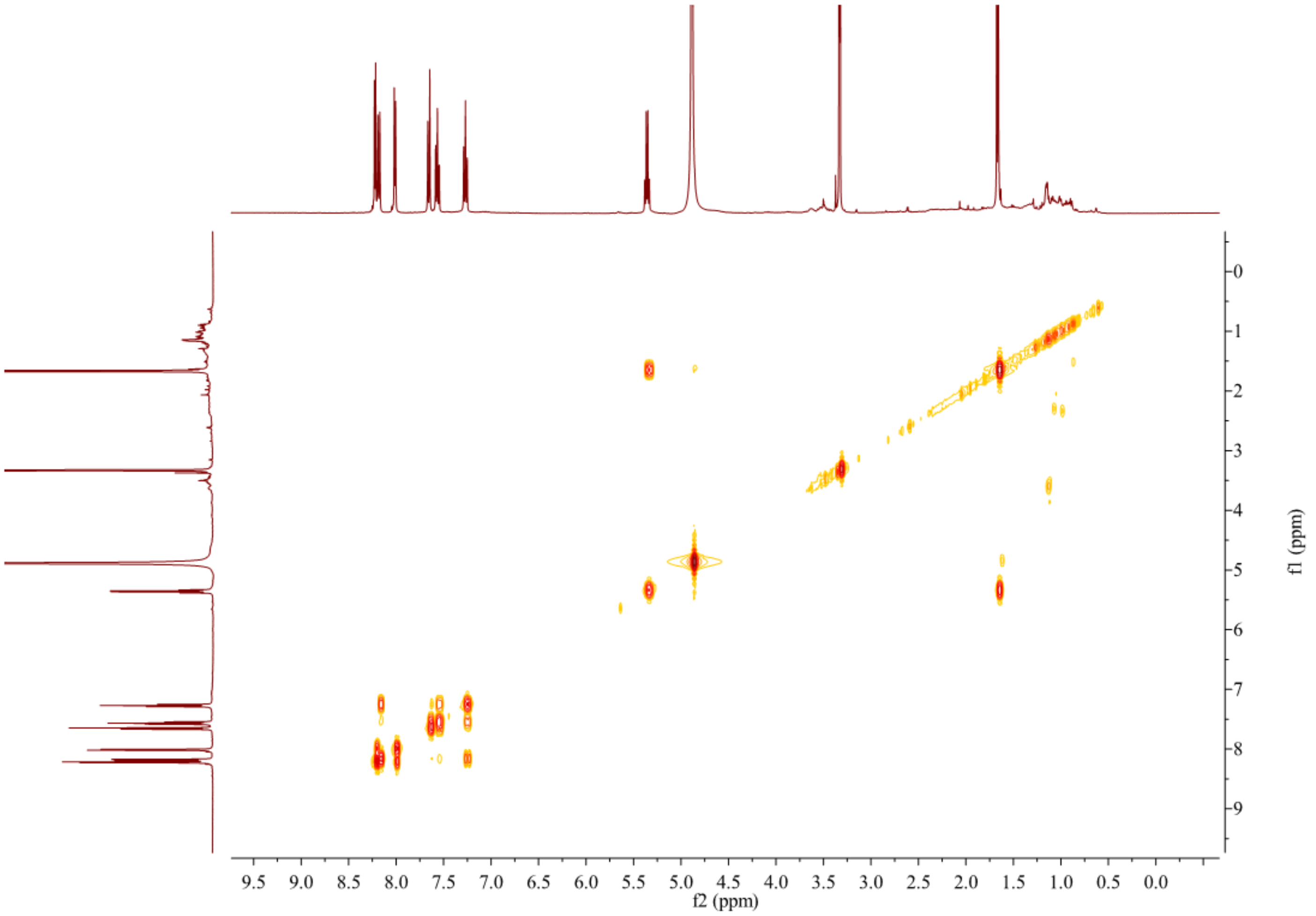

Figure S71. ^{1}H–^{1}H COSY spectrum of 1-(9*H*-pyrido [3,4-b] indol-1-yl) ethan-1-ol (**12**) in methanol-d_4.

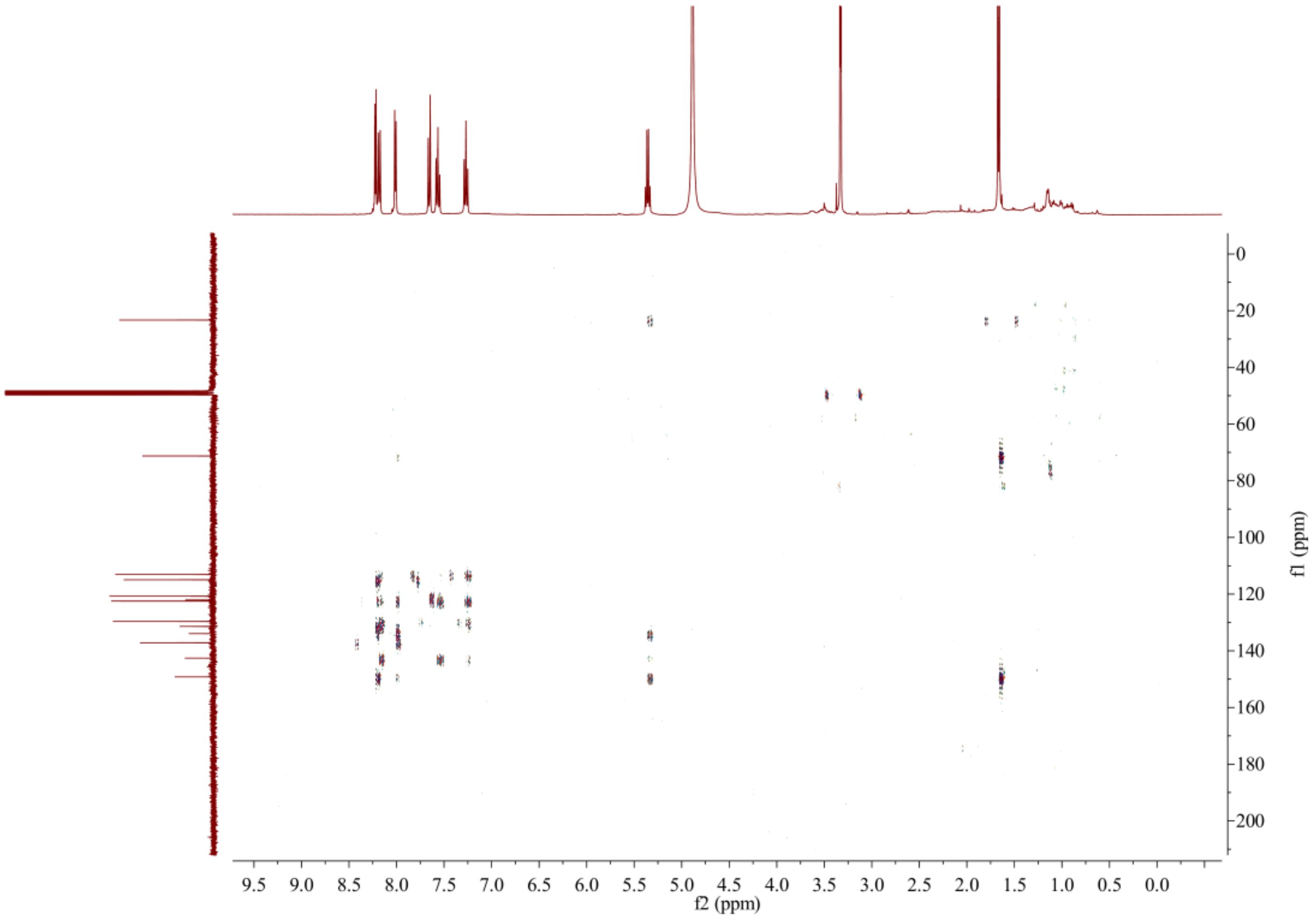

Figure S72. HMBC spectrum of 1-(9*H*-pyrido [3,4-b] indol-1-yl) ethan-1-ol (**12**) in methanol-d_4.

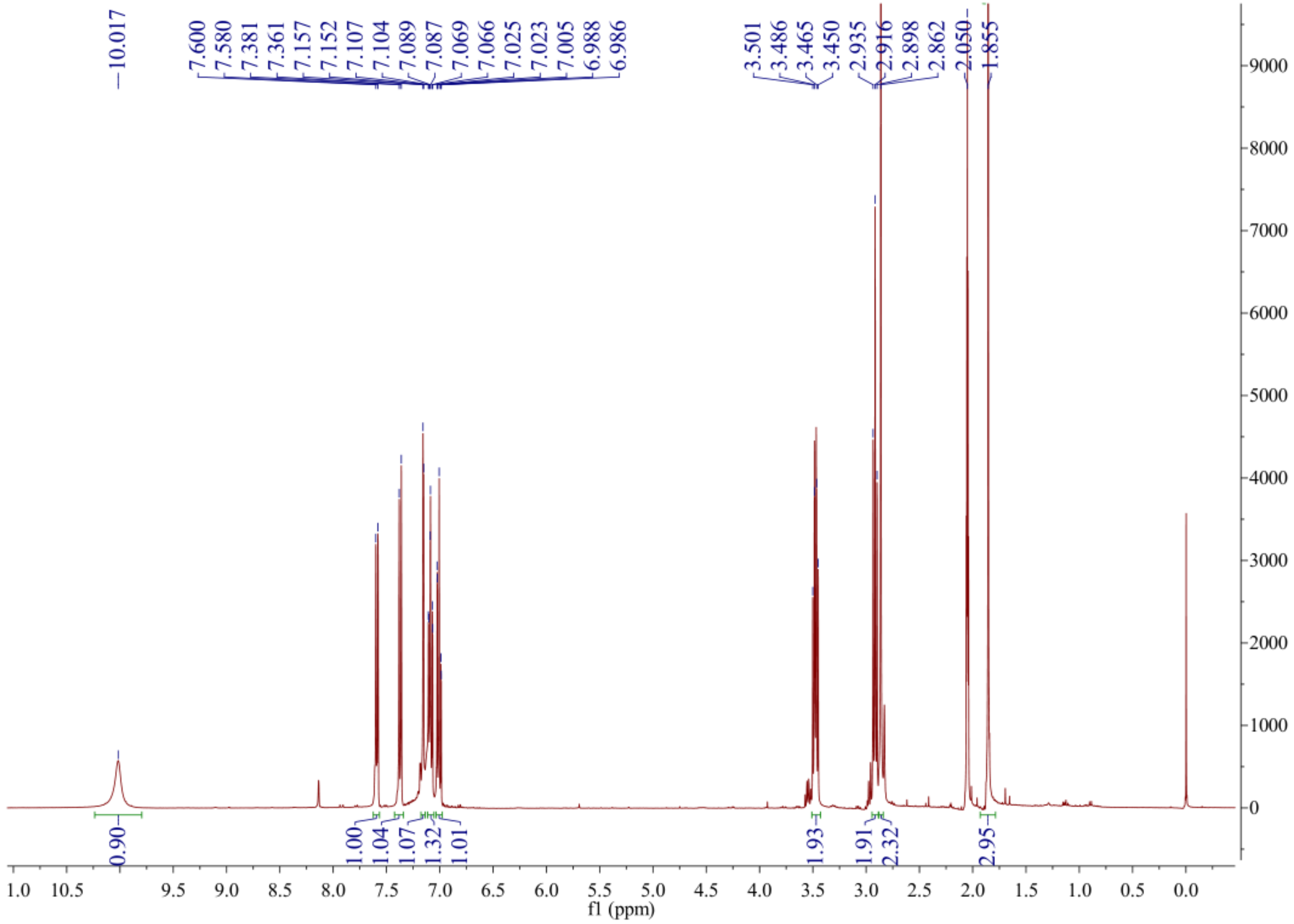

Figure S73. ^{1}H NMR spectrum of N_b-acetyltryptamine (**13**) in acetone-d_6 (400 MHz).

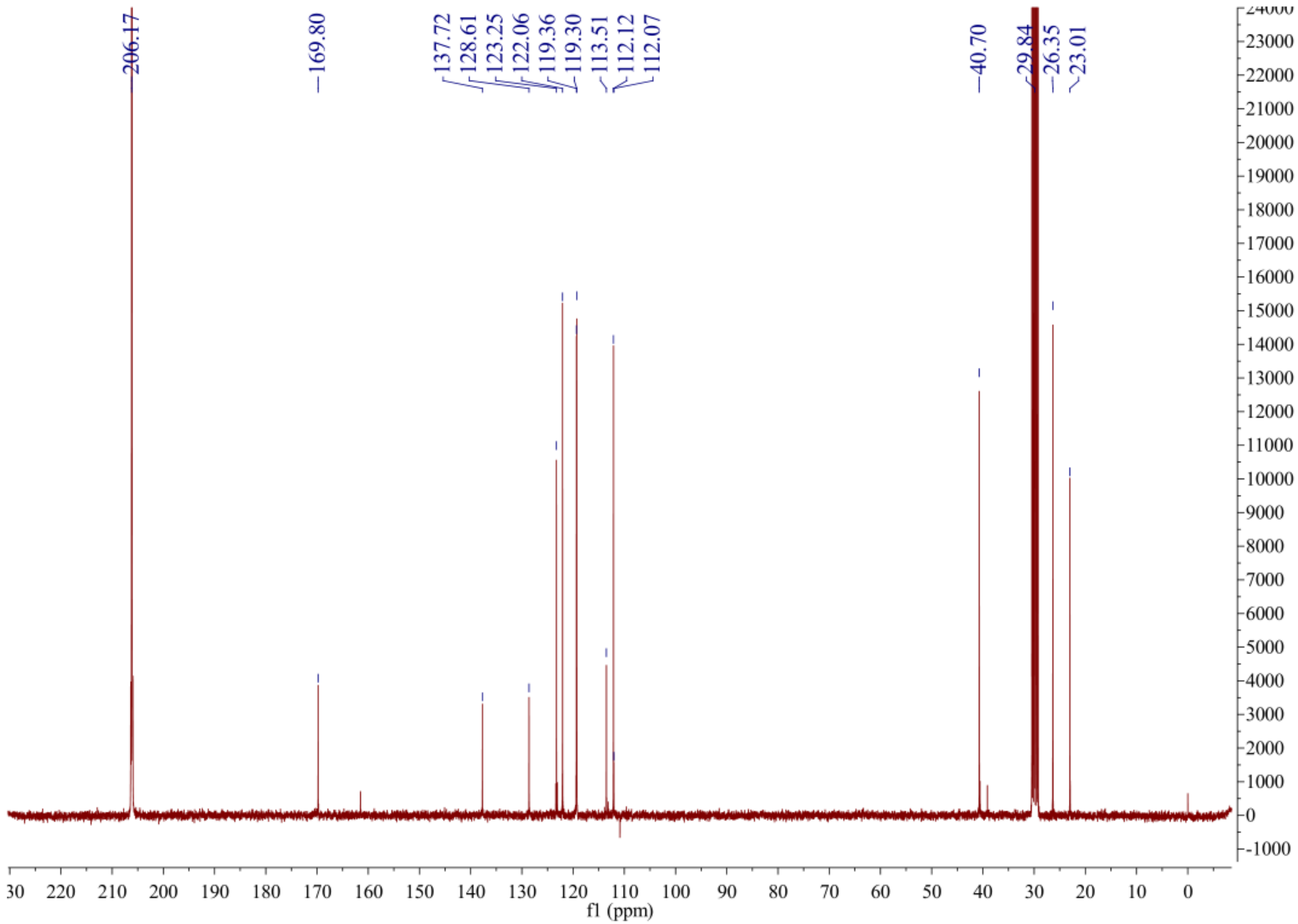

Figure S74. ^{13}C NMR spectrum of N_b-acetyltryptamine (**13**) in acetone-d_6 (100 MHz).
